# High Hemin Concentration Induces Escape from Senescence of Normoxic and Hypoxic Colon Cancer Cells

**DOI:** 10.3390/cancers14194793

**Published:** 2022-09-30

**Authors:** Agata Borkowska, Aleksandra Olszewska, Weronika Skarzynska, Marta Marciniak, Maciej Skrzeszewski, Claudine Kieda, Halina Was

**Affiliations:** 1 Laboratory of Molecular Oncology and Innovative Therapies, Military Institute of Medicine, Szaserow 128 Street, 04-141 Warsaw, Poland; 2 Postgraduate School of Molecular Medicine, Medical University of Warsaw, Zwirki i Wigury 61 Street, 02-091 Warsaw, Poland; 3 Doctoral School of Translational Medicine, Centre of Postgraduate Medical Education, 01-813 Warsaw, Poland; 4 Centre for Molecular Biophysics, UPR CNRS 4301, CEDEX 2, 45071 Orléans, France

**Keywords:** colon cancer, bleeding, bruise, chemotherapy, senescence, hemin, antioxidative enzymes, heme oxygenase-1, catalase, glutathione peroxidase-1, oxidative stress

## Abstract

**Simple Summary:**

High red-meat consumption as well as bleeding or bruising can promote oxidative stress and, in consequence, cancer development. However, the mechanism of that phenomenon is not understood. The induction of therapy-induced senescence (TIS) might also be induced by oxidative stress. Recently, TIS cells, despite their inhibited proliferation potential, have been identified as one of the sources of tumor re-growth. Here, with the use of molecular analyses, we found that oxidative stress, promoted by high doses of hemin or H_2_O_2_, can trigger TIS escape and cell re-population. It is closely related to the activity of antioxidative enzymes, especially heme oxygenase-1. Hypoxia might accelerate these effects. Therefore, we propose that the prevention of excessive oxidative stress could be a potential target in senolytic therapies.

**Abstract:**

Hemoglobin from either red meat or bowel bleeding may promote oxidative stress and increase the risk of colorectal cancer (CRC). Additionally, solid cancers or their metastases may be present with localized bruising. Escape from therapy-induced senescence (TIS) might be one of the mechanisms of tumor re-growth. Therefore, we sought to study whether hemin can cause escape from TIS in CRC. To induce senescence, human colon cancer cells were exposed to a chemotherapeutic agent irinotecan (IRINO). Cells treated with IRINO exhibited common hallmarks of TIS. To mimic bleeding, colon cancer cells were additionally treated with hemin. High hemin concentration activated heme oxygenase-1 (HO-1), induced escape from TIS and epithelial-to-mesenchymal transition, and augmented progeny production. The effect was even stronger in hypoxic conditions. Similar results were obtained when TIS cells were treated with another prooxidant agent, H_2_O_2_. Silencing of antioxidative enzymes such as catalase (CAT) or glutathione peroxidase-1 (GPx-1) maintained colon cancer cells in a senescent state. Our study demonstrates that a high hemin concentration combined with an increased activity of antioxidative enzymes, especially HO-1, leads to escape from the senescence of colon cancer cells. Therefore, our observations could be used in targeted anti-cancer therapy.

## 1. Introduction

Colorectal cancer is a major public health problem, being the third most commonly diagnosed cancer and the fourth cause of cancer-related death worldwide [1]. Epidemiologic factors related to an increased risk for CRC include old age, tobacco use, red meat consumption, obesity, and lack of physical activity [2]. The molecular and cellular mechanisms that are responsible for CRC development, associated with aging and diet, are not well understood. Regarding diet, it has been suggested that oxidative factors related to the high consumption of red meat might promote CRC formation [3]. Hemoglobin from either red meat or bowel bleeding may increase oxidative damage in the bowel and a higher risk of colorectal cancer [4,5]. Additionally, it was recently demonstrated that a high-fat, low-calcium, and vitamin D diet leads to oxidative stress in the colon [6]. It is also possible that the effects of the diet on the microbiome may affect CRC risk by elevating the microbiome-mediated oxidative stress in the colon [7]. However, oxidative stress is likely limited, if red meat and processed meat are consumed in moderation, and combined with high consumption of fruits, vegetables, antioxidants, and a low intake of refined sugars [8]. About aging, a variety of potential mechanisms have been proposed to mediate age-related disease, including the accumulation of increased oxidative stress injury, DNA damage, misprocessed protein aggregates, and cellular senescence [9]. Oxidative stress and reactive oxygen species (ROS) accumulate in aging tissue and can induce cellular senescence, which is believed to promote age-related diseases [9], including colorectal cancer [10].

Stress-induced premature senescence (SIPS) might be one of the drug-resistance mechanisms [11,12,13]. SIPS is an acute and short-term effect, which is independent of telomere erosion. It may be triggered by oxidative stress or DNA damaging agents (chemotherapeutics and radiotherapy), thus called therapy-induced senescence (TIS) [14]. Features of senescent cells include irreversible growth arrest, augmented size and granularity, activation of the DNA damage response (DDR) pathway, polyploidization, and elevated activity of the senescence-associated β-galactosidase (SA-β-Gal) and senescence-associated secretory phenotype (SASP) [15]. The induction of senescence may be related to anti-tumor effects, as it prevents most cancer cells from dividing. On the other hand, it could result in the development of resistance to therapy and cancer recurrence [16,17]. Recently, the accumulation of TIS cells has been linked to reduced survival of patients subjected to anticancer treatment [11,12,13,15,18,19]. This effect could be related to the remodeling of the tumor environment, mediated by SASP agents, including, e.g.,: growth factors, immunomodulatory cytokines, or proteases [15]. Changes in non-cancerous cells during aging may also contribute to a tumor permissive microenvironment [20,21]. Another explanation for that phenomenon might be an atypical division of senescent, polyploid cells, called neosis. Neosis is characterized by karyokinesis via nuclear budding followed by asymmetric, intracellular cytokinesis, producing several small mononuclear cells with an extended mitotic life span [22,23,24]. Recent studies suggest that cancer cells escape from senescence might be connected with cell polyploidization/depolyploidization [19,25,26,27,28,29,30,31,32]. Polyploidy is the result of endoreplication, a cell cycle variation that produces a polyploid genome by repeated rounds of DNA replication in the absence of cell division. Recent evidence suggests that endoreplication can confer genome instability, a major cancer-enabling property [33]. Additionally, it was suggested that tumor cells containing an elevated genomic content and resembling blastocyst might be key players in the evolution of cancer and resistance to therapies [27,34,35].

Here, we asked the question of whether antioxidative systems can play a role in the escape of cancer cells from senescence. We demonstrated that a high concentration of prooxidants hemin and H_2_O_2_ causes colon cancer cells escape from senescence, along with a highly proliferating progeny and epithelial-to-mesenchymal transition (EMT).

## 2. Results

### 2.1. High Hemin Concentration Causes Escape from Senescence

**Figure 1:**
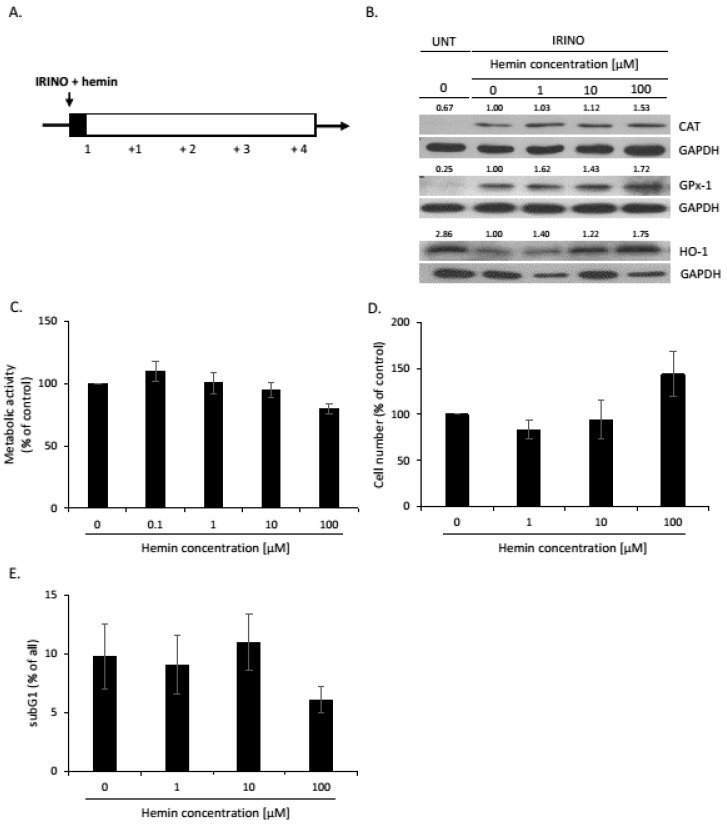
Hemin influences the activity of senescent HCT116 cells. (**A**) The scheme of the experiment. HCT116 colon cancer cells were subjected to 2.5 μM of irinotecan (IRINO) and 1, 10, or 100 μM of hemin. After 24 h, the medium was changed and cells were cultured in a drug-free medium for 4 days. (**B**) Expression of antioxidative enzymes in untreated (UNT) and IRINO-treated cells subjected to hemin in different concentrations. Representative blots show levels of catalase, GPx-1, and HO-1 proteins. GAPDH acts as loading control; (**C**) Evaluation of metabolic activity assayed by MTT assay; (**D**) Evaluation of cell number. Cell number was counted using Bürker’s chamber. (**E**) Quantification of the percentage of cells in the subG1 phase (with DNA content < 2C). Cell cycle analysis was performed with the use of Muse^TM^ Cell Cycle Reagent and flow cytometry. Each bar represents mean ± SEM, n ≥ 3.

**Figure A1:**
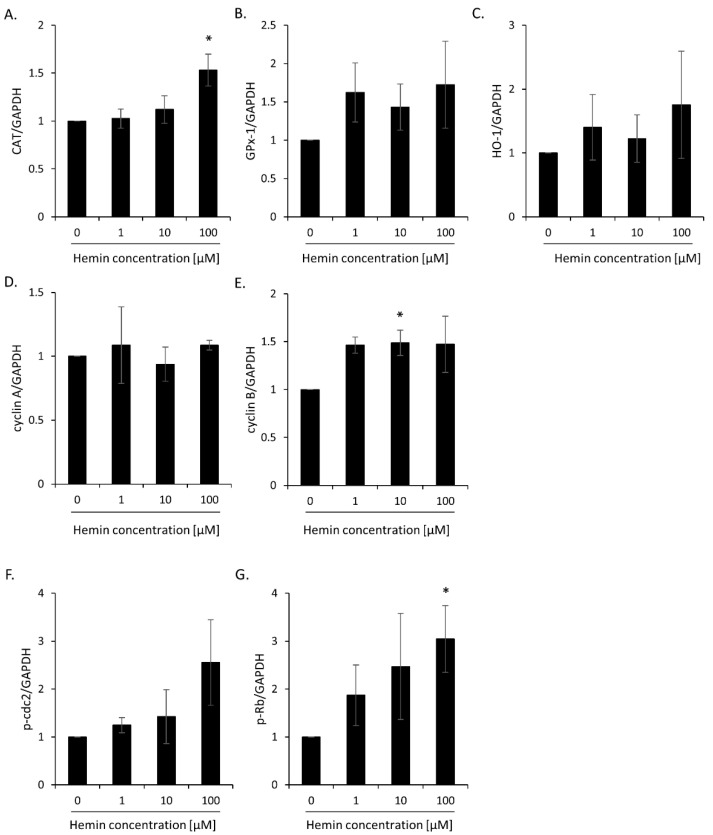
Evaluation of protein expression in HCT116 cells treated with 2.5 μM IRINO subjected to 1, 10, or 100 μM hemin: antioxidative and proliferation markers. Quantification is based on the densitometry analysis performed with the use of ImageJ software. Data are shown as the ratio of the protein amount to respective protein loading control (GAPDH): (**A**) CAT; (**B**) GPx-1; (**C**) HO-1; (**D**) cyclin A; (**E**) cyclin B; (**F**) p-cdc2; (**G**) p-Rb. Each bar represents mean ± SEM, n ≥ 3; * *p* < 0.05—hemin concentration vs. 0.

In our previous studies, we found that human colon cancer cell lines treated with various chemotherapeutics underwent senescence. However, after withdrawal of the drug, cell culture re-growth was observed [25,26,27,36]. The strongest induction of senescence was detected after irinotecan, the drug, that is commonly used in the therapy of colon cancer patients [27]. As the development of senescence might be mediated through reactive oxygen species (ROS) [25], here, we checked, whether antioxidative systems might interfere with the induction of senescence/escape from senescence. To induce senescence, HCT116 and SW480 colon cancer cells were treated with irinotecan (IRINO) and a series of hemin concentrations for 24 h. After, that medium was changed to drug-free one and cells were cultured for the next 4 days (Figure 1A). The induction of senescence was correlated with differential changes in the expression of antioxidative enzymes. The expression of CAT and GPx-1, enzymes involved in the reaction to H_2_O_2_ treatment, was elevated (Figure 1B and Figure A1A,B). In contrast, the expression of heme oxygenase-1 (HO-1), an enzyme that converts heme to biliverdin, CO, and iron ions, was reduced (Figure 1B and Figure A1C). To verify the effects of HO-1 on the induction of senescence, colon cancer cells were treated with hemin, as an HO-1 activator and substrate. In senescent cancer cells, hemin induced HO-1, CAT, and GPx-1 expression (Figure 1B and Figure A1A–C). Hemin, only at the highest dose of 100 µM, showed a strong tendency to decrease the metabolic activity of cancer cells (Figure 1C, *p* = 0.054; Figure A10A). It also showed the tendency to increase the total cell number (Figure 1D) but not the proportion of cells in the subG1 phase (Figure 1E and Figure A10B). These data suggest that hemin at high concentrations may affect the senescence process.

**Figure 2:**
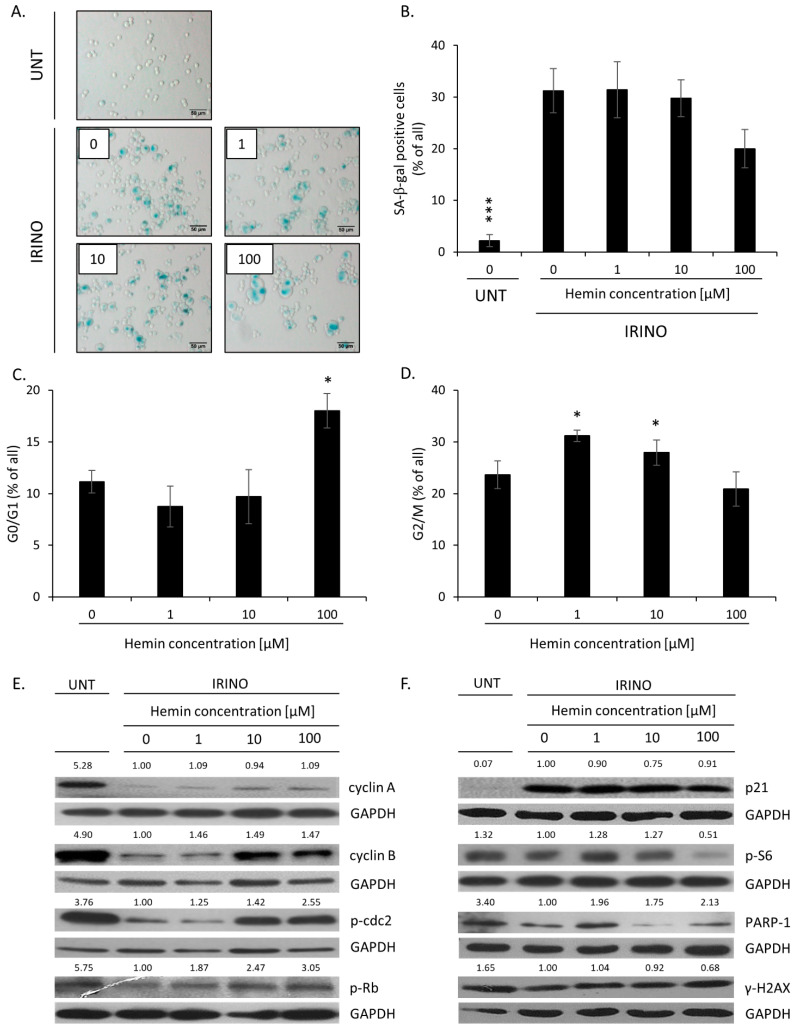
High hemin concentration correlates with an escape from senescence. (**A**) The activity of the SA-β-Gal enzyme in UNT and IRINO-treated cells subjected to hemin. Detection of the enzyme was performed on cytospined cells. Representative photos were acquired using light microscopy. Original magnification 200×, scale bar 50 μM; (**B**) Quantification of SA-β-Gal positive cells after treatment with hemin; (**C**,**D**) Quantification of the percentage of cells in the G0/G1 phase (**C**) and the G2/M phase (**D**). Cell cycle analysis was performed with the use of Muse^TM^ Cell Cycle Reagent and flow cytometry. (**E**) Expression of proteins related to cell cycle progression. Representative blots show the level of cyclin A, cyclin B, p-cdc2, and p-Rb in UNT cells and IRINO-treated cells subjected to different hemin concentrations. GAPDH acts as loading control. (**F**) Production of protein related to cell cycle inhibition, geroconversion, and DNA repair after treatment with hemin. Representative blots show levels of p21, p-S6, PARP-1, and γH2AX. GAPDH acts as a loading control. Each bar represents mean ± SEM, n ≥ 3; * *p* < 0.05, *** *p* < 0.001—hemin concentration vs. 0.

**Figure A2:**
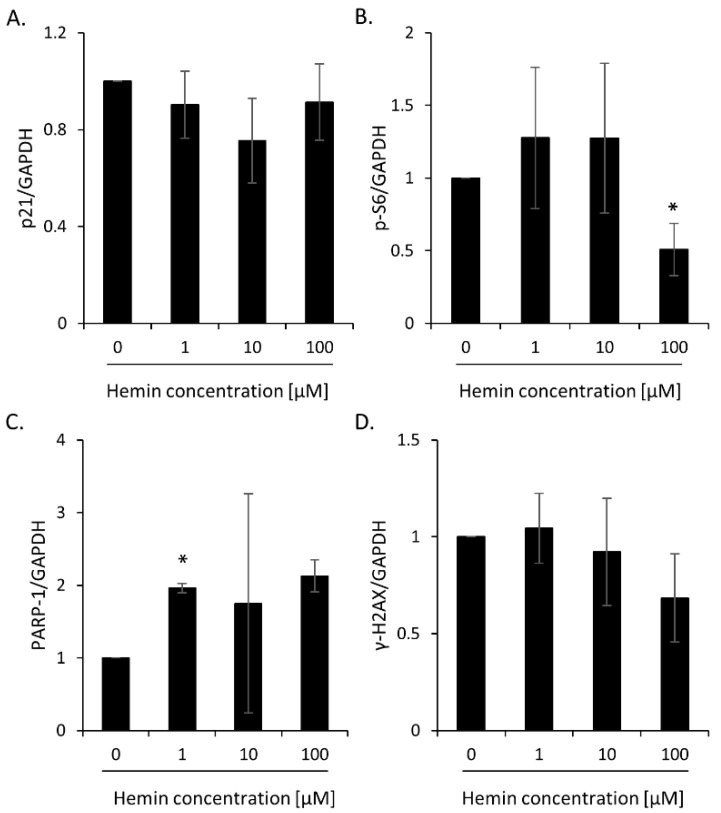
Evaluation of protein expression in HCT116 cells treated with 2.5 μM IRINO subjected to 1, 10, or 100 μM hemin: cell cycle inhibition and DNA repair markers. Quantification is based on the densitometry analysis performed with the use of ImageJ software. Data are shown as the ratio of the protein amount to respective protein loading control (GAPDH): (**A**) p21; (**B**) p-S6; (**C**) PARP-1; (**D**) γ-H2AX. Each bar represents mean ± SEM, n ≥ 3; * *p* < 0.05—hemin concentration vs. 0.

Hemin at 100 µM concentration tends to reduce the proportion of senescent cells, as shown by decreased numbers of SA-β-gal-positive cells (Figure 2A,B, *p* = 0.052; Figure A10C,D). It correlated with higher proportions of cells in the G0/G1 phase (Figure 2C and Figure A10E). Recently, we showed that such an alteration in cell cycle distribution might be related to amitotic divisions of senescent cancer cells and the appearance of a small, highly proliferating progeny [27]. In contrast, the proportion of cells accumulated in the G2/M phase was reduced (Figure 2D and Figure A10F). There were no changes in the proportion of senescent HCT116 cells accumulated in the S (Figure A11A) or polypoidal phases upon hemin treatment (Figure A11B). In the case of SW480 cells, a tendency toward a reduction in cells present in the S (Figure A11C, *p* = 0.056) and polypoidal phases was observed (Figure A11D). To confirm that a high concentration of hemin induced escape from senescence, the expression of proteins involved in proliferation and senescence was checked. The levels of proteins related to the cell cycle progression—cyclin A, cyclin B, p-cdc2, and p-Rb—were downregulated in IRINO-treated cells but upregulated when senescent cells were treated with 100 µM of hemin (Figure 2E and Figure A1D–G). In this vein, the expression of two proteins related to cell cycle inhibition and geroconversion—p21 and p-S6—was reduced (Figure 2F and Figure A2A,B). The expression of PARP-1, a protein responsible for DNA repair, was upregulated in hemin-treated senescent cancer cells (Figure 2F and Figure A2C), whereas the expression of γ-H2AX, the sensor of DNA double breaks, showed a tendency to reduce (Figure 2F and Figure A2D). Accordingly, the presence of the cleaved form of PARP-1, indicative of the ongoing apoptosis process, was not detected (Figure 2F and Figure A2C).

Altogether, these data showed that, in contrast to CAT and GPx-1, HO-1 expression was downregulated upon the induction of senescence in colon cancer cells. A high concentration of hemin, the HO-1 substrate, and the activator most likely promoted escape from senescence. It was marked by the appearance of SA-β-gal negative, highly proliferating cells, progressing through the G0/G1 phase, and a shift from senescence to proliferation molecular markers.

### 2.2. High H_2_O_2_ Level Leads to Escape from Senescence and Induces EMT

**Figure 3:**
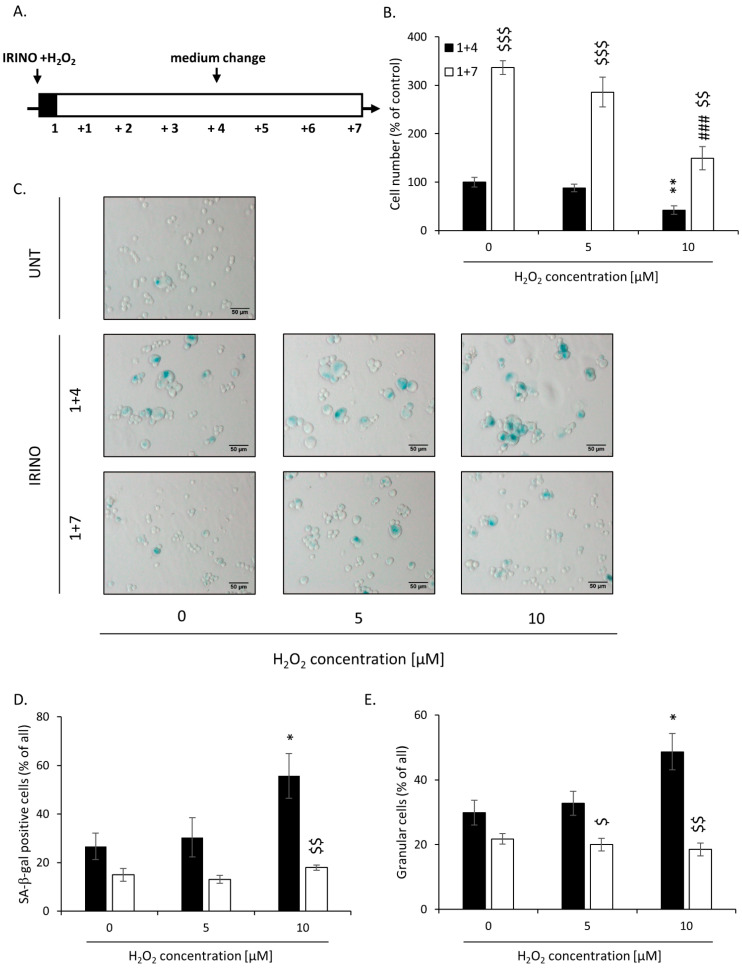
High H_2_O_2_ leads to escape from senescence at day 1 + 7: increased cell number but decreased SA-β-Gal and granularity. (**A**) The scheme of the experiment. Cells were subjected to 2.5 μM of irinotecan and 5 or 10 μM H_2_O_2_. After 24 h, the medium was changed and the cells were cultured in a drug-free medium for 4 (■) or 7 (□) days. In a 7-day long variant of the experiment, after 4 days of cell culture, the medium was changed to the new one. (**B**) Evaluation of cell number on the 4th and 7th days after the treatment. (**C**) Activity of SA-β-gal enzyme in UNT and IRINO–treated cells after treatment with H_2_O_2_. Cells were cytospined, and cytochemical staining for SA-β-gal activity was performed. Representative photos were acquired using light microscopy. Original magnification 200×, scale bar 50 μM. (**D**) Quantification of SA-β-gal positive cells on the 4th and 7th days after the treatment. (**E**) Percentages of granular cells as determined by FSC/SSC analysis using flow cytometry. Each bar represents mean ± SEM, n ≥ 3; * *p* < 0.05, ** *p* < 0.01—vs. 1 + 4 control; ### *p* < 0.001—vs. 1 + 7 control; $ *p* < 0.05, $$ *p* < 0.01, $$$ *p* < 0.001—1 + 7 vs. 1 + 4.

As high hemin concentration results in increased oxidative stress [37,38], we tested whether the prooxidant, H_2_O_2_, would lead to escape from senescence, too. HCT116 cells were incubated with 5 or 10 μM H_2_O_2_ and IRINO for 24 h and then cultured in a drug-free medium for the next 4 (“1 + 4” protocol) or 7 (“1 + 7” protocol) days (Figure 3A). H_2_O_2_ at the 10 μM concentration caused a significant decrease in cell numbers at day 1 + 4 (Figure 3B). It correlated with elevated numbers of SA-β-gal positive (Figure 3C,D) and granular cells (Figure 3E). At day 1 + 7, upon senescence escape, the percentages of SA-β-gal and granular cells were significantly reduced (Figure 3C–E).

**Figure 4:**
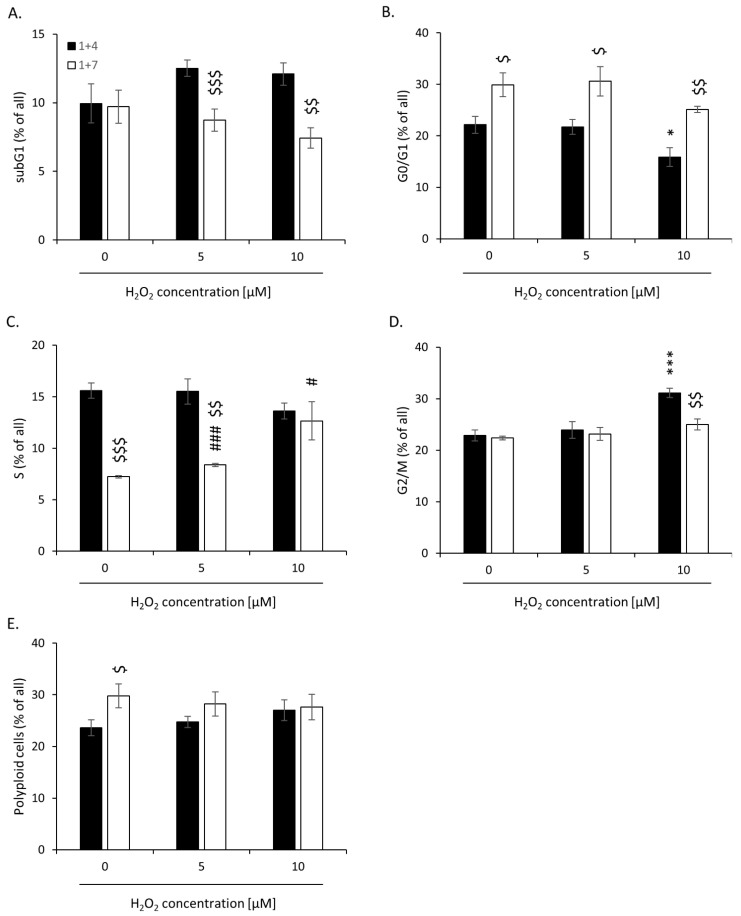
High H_2_O_2_ concentration leads to escape from senescence: changed accumulation in different phases of the cell cycle. Cells were subjected to 2.5 μM of irinotecan and 5 or 10 μM H_2_O_2_. After 24 h, the medium was changed and the cells were cultured in a drug-free medium for 4 (■) or 7 (□) days. In a 7-day long variant of the experiment, after 4 days of cell culture, the medium was changed to the new one. (**A**) subG1 (with DNA content < 2c); (**B**) G0/G1; (**C**) S; (**D**) G2/M. (**E**) Percentages of polyploid cells. Cell cycle analysis was performed using PI staining and flow cytometry. Each bar represents mean ± SEM, n ≥ 3; * *p* < 0.05, *** *p* < 0.001—vs. 1 + 4 control; # *p* < 0.05, ### *p* < 0.001—vs. 1 + 7 control; $ *p* < 0.05, $$ *p* < 0.01, $$$ *p* < 0.001—1 + 7 vs. 1 + 4.

Moreover, on day 1 + 4, H_2_O_2_-treated senescent cells showed reduced proportion in the G0/G1 phase of the cell cycle (Figure 4B) and an augmented accumulation in the G2/M phase (Figure 4D). On day 1 + 7, upon senescence escape, the effects were opposites (Figure 4B,D). There was also a significant decrease in the proportion of H_2_O_2_-treated cells accumulated in the subG1 phase between days 1 + 7 and 1 + 4 (Figure 4A). Escape from senescence correlated with an increased proportion of cells in the S phase, which was the most visible in cells treated with the highest H_2_O_2_ dose (Figure 4C). There were no changes in the proportion of polyploid cells (Figure 4E). These data suggest that increased oxidative stress related to high H_2_O_2_ dose promotes an escape from senescence.

**Figure 5:**
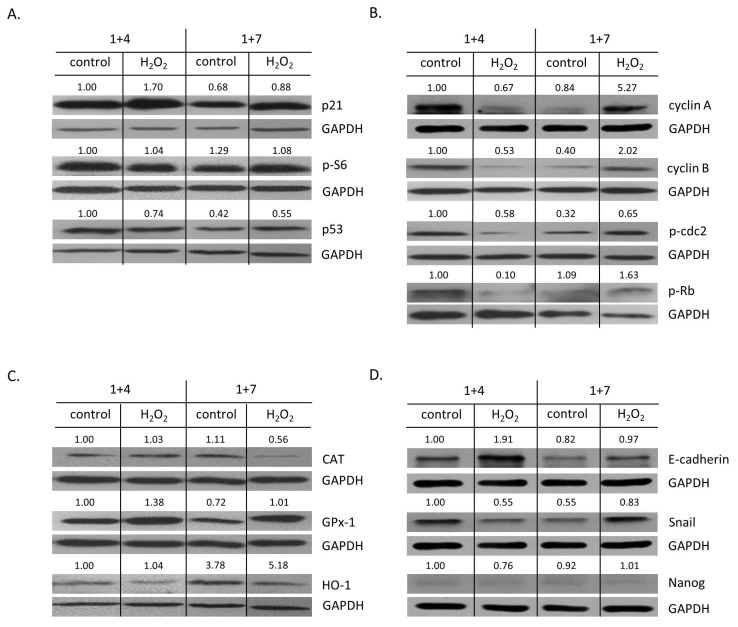
High H_2_O_2_ level leads to escape from senescence: changed expression of proteins related to proliferation and EMT. Cells were subjected to 2.5 μM of IRINO and 5 or 10 μM H_2_O_2_. After 24 h, the medium was changed and the cells were cultured in a drug-free medium for 4 (■) or 7 (□) days. In a 7-day long variant of the experiment, after 4 days of cell culture, the medium was changed to the new one. (**A**) Production of protein related to cell cycle inhibition, geroconversion, and tumor suppression. Representative blots showing levels of p21, p-S6, and p53 proteins. (**B**) Production of proteins related to cell cycle progression. Representative blots show levels of cyclin A, cyclin B, p-cdc2, and p-Rb. (**C**) Production of antioxidative enzymes. Representative blots show levels of catalase, GPx-1, and HO-1 proteins. (**D**) Production of proteins related to EMT and stemness. Representative blots show levels of E-cadherin, Snail, and Nanog. GAPDH acts as a loading control.

**Figure A3:**
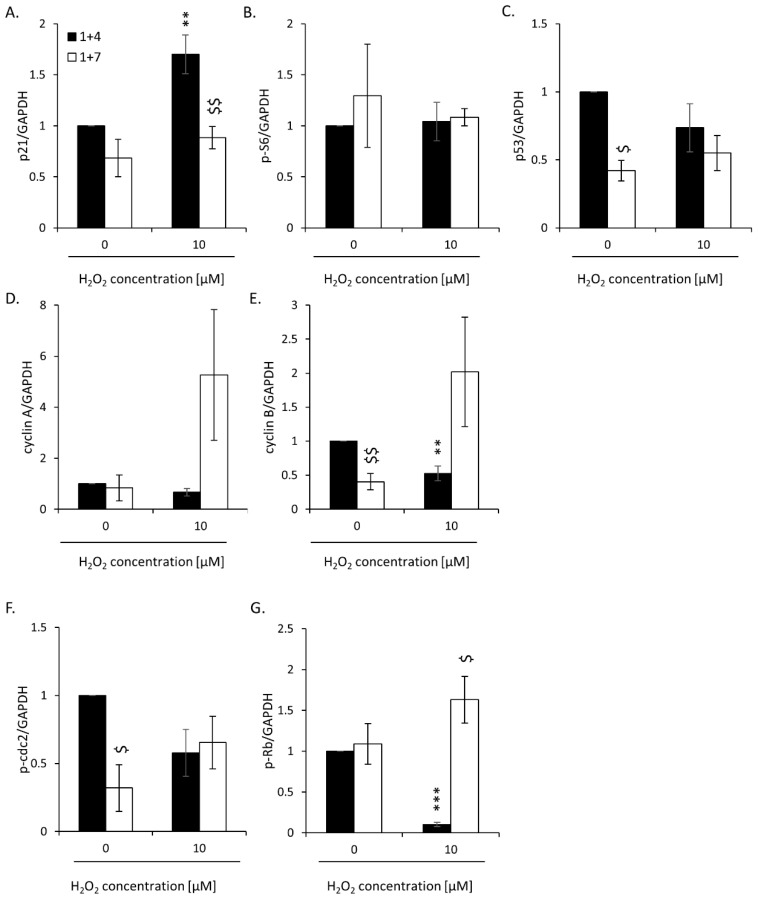
Evaluation of protein expression in HCT116 cells treated with 2.5 μM IRINO subjected to 5 or 10 μM H_2_O_2_ after the 4th and 7th days of the experiment: cell cycle inhibition and proliferation markers. Quantification is based on the densitometry analysis performed with the use of ImageJ software. Data are shown as the ratio of the protein amount to respective protein loading control (GAPDH): (**A**) p21; (**B**) p-S6; (**C**) p53; (**D**) cyclin A; (**E**) cyclin B; (**F**) p-cdc2; (**G**) p-Rb. Each bar represents mean ± SEM, n ≥ 3; ** *p* < 0.01, *** *p* < 0.001—vs. 1 + 4 control; $ *p* < 0.05, $$ *p* < 0.01—1 + 7 vs. 1 + 4.

**Figure A4:**
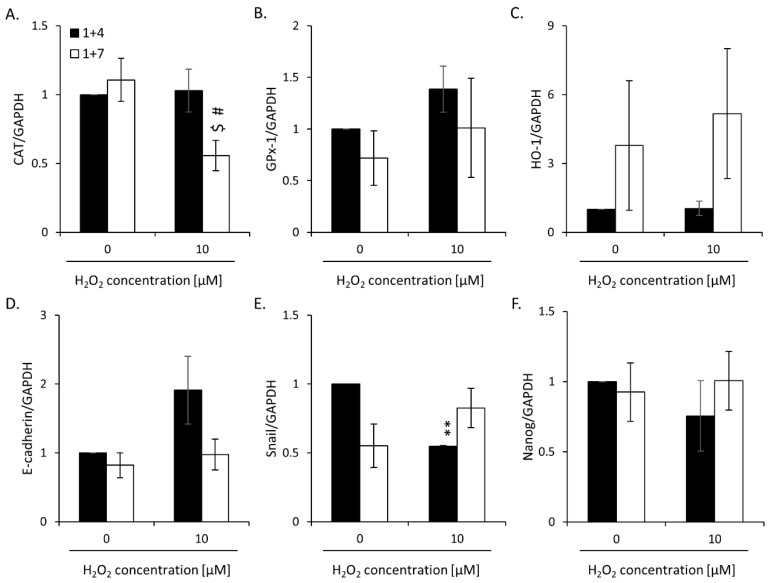
Evaluation of protein expression in HCT116 cells treated with 2.5 μM IRINO subjected to 5 or 10 μM H_2_O_2_ after the 4th and 7th days of the experiment: antioxidative and EMT markers. Quantification is based on the densitometry analysis performed with the use of ImageJ software. Data are shown as the ratio of the protein amount to respective protein loading control (GAPDH): (**A**) CAT; (**B**) GPx-1; (**C**) HO-1; (**D**) E-cadherin; (**E**) Snail; (**F**) Nanog. Each bar represents mean ± SEM, n ≥ 3; ** *p* < 0.01 —vs 1 + 4 control; # *p* < 0.05—vs 1 + 7 control; $ *p* < 0.05—1 + 7 vs. 1 + 4.

To verify these observations, the expression of proteins related to senescence and proliferation was tested. On day 1 + 4, the expression of a senescent marker, p21 (Figure 5A and Figure A3A), was elevated, whereas the expression of the proliferation markers—cyclin A (tendency), cyclin B, p-cdc2 (tendency), and p-Rb—was downregulated (Figure 5B and Figure A3D–G). On day 1 + 7, upon senescence escape, the effect was the opposite (Figure 5A,B and Figure A3A,D–G). There were no changes in p53 and p-S6 expression (Figure 5A and Figure A3B,C). HO-1 expression was elevated upon escaping from senescence. The effect was even stronger when senescent cells were treated with H_2_O_2_ (Figure 5C and Figure A4C). In contrast, upon senescence escape, H_2_O_2_-treated cells exhibited a reduced expression of CAT (Figure 5C and Figure A4A), whereas GPx-1 expression did not change (Figure 5C and Figure A4B). As we showed previously, escaping from senescence and progeny production might be related to acquiring a stem cell phenotype and epithelial-to-mesenchymal transition (EMT) [26,27]. In line with these data, senescent HCT116 cells treated with H_2_O_2_ underwent mesenchymal-to-epithelial transition (MET): at day 1 + 4, they exhibited augmented expression of E-cadherin (Figure 5D and Figure A4D) and reduced expression of its inhibitor, Snail (Figure 5D and Figure A4E). At day 1 + 7, when cells started to proliferate, the opposite process (EMT) was observed (Figure 5D and Figure A4D,E). There were no changes in the expression of a stem cell marker, Nanog (Figure 5D and Figure A4F).

To summarize, these data suggest that increased oxidative stress due to a high H_2_O_2_ concentration, similarly to high hemin concentration, triggers an escape from senescence, the appearance of highly proliferating progeny, a shift in HO-1/CAT expressions, and EMT.

### 2.3. Silencing of Antioxidative Enzymes Maintains Colon Cancer Cells in a Senescent State

**Figure 6:**
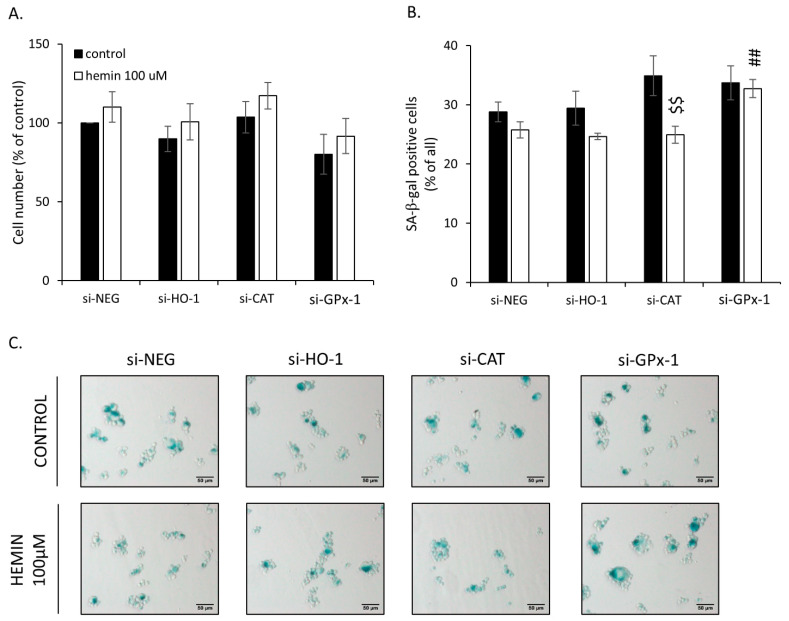
Silencing of antioxidative enzymes maintains colon cancer cells in a senescent state: changed proportion of SA-β-Gal positive cells. In HCT116 cells, genes encoding antioxidative enzymes—HO-1, CAT, or GPx-1—were silenced with the use of siRNAs. Cells were exposed to IRINO and 100 μM hemin (□). Control cells were subjected to IRINO and NaOH treatment (■). Cells were incubated with drugs for 24 h and then cultured in a drug-free medium for 4 days. (**A**) Evaluation of cell number. Cell number was counted with the use of Bürker’s chamber; (**B**) Evaluation of SA-β-gal positive cells. (**C**) Activity of SA-β-gal enzyme. Cells were cytospined and cytochemical stained for SA-β-gal activity. Representative photos were acquired using light microscopy. Original magnification 200×, scale bar 50 μM; Each bar represents mean ± SEM, n ≥ 3; ## *p* < 0.01—vs. si-NEG hemin 100 μM; $$ *p* < 0.01—control vs. hemin 100 μM.

To assess the possible molecular mechanisms responsible for the observed changes, HO-1, CAT, and GPx-1 were silenced with specific siRNAs. After gene silencing, colon cancer cells were treated with IRINO and a 100 µM concentration of hemin. GPx-1 knock-out slightly decreased the total cell number (Figure 6A) and increased the numbers of SA-β-gal positive cells in the presence of hemin (Figure 6B,C). Hemin also reduced the subpopulation of SA-β-gal positive cells among CAT-silenced ones (Figure 6B,C). HO-1 silencing did not affect cell number (Figure 6A) or the proportion of SA-β-gal positive cells (Figure 6B,C).

**Figure 7:**
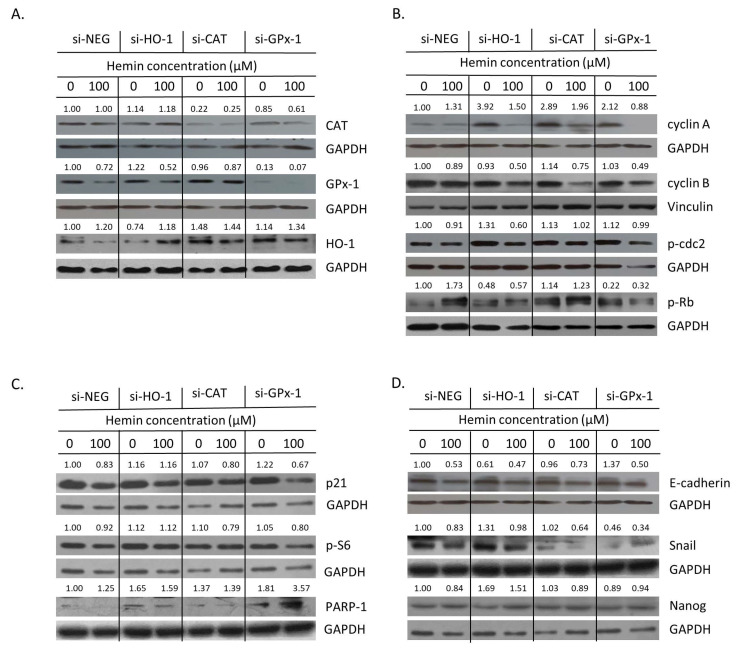
Silencing of antioxidative enzymes maintains colon cancer cells in a senescent state: changed expression of proteins related to proliferation and EMT. In HCT116 cells, genes encoding antioxidative enzymes—HO-1, CAT, and GPx-1—were silenced with the use of siRNAs. Cells were exposed to IRINO and 100 μM hemin (□). Control cells were subjected to IRINO and NaOH treatment (■). Cells were incubated with drugs for 24 h, then cultured in a drug-free medium for 4 days. (**A**) Expression of antioxidative enzymes. Representative blots show levels of catalase, GPx-1, and HO-1 proteins. (**B**) Expression of proteins related to cell cycle progression. Representative blots show levels of cyclin A, cyclin B, p-cdc2, and p-Rb. (**C**) Expression of proteins related to cell cycle inhibition, geroconversion, and tumor suppression. Representative blots show levels of p21, p-S6, and p53 proteins. (**D**) Expression of proteins related to EMT and stemness. Representative blots show levels of E-cadherin, Snail, and Nanog. GAPDH or vinculin acts as a loading control.

**Figure A5:**
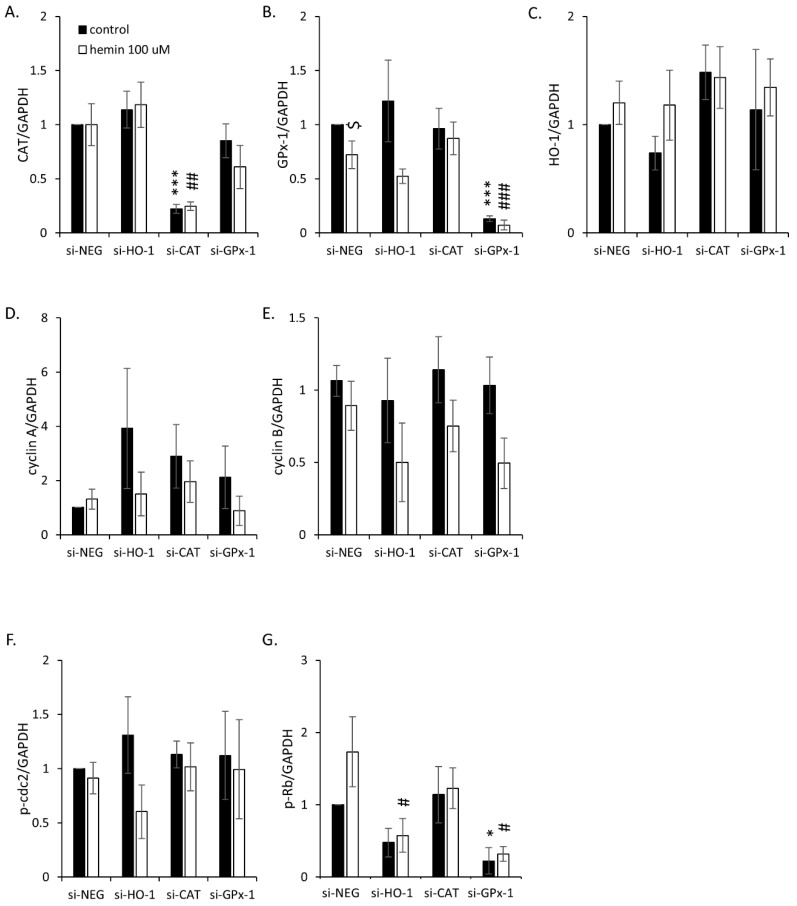
Evaluation of the protein expression in HCT116 cells, where genes encoding antioxidative enzymes HO-1, CAT, and GPx-1 were silenced with the use of siRNAs: antioxidative and proliferation markers. Cells were treated with 2.5 μM IRINO and exposed to 100 μM hemin. Control cells were subjected to IRINO and NaOH. Cells were incubated with drugs for 24 h; after that time, the medium was removed and the cells were cultured in a drug-free medium for 4 days. Quantification is based on the densitometry analysis performed with the use of ImageJ software. Data are shown as the ratio of the protein amount to respective protein loading control (GAPDH): (**A**) CAT; (**B**) GPx-1; (**C**) HO-1; (**D**) cyclin A; (**E**) cyclin B; (**F**) p-cdc2; (**G**) p-Rb. Each bar represents mean ± SEM, n ≥ 3; * *p* < 0.05, *** *p* < 0.001—vs. siNEG control; # *p* < 0.05, ## *p* < 0.01, ### *p* < 0.001—vs. siNEG hemin 100 μM; $ *p* < 0.05—control vs. hemin 100 μM.

**Figure A6:**
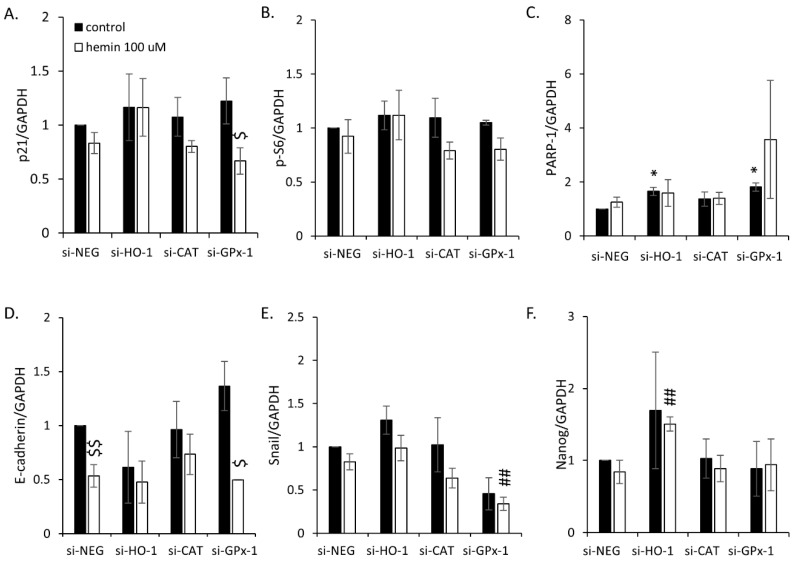
Evaluation of the protein expression in HCT116 cells, where genes encoding antioxidative enzymes HO-1, CAT, and GPx-1 were silenced with the use of siRNAs: cell cycle inhibition and EMT markers. Cells were treated with 2.5 μM IRINO and exposed to 100 μM hemin. Control cells were subjected to IRINO and NaOH. Cells were incubated with drugs for 24 h; after that time, the medium was removed and the cells were cultured in a drug-free medium for 4 days. Quantification is based on the densitometry analysis performed with the use of ImageJ software. Data are shown as the ratio of the protein amount to respective protein loading control (GAPDH): (**A**) p21; (**B**) p-S6; (**C**) PARP-1; (**D**) E-cadherin; (**E**) Snail; (**F**) Nanog. Each bar represents mean ± SEM, n ≥ 3; * *p* < 0.05—vs. siNEG control; ## *p* < 0.01—vs. siNEG hemin 100 μM; $ *p* < 0.05, $$ *p* < 0.01—control vs. hemin 100 μM.

Then, the expression of proteins related to oxidative stress, cell proliferation, senescence, EMT, and stemness was checked. Firstly, successful silencing of CAT and GPx-1 was confirmed (Figure 7A and Figure A5A,B). In the case of HO-1, gene knock-out was not entirely effective and the expression of HO-1 was further induced in response to hemin (Figure 7A and Figure A5C). This can explain why no significant effects of HO-1 silencing on cell number (Figure 6A) or proportion of SA-β-gal positive cells were observed (Figure 6B,C). Nevertheless, HO-1 silencing led to a reduced expression of proliferation marker p-Rb, regardless of hemin presence or absence (Figure 7B and Figure A5G). It was accompanied by elevated levels of the DNA repair protein PARP-1 (Figure 7C and Figure A6C) and the mesenchymal marker Snail (Figure 7D and Figure A6E). Upon hemin addition, Snail expression was downregulated (Figure 7D and Figure A6E), whereas the level of the stem cell marker Nanog was augmented (Figure 7D and Figure A6F). The silencing of CAT or GPx-1 showed a tendency to decrease the expression of proliferation marker cyclin B in the presence of hemin (Figure 7B and Figure A5E, *p* = 0.057). A loss of GPx-1 also reduced the expression of p-Rb, another proliferation marker, both in the presence and absence of hemin (Figure 7B and Figure A5G), while PARP-1 expression was augmented (Figure 7C and Figure A6C). It also caused MET, as an expression of E-cadherin, to be upregulated, whereas the expression of Snail was downregulated (Figure 7D and Figure A6D,E). In the presence of hemin, it seemed that the opposite process of EMT was started, as E-cadherin expression was reduced (Figure 7D and Figure A6D). It was correlated with a decreased expression of the cell cycle inhibitor p21 (Figure 7C and Figure A6A). Snail expression was decreased in both CAT- and GPx-1-silenced cells treated with hemin (Figure 7D and Figure A6E).

Taken together, these data suggest that the silencing of antioxidative enzymes—HO-1, CAT, and GPx-1—maintains colon cancer cells in a non-proliferating, epithelial state with increased mechanisms of DNA protection. Upon hemin addition, cells started to show features of cancer stem cells and escape from senescence.

### 2.4. Hemin Enhances Escape from Senescence in Hypoxic Cells in the Presence of CAT

As shown previously in colon and lung cancer cells, hypoxia may significantly affect the response of cancer cells to chemotherapeutics, including the development of senescence/escaping from it [39]. Therefore, the role of hypoxia on senescence escaping, upon modulation of oxidative status, was tested here.

**Figure 8:**
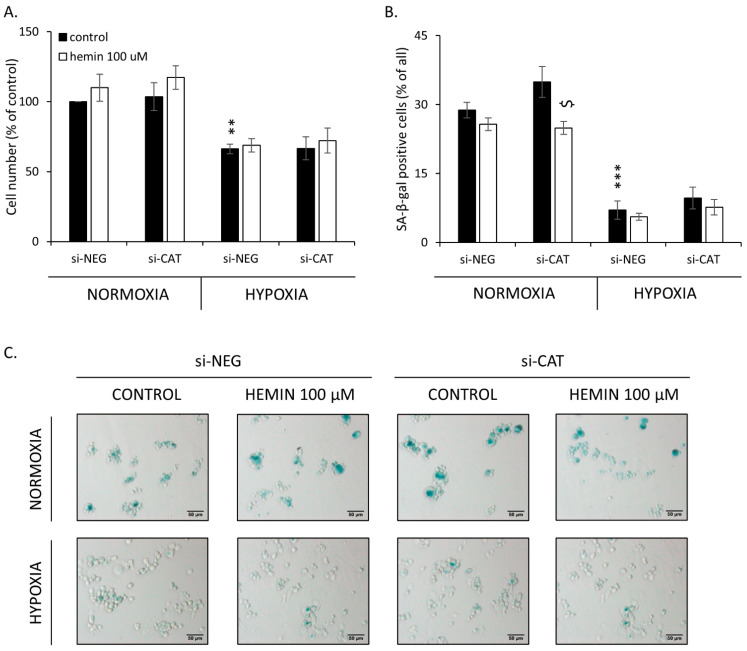
Hemin enhances escaping from senescence in hypoxic cells. In HCT116 cells, the gene encoding CAT was silenced with the use of siRNAs. Cells were cultured in parallel in normoxia or hypoxia. Cells were exposed to 100 μM hemin (□). Control cells were subjected to IRINO and NaOH in the corresponding dose (■). (**A**) Evaluation of cell number. Cell number was counted with the use of Bürker’s chamber. (**B**) Evaluation of SA-β-gal positive cells; (**C**) Activity of SA-β-gal enzyme. Cells were cytospined and cytochemical staining for SA-β-gal activity was performed. Representative photos were acquired using light microscopy. Original magnification 200×, scale bar 50μM; Each bar represents mean ± SEM, n ≥ 3; ** *p* < 0.01, *** *p* < 0.001—vs. si-NEG control normoxia; $ *p* < 0.05—control vs. hemin 100 μM.

Low oxygen tension significantly decreased the total cell number (Figure 8A) and the proportion of SA-β-gal positive cells in the HCT116 cell line (Figure 8B,C). In SW480 cells, hypoxia also decreased total cell number, whereas hemin application increase its rate (Figure A12A). However, hypoxia did not change the proportion of SA-β-gal positive cells in SW480 cells (Figure A12B,C). We previously showed that escaping from senescence is a multistep process that depends on the cell line, drug type and its concentration, the timing of the experiment, etc. We proposed that it can be monitored using different cellular and molecular markers [26,27,39]. In this vein, SW480 cells demonstrated a reduced accumulation in the subG1 (Figure A13A) and S phases (Figure A13D), as well as progression from the G2/M to G0/G1 phases upon hemin treatment (Figure A13B,C). There were no changes in the polyploid phase (Figure A13E). The effects were more pronounced in hypoxia (Figure A13).

**Figure 9:**
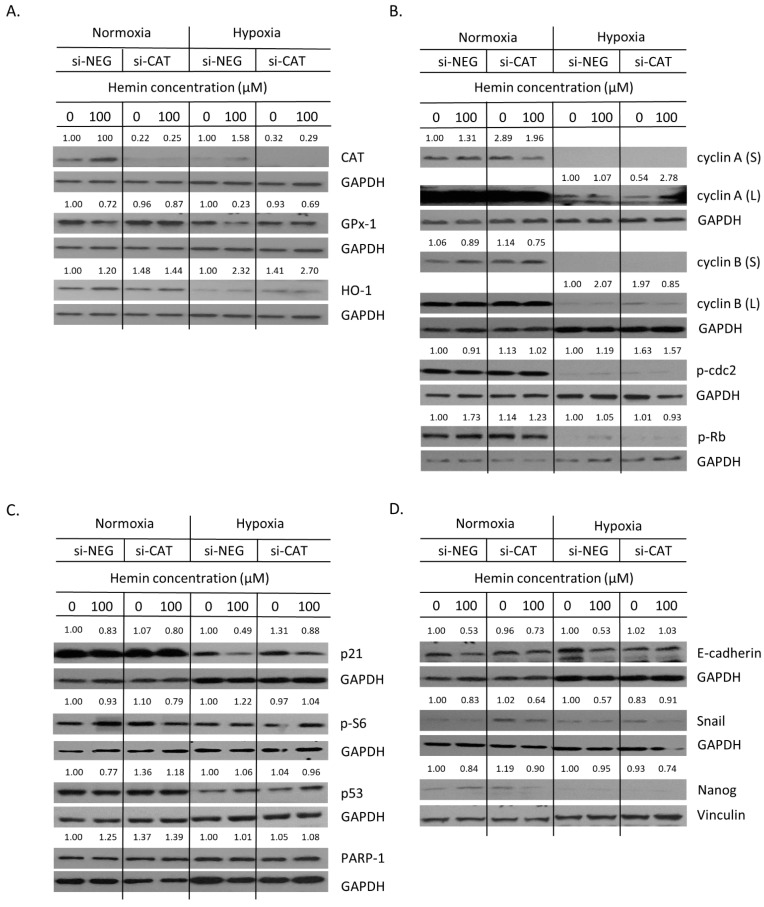
Hemin enhances escape from senescence in hypoxic cells in the presence of CAT: changed the expression of proteins related to proliferation and EMT. In HCT116 cells, the gene encoding CAT was silenced with the use of siRNAs. Cells were cultured in parallel in normoxia or hypoxia. Cells were exposed to 100 μM hemin (□). Control cells were subjected to IRINO and NaOH in the corresponding dose (■). (**A**) Production of antioxidative enzymes. Representative blots show levels of CAT, GPx-1, and HO-1 proteins. (**B**) Production of proteins related to cell cycle progression. Representative blots show levels of cyclin A, cyclin B, p-cdc2, and p-Rb. (**C**) Production of protein related to cell cycle inhibition, geroconversion, and tumor suppression. Representative blots show levels of p21, p-S6, p53, and PARP-1 proteins. (**D**) Production of protein related to EMT and stemness. Representative blots show levels of E-cadherin, Snail, and Nanog. GAPDH or vinculin acts as a loading control.

**Figure A7:**
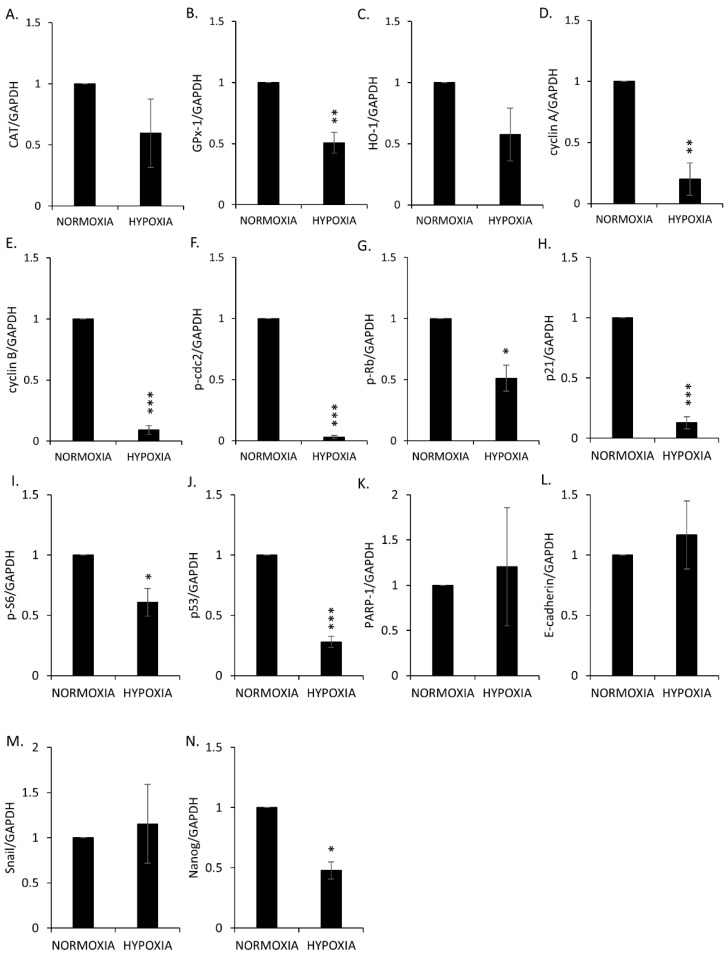
Evaluation of the protein expression in HCT116 cells treated with 2.5 μM IRINO in normoxia or hypoxia: antioxidative, proliferation, cell cycle inhibition, and EMT markers. Quantification is based on the densitometry analysis performed with the use of ImageJ software. Data are shown as the ratio of the protein amount to respective protein loading control (GAPDH): (**A**) CAT; (**B**) GPx-1; (**C**) HO-1; (**D**) cyclin A; (**E**) cyclin B; (**F**) p-cdc2; (**G**) p-Rb; (**H**) p21; (**I**) p-S6; (**J**) p53; (**K**) PARP-1; (**L**) E-cadherin; (**M**) Snail; (**N**) Nanog. Each bar represents mean ± SEM, n ≥ 3; * *p* < 0.05, ** *p* < 0.01, *** *p* < 0.001—normoxia vs. hypoxia.

**Figure A8:**
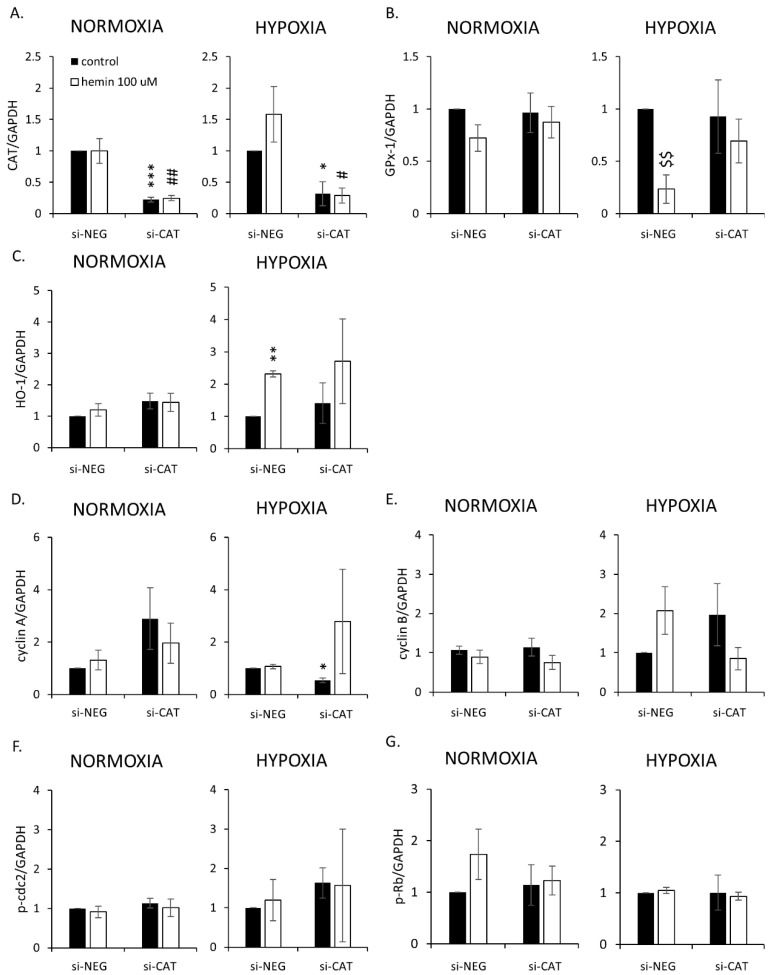
Evaluation of the protein expression in HCT116 cells, where gene encoding CAT was silenced with the use of siRNAs: antioxidative and proliferation markers. Cells were cultured in parallel in normoxia or hypoxia. Cells were treated with 2.5 μM IRINO and exposed to 100 μM hemin. Control cells were subjected to IRINO and NaOH. Quantification is based on the densitometry analysis performed with the use of ImageJ software. Data are shown as the ratio of the protein amount to respective protein loading control (GAPDH): (**A**) CAT; (**B**) GPx-1; (**C**) HO-1; (**D**) cyclin A; (**E**) cyclin B; (**F**) p-cdc2; (**G**) p-Rb. Each bar represents mean ± SEM, n ≥ 3; * *p* < 0.05, ** *p* < 0.01, *** *p* < 0.001—siNEG control vs. siCAT control; # *p* < 0.05, ## *p* < 0.01—siNEG hemin 100 μM vs. siCAT hemin 100 μM; $$ *p* < 0.01—control vs. hemin 100 μM.

**Figure A9:**
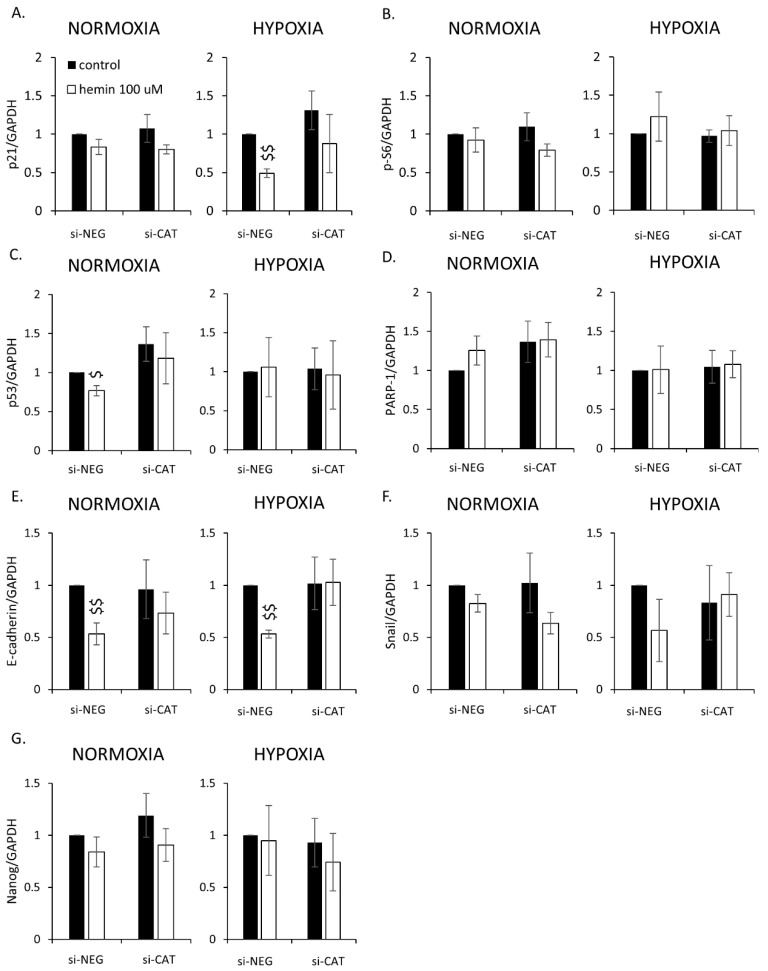
Evaluation of the protein expression in HCT116 cells, where gene encoding CAT was silenced with the use of siRNAs: cell cycle inhibition and EMT markers. Cells were cultured in parallel in normoxia or hypoxia. Cells were treated with 2.5 μM IRINO and exposed to 100 μM hemin. Control cells were subjected to IRINO and NaOH. Quantification is based on the densitometry analysis performed with the use of ImageJ software. Data are shown as the ratio of the protein amount to respective protein loading control l (GAPDH): (**A**) p21; (**B**) p-S6; (**C**) p53; (**D**) PARP-1; (**E**) E-cadherin; (**F**) Snail; (**G**) Nanog. Each bar represents mean ± SEM, n ≥ 3; $ *p* < 0.05, $$ *p* < 0.01—control vs. hemin 100 μM.

**Figure A10:**
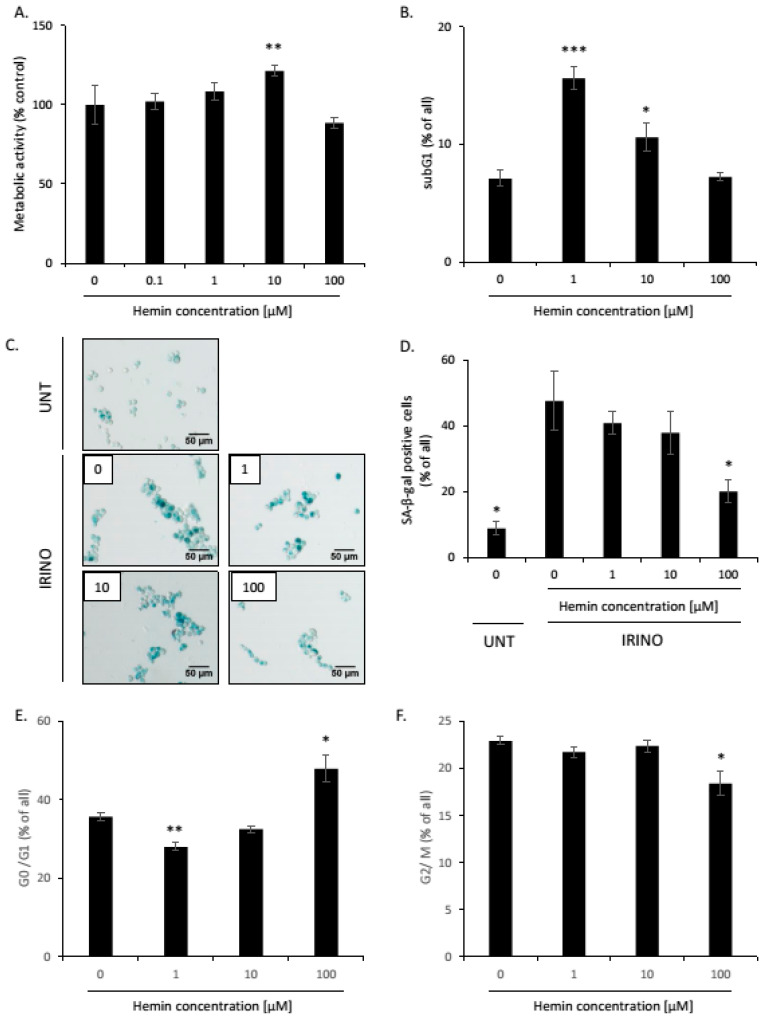
High hemin concentration correlates with an escape from the senescence of SW480 cells. SW480 cells were subjected to 2.5 μM of irinotecan (IRINO) and 1, 10, or 100 μM of hemin. After 24 h, the medium was changed and the cells were cultured in a drug-free medium for 4 days. (**A**) Evaluation of metabolic activity assayed by MTT metabolism. (**B**) Quantification of the percentage of cells in the subG1 phase (with DNA content < 2C). Cell cycle analysis was performed with the use of Cell Cycle Analysis Kit (Sigma) and flow cytometry. (**C**) Activity of SA-β-Galactosidase enzyme in UNT and IRINO-treated cells subjected to hemin. Detection of the enzyme was performed on cytospined cells. Representative photos were acquired using light microscopy. Original magnification 200×, scale bar 50 μM. (**D**) Quantification of SA-β-Gal positive cells after treatment with hemin. (**E**,**F**) Quantification of the percentage of cells in the G0/G1 (**E**) or G2/M phases (**F**). Cell cycle analysis was performed with Muse^TM^ Cell Cycle Reagent and flow cytometry. Each bar represents mean ± SEM, n ≥ 3. * *p* < 0.05, ** *p* < 0.01, *** *p* < 0.001—hemin concentration vs. 0.

**Figure A11:**
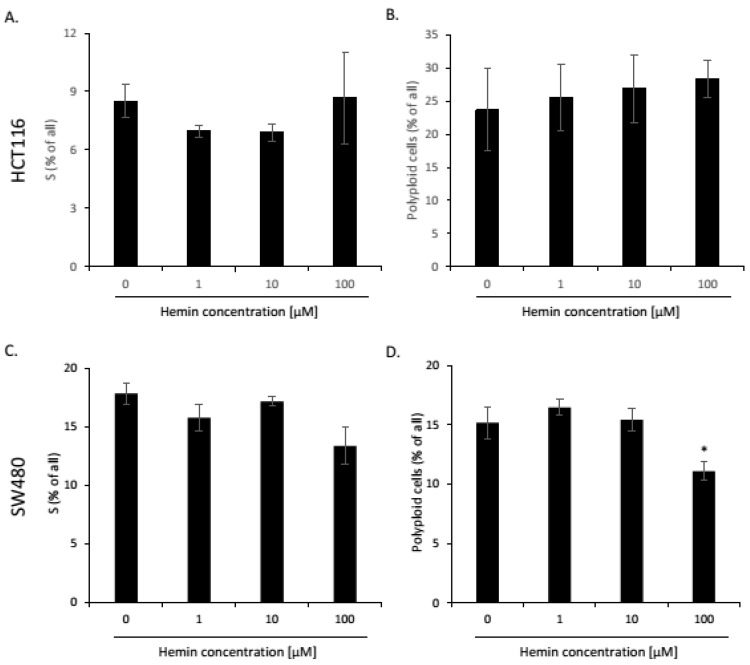
High hemin concentration slightly affects the distribution of HCT116 and SW480 cells in the S and polyploid phases of the cell cycle. HCT116 and SW480 cells were subjected to 2.5 μM of IRINO and 1, 10, or 100 μM of hemin. After 24 h, the medium was changed and the cells were cultured in a drug-free medium for 4 days. Quantification of the percentage of cells in the S phase in HCT116 (**A**) and SW480 cells (**C**). Quantification of the percentage of polyploid HCT116 (**B**) and SW480 cells (**D**). * *p* < 0.05.

**Figure A12:**
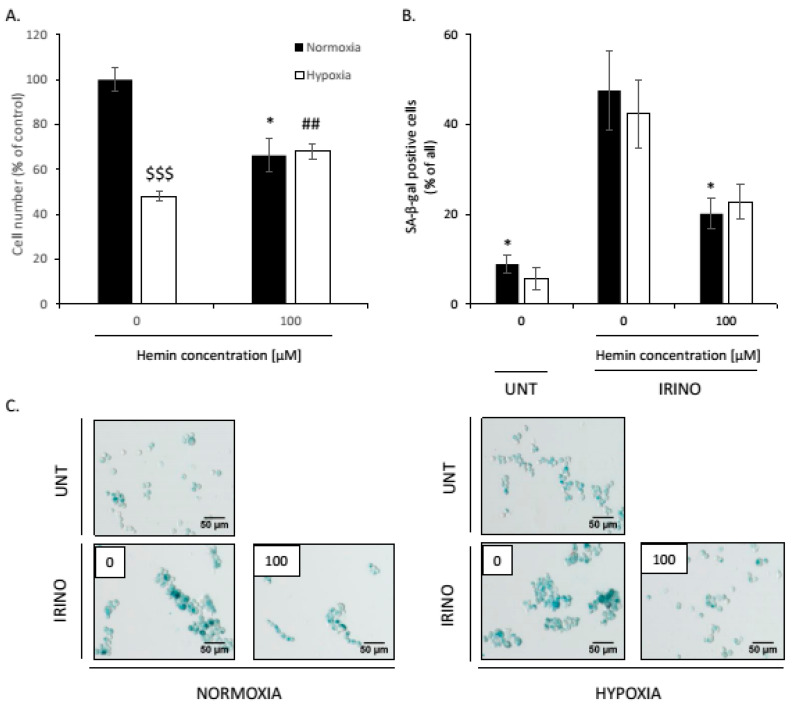
High hemin concentration correlates with an escape from senescence of hypoxic SW480 cells: cell number and activity of SA-β-Gal. Cells were cultured in parallel in normoxia or hypoxia. Cells were exposed to 100 μM hemin. Control cells were subjected to IRINO and NaOH in the corresponding dose. (**A**) Evaluation of cell number. Cell number was counted using Bürker’s chamber. (**B**) Activity of SA-β-Gal enzyme in IRINO-treated cells subjected to hemin under normoxic or hypoxic conditions. Detection of the enzyme was performed on cytospined cells. (**C**) Quantification of SA-β-Gal positive cells after treatment with hemin. Representative photos were acquired using light microscopy. Original magnification 200 x, scale bar 50 μM; Each bar represents mean ± SEM, n ≥ 3; * *p* < 0.05—vs. control normoxia; ## *p* < 0.01—vs. control hypoxia; $$$ *p* < 0.001—normoxia vs. hypoxia.

**Figure A13:**
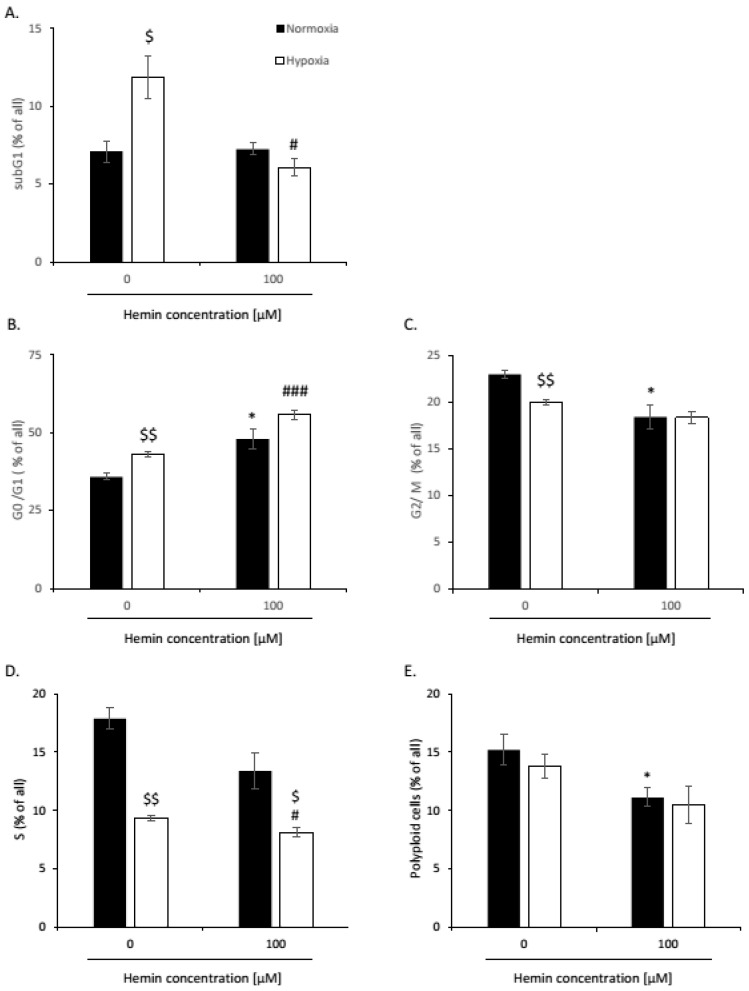
High hemin concentration correlates with an escape from senescence of hypoxic SW480 cells: changed accumulation in different phases of the cell cycle. Quantification of the percentage of cells in the subG1 (**A**), G0/G1 (**B**), G2/M (**C**), and S phases (**D**), and polyploid cells (**E**). Cell cycle analysis was performed with the Cell Cycle Analysis Kit (Sigma) and flow cytometry; Each bar represents mean ± SEM, n ≥ 3; * *p* < 0.05—vs. control normoxia; # *p* < 0.05, ### *p* < 0.001—vs. control hypoxia; $ *p* < 0.05, $$ *p* < 0.01—normoxia vs. hypoxia.

Hypoxia reduced the expression of proteins related to cell proliferation: cyclin A, cyclin B, p-cdc2, and p-Rb (Figure 9B and Figure A7D–G); cell cycle inhibition/senescence: p21, p-S6, and p53 (Figure 9C and Figure A7H–J); and stemness: Nanog (Figure 9D and Figure A7N). It did not change the expression of DNA repair protein, PARP-1 (Figure 9C and Figure A7K), and EMT/MET proteins: E-cadherin and Snail (Figure 9D and Figure A7L,M). In terms of antioxidative enzymes, hypoxia significantly downregulated the expression of GPx-1 (Figure 9A and Figure A7B) and showed a tendency to decrease the levels of CAT (Figure 9A and Figure A7A) and HO-1 (Figure 9A and Figure A7C). Hemin more strongly induced HO-1 in hypoxia than in normoxia, regardless of CAT status (Figure 9A and Figure A8C). In contrast, it did not affect CAT and GPx-1 expression in hypoxia differently than in normoxia (Figure 9A and Figure A8A,B). In terms of proliferation markers, only two proteins—cyclin A and cyclin B—were affected, and their expression seemed to be balanced by hypoxia, hemin, and CAT expression (Figure 9B and Figure A8D,E). The expression of cyclin A was upregulated in CAT-silenced cells in normoxia, whereas in hypoxia, the effect was the opposite (Figure 9B and Figure A8D). In contrast, the expression of cyclin B was elevated in CAT-silenced cells, but only in hypoxia. Hemin seemed to compensate for these effects (Figure 9B and Figure A8E). In this vein, hemin downregulated the expression of cell cycle inhibitor p21 regardless of oxygen tension and CAT expression (Figure 9C and Figure A9A). A similar effect was observed for another cell cycle inhibitor, p53, but only in normoxia and in the presence of CAT (Figure 9C and Figure A9C). Hemin also reduced the epithelial marker expression for E-cadherin both in normoxia and hypoxia. The observed effects were reversed in the absence of CAT (Figure 9D and Figure A9E). There were no changes in the expression of p-cdc2 (Figure 9B and Figure A8F), p-Rb (Figure 9B and Figure A8G), p-S6 (Figure 9C and Figure A9B), PARP-1 (Figure 9C and Figure A9D), Snail (Figure 9D and Figure A9F), and Nanog (Figure 9D and Figure A9G).

Altogether, these data showed that hypoxia reduced the expression of proteins related to proliferation, cell cycle inhibition, and antioxidative defense, whereas those related to EMT/MET and DNA repair were not changed. Upon hemin addition, there was HO-1 and cyclins activation, followed by p21 reduction and EMT. CAT expression maintained a reduced expression of E-cadherin and further upregulated cyclins’ expression.

## 3. Discussion

Cellular senescence has been considered for a long time as an effect synergistic with cancer prevention and therapy [40,41,42,43,44,45,46]. However, recent data show that TIS may lead to cancer recurrence through amitotic divisions and/or SASP-dependent effects on the tumor microenvironment [11,22,23,25,26,27,35,36,39,47,48,49,50,51,52,53,54]. Therefore, we postulate that TIS cells should be considered a double-edged sword and/or Trojan horse in cancer development/acquiring resistance to therapies [16,17]. The questions of what triggers the unusual divisions of senescent cancer cells and how we can control them remain unanswered.

Hemoglobin from either red meat or bowel bleeding may serve as an enhancer of oxidative damage in the bowel and increase the risk of colorectal cancer [4,5]. However, molecular mechanisms beyond that phenomenon remain unclear. Therefore, here, we asked the question of whether the oxidative stress induced by hemin can play a role in the escape of cancer cells from senescence. The most important findings of our study are the following: (1) high concentration of prooxidants, hemin, and H_2_O_2_ can trigger escapes from senescence of colon cancer cells, which is manifested by the appearance of a highly proliferating progeny and a shift in EMT/MET markers; (2) silencing of antioxidative enzymes, CAT, GPx-1, and HO-1 maintain senescent cells in the non-proliferating state; (3) in contrast to CAT and GPx-1, the expression of HO-1 is reduced upon the induction of senescence but is restored when cancer cells escape from it; and (4) hemin enhances the escape from senescence in hypoxic cells through HO-1/cyclins activation.

In our previous studies, we demonstrated that human colon cancer cells treated with chemotherapeutic agents—doxorubicin, irinotecan, and 5-fluorouracil—underwent senescence in long-term cell cultures, but after withdrawal of the drug, cell re-growth was observed [25,26,27,36]. Moreover, a subpopulation of TIS cells exhibited certain features of cancer stem cells, namely, elevated Nanog expression, augmented proportion of CD24^+^ cells and side population, epithelial-to-mesenchymal transition (EMT), and tumor formation in NOD/SCID animals [26,27]. Additionally, in another study, we showed that doxorubicin-treated HCT116 cells displayed an increased production of reactive oxygen species (ROS), whereas a decreased level of ROS prevented the cells from escaping senescence [25]. Therefore, here, we asked the question of whether antioxidative systems can play a role in cancer cells escaping from senescence. In the present study, we showed that the induction of senescence in colon cancer cells led to the upregulation of two enzymes involved in H_2_O_2_ utilization, catalase, and glutathione peroxidase-1 but the downregulation of heme oxygenase-1, heme degrading enzyme. To check whether we would be able to reverse this effect, we treated HCT116 and SW480 cells with hemin, a major substrate and an activator of HO-1. In accordance, we demonstrated that at a high concentration (100 μM) hemin led to HO-1 induction and escape from senescence. It correlated with the appearance of SA-β-galactosidase negative cells, progression through the G0/G1 phase, and upregulation of proliferation and EMT markers. Similar effects were obtained when senescent cells were treated with another prooxidant, H_2_O_2._ The overexpression of HO-1 has been demonstrated in many cancers [55,56,57] and its level could be further increased in response to oxidative stress, chemo-, radio- [58], or photodynamic therapy [59,60,61]. Moreover, we showed that HO-1 may exert potent and complex effects depending on the cancer type and microenvironmental context [59,62,63,64,65,66,67,68,69,70]. However, in the perspective of senescence and escaping from senescence of cancer cells, the role of HO-1 has not been studied yet. Interestingly, in a model of myeloid cells, Hedblom and coworkers demonstrated that the deletion of HO-1 resulted in an impaired DNA damage response (DDR), reduced cell proliferation, and increased cellular senescence [71]. Additionally, there are few reports of solid cancers or their metastases presenting with localized bruising. Bruising has been described in parathyroid adenomas [72], orbital neuroblastoma [73], neurofibromatosis [74], and malignant melanoma [75,76]. These data may suggest that the heme/HO-1 system may participate in tumor initiation and metastases. In accordance, the role of HO-1 in cell dedifferentiation/stemness has been shown in several models [66]. For example, Kim and coworkers demonstrated that HO-1-derived CO production participated in the manifestation of breast cancer stem cell-like properties and stimulated the formation of mammospheres through activation of Notch-1 signaling [77]. In our recent paper, we showed that HO-1 had effects on melanoma stem cells’ properties [69]. We and others also showed that TIS cells may exhibit certain features of cancer-initiating cells in the dormant state [22,26,27,50,54,78,79]. Polyploid, giant, cancer cells (PGCCs) containing an elevated genomic content and resembling blastocyst might be key players in the evolution of cancer and long-term resistance to therapies [27,34,35]. Additionally, we demonstrated that hypoxia increased the escape from senescence of lung and colon cancer cells treated with chemotherapeutic drugs [39]. In this vein, Zhang and coworkers reported that PGCCs, which possess features of cancer-initiating cells, could be induced from ovarian cancer cells by the hypoxia-mimetic agent, CoCl_2_ [54]. They showed that PGCCs from ovarian cancer could grow into tumor spheroids, initiate tumor growth in nude mice, and differentiate into other benign cell types in vitro and in vivo [54,79,80,81]. These properties of PGCCs were also reported in colon cancer [82]. Interestingly, Li and coworkers demonstrated that PGCCs also produced embryonic hemoglobin-delta and -zeta with a strong oxygen-binding ability and erythroid differentiation-related proteins, promoting the survival of tumor cells in a hypoxic microenvironment [83]. Here, we showed that hemin enhances escape from senescence in hypoxic colon cancer cells through HO-1/cyclins activation. In line with our data, exposure to hemin increased the gene and protein expression level of HO-1 and diminished the ROS levels, senescence, and the inflammatory profile in fibroblasts isolated from the lung biopsies of patients with chronic obstructive pulmonary disease. It also rescued mitochondria dysfunction by restoring mitophagy [84]. Moreover, HO-1 improved heart function and attenuated cardiomyocyte senescence triggered by ischemic injury and aging [85], whereas hemin enhanced the cardioprotective effects of mesenchymal stem cell-derived exosomes against infarction via amelioration of cardiomyocyte senescence [86]. Interestingly, Luo and coworkers showed that HO-1 was accumulated in the nuclei of stress-induced senescent endothelial cells and conferred protection against endothelial senescence independent of its enzymatic activity [87]. Altogether, our data suggest that the hemin/HO-1 system may participate in escaping from the senescence of cancer cells. Therefore, we propose that it could be used in targeted anti-cancer therapy.

In this study, we also showed that, similarly to hemin, another prooxidant H_2_O_2_ leads to an escape from senescence. Hydrogen peroxide is freely diffusible and is a relatively long-lived molecule. Although it is not very reactive, it is the precursor of many other reactive oxygen species (ROS). One of the most important products is a strongly reactive hydroxyl radical (⋅OH). The reaction takes place in the presence of transition metal ions such as Fe^2+^ using the Fenton reaction. Among the by-products of heme degradation by HO-1 are iron ions. H_2_O_2_, ⋅OH, and other ROS are essential for various biological processes, e.g., proliferation or differentiation in normal and cancer cells [88]. ROS has been shown to play tumor-promoting but also tumor-suppressing actions [89]. Importantly, ROS production is a mechanism shared by most chemotherapeutics [90]. Recent evidence suggests that prolonged chemotherapy can reduce the overall cellular ROS in cancer, which is believed to function as a key underlying mechanism of drug resistance in chemotherapy [91]. SIPS might be induced by various stressful agents, including ROS or chemotherapeutics. Some data suggest that ROS may play a role not only in the induction of senescence but also in its escape. Gosselin and collaborators reported that oxidative stress induced epithelial cell senescence and that such cells were able to divide by budding [92]. Similarly, Achuthan and coworkers demonstrated that a senescent population of cancer cells had a high level of ROS, whereas their progeny displayed stem-like cell features but low ROS production [93]. In accordance, our previous study showed that the ROS scavenger, Trolox, protected from the emergence of escapers [25]. Recently, Dharmalingam and coworkers showed that, while free iron produced mostly reactive oxygen species (ROS)-related single-strand DNA breaks, hemin induced rapid and persistent nuclear and mitochondrial double-strand breaks (DSBs) in neuronal and endothelial cell genomes and mouse brains following experimental intracerebral hemorrhage. These were comparable to that seen with γ-radiation and DNA-complexing chemotherapies. While they applied antioxidant therapy to prevent senescence, cells became again sensitized to ferroptosis [94]. Here, we demonstrated that, upon the induction of senescence with IRINO, the expression of CAT and GPx-1, enzymes that utilize H_2_O_2_, was strongly upregulated in colon cancer cells. Moreover, when cells were treated with high hemin concentration, which led to escaping from senescence, the CAT and GPx-1 levels were further elevated. In turn, when senescent cells were treated with H_2_O_2_, the CAT expression was maintained at a high level during the whole experiment (7 days), dropping down by the end of it. It correlated with the strong increase in proteins related to cell cycle progression—cyclin A, cyclin B, p-cdc2, and p-Rb—and the appearance of a small, highly proliferating progeny. Interestingly, when we silenced CAT and GPx-1 in senescent colon cancer cells with specific siRNAs and treated these cells with hemin, we observed that the expressions of cyclins, especially cyclin B, were reduced. As cyclin B is necessary for the progression of the cells into and out of the M phase of the cell cycle, we propose that excessive ROS production, unbalanced by activities of antioxidative enzymes CAT and GPx-1, inhibits escaping from senescence. The intrinsic levels of antioxidant enzymes are low in a majority of cancer cell types as compared to non-transformed cells. This suggests that those cancer cells may lack the biochemical machinery, which is crucial for detoxifying higher fluxes of H_2_O_2_. Kinetic models built using in vitro data have shown that CAT is the major enzyme responsible for the detoxification of high concentrations of H_2_O_2_, whereas GPx-1 is involved in removing low fluxes of H_2_O_2_ [95]. Our data suggest that upregulation of CAT and GPx-1 in senescent cancer cells may be a characteristic feature and may help distinguish them from non-senescent cancer cells. Additionally, we propose that this phenomenon could be responsible for the enhanced resistance of senescent cancer cells to oxidative stress and oxidative stress-based anticancer therapies.

Taken together, this work shows that high hemin or H_2_O_2_ concentrations could be a trigger for colon cancer cells to escape from senescence. However, the outcome depends on the proper activities of antioxidative enzymes: HO-1, CAT, and GPx-1. HO-1 expression, which is reduced upon induction of senescence, seems to be restored when cancer cells escape from it. Oppositely, the CAT and GPx-1 levels are strongly elevated in cancer cells that undergo TIS. Hemin enhances escaping from senescence in hypoxic colon cancer cells through HO-1/cyclins activation. Therefore, our data indicate that the oxidative status of senescent cancer cells differs from their non-senescent counterparts and we suggest its use in senolytic-oriented therapy. A thorough combination of prooxidants and/or antioxidative enzymes’ activity could be a valuable help to prevent senescent cells from re-activating their proliferative properties and cancer re-growth after chemotherapies.

## 4. Materials and Methods

### 4.1. Chemicals and Antibodies

Unless otherwise specified, chemicals and reagents were purchased from Sigma Aldrich (Taufkirchen, Germany). A list of the antibodies is presented in the table below (Table 1):

### 4.2. Cells and Treatment

Human colon HCT116 cancer cells were kindly provided by Dr. Bert Vogelstein (Johns Hopkins University, Baltimore, MD, USA). Authentication of the HCT116 cell line was performed with Cell Line Authentication IdentiCell STR. Human colon cancer cell line SW480 was purchased from ATCC. Cells were grown under standard normoxic conditions (~19% pO_2_, 37 °C, 5% CO_2_) or in hypoxia (1% pO_2_, 37 °C, 5% CO_2_) in McCoy’s medium supplemented with 10% fetal bovine serum, 10,000 units/mL of penicillin, 10,000 µg/mL of streptomycin, and 25 µg/mL of amphotericin B (Antibiotic-Antimycotic).

To induce senescence, cells were seeded at a density of 250 000/per 25 cm^2^ flask 24 h before treatment. Colon cancer cells were cultured in the presence of 2.5 μM (HCT116) or 2.5 μM (SW480) irinotecan (IRINO) for 24 h followed by 4–7 days in a fresh medium without a drug. In the experiments, where growing time lasted for 7 days after the incubation with the drugs, the medium was changed to the fresh one on day 4. Doses of IRINO used in experiments were chosen as most efficient to induce senescence but not cell death, based on staining for SA-β-gal activity, cell counting in Bürker’s chamber, BrdU incorporation assay, and cell cycle analysis with PI staining [27]. Additionally, cells were incubated with hemin or H_2_O_2_ during the chemotherapeutic treatment. Incubation with drugs lasted 24 h; after that time, the medium was changed to drug-free one and the cells were grown for additional 4 or 7 days. NaOH was used as a solvent for hemin. Cells treated with NaOH were used as a control in the hemin-based experiments. The experiments were performed in technical duplicates or triplicates. All tests were performed in at least three biological repetitions.

### 4.3. Experiments in Hypoxia

In the experiments performed on 96-well plates, cells were seeded at 1500 cells/per well for HCT116 or SW480 cells. For the remaining tests, 125,000 cells were plated on a 25 cm^2^ flask for each cell line. After seeding, cells were grown in normoxia for 24 h. Half of the plates/flasks were then left in normoxic condition, whereas the remaining culture dishes were transferred to the hypoxic chamber, where the medium was changed to the hypoxic one. The hypoxic medium was incubated for at least 24 h in the hypoxic chamber before the experimental procedure. After 24 h of culture in normoxia or hypoxia, cells were treated with chemotherapeutics for the next 24 h. After 24 h of incubation with chemotherapeutic drugs, the medium was changed and the cells were cultured in a drug-free medium for the next 7 days. Additionally, the medium was changed on day 4.

### 4.4. Gene Silencing

HCT116 cells were seeded at 750 per well or 62,500 cells per 25 cm^2^ flask in normoxic conditions. Twenty-four hours later, half of the plates/flasks were left in normoxic condition, whereas the remaining culture dishes were transferred to the hypoxic chamber and the medium was changed. After a 24 h incubation, the cells were transfected with pooled siRNAs targeting: HO-1, GPx-1, or CAT (ON-TARGETplus siRNA, Dharmacon, Lafayette, CO, USA) using lipofectamine RNAiMAX (Invitrogen, Life Technologies Corporation, Carlsbad, CA, USA). Nontargeting siRNAs were used as a control. Lipofectamine and siRNA dilutions were prepared according to manufacturers’ protocols. The next day, cancer cells were treated with respective chemotherapeutics for 24 h followed by 4 days of drug-free culture. Afterward, the effects of gene silencing on protein expression, cell number, and SA-β-gal activity were analyzed.

### 4.5. Cell Viability Assay by MTT Metabolism

Cell viability was determined by measuring the conversion of MTT (3-(4,5-dimethylthiazol-2-yl)-2,5-diphenyltetrazolium bromide, the final concentration of 0.5 mg/mL) to formazan in living cells. HCT116 and SW480 colon cancer cells were cultured according to the description provided in Section 4.3 and Section 4.4. After 2 h of incubation with MTT at 37 °C, formazan crystals were dissolved in a lysis buffer containing 50 mM HCl in isopropanol. Optical density was measured at 562 nm using a scanning multi-well spectrophotometer.

### 4.6. Western Blotting

Cells were harvested and put into RIPA lysis buffer and frozen overnight. After defrosting, the cell suspension was centrifuged at 10,000× *g*. The concentration of proteins was estimated by the BCA method. Laemmli SDS sample buffer was added to lysates before separation by the SDS-PAGE method. The same protein amount was loaded into each well. The semi-dry transfer was used (20 V for 20 min). After the transfer membranes were stained with Ponceau S and cut according to the protein size ladder. After that, membranes were blocked in 5% non-fat milk and probed overnight at 4 °C with antibodies specific (1:1000) for HO-1, CAT, GPx1, p21, p-S6, p-p53, p53, E-cadherin, Snail, Nanog, cyclin A, cyclin B, p-Rb, p-cdc2, and PARP-1. GAPDH (1:50,000) or Vinculin (1:1000) was used as a loading control. For the process of the incubation, antibodies were diluted in TBS buffer supplemented with: 5% Bovine Serum Albumin or 5% non-fat milk, 0.05% Tween, and 0.01% sodium azide. Then, proteins were detected using appropriate secondary HRP-conjugated antibodies (1:10,000), diluted in TBS buffer supplemented with 5% non-fat milk and 0.05% Tween, and ECL reagents as recommended by the manufacturer. The uncropped blots are shown in the Supplementary Materials.

### 4.7. Detection of Senescence-Associated β-Galactosidase (SA-β-Gal)

The activity of SA-β-gal was assessed according to modified Dimri et al. [96]. Cells were trypsinized and fixed with 2% formaldehyde and 0.2% glutaraldehyde diluted in PBS. After that, cells were washed with PBS, cytospined on microscopic slides, and incubated overnight at 37 °C with a solution containing 1 mg/mL 5-bromo-4-chloro-3-indolyl-b-D-galactopyranoside, 5 mM potassium ferrocyanide, 150 mM NaCl, 2 mM MgCl_2_, and 0.1 M phosphate buffer, pH 6.0. All the reagents were brought to room temperature before they were mixed. Microscopic slides were sealed with the use of a Dako medium.

### 4.8. DNA Content Analysis

Cells were harvested, centrifuged at 1200× *g* for 5 min, suspended in 500 μL of PBS, and fixed in 5 mL of 70% frozen EtOH. Fixed cells were stored at −20 °C for at least 24 h. Before analysis, cells were centrifuged to remove EtOH. Then, cells were washed with PBS, centrifuged, and suspended in 250 μL of Muse^TM^ Cell Cycle Reagent or Cell Cycle Analysis Kit (Sigma). DNA content analyses were performed using a Becton-Dickinson FACS Calibur and the BD CellQuest Pro 6.0 software or CytoFlex Beckman Coulter and CytExpert software. Cells were left in the dark for 30 min during the process of staining. Data for 50,000 cells were collected and analyzed.

### 4.9. Statistical Analysis

All experiments were repeated at least three times. The experiments to determine cell viability with the use of MTT assay were performed in three technical repetitions. The numerical results are expressed with mean values ± standard errors. *p*-values were assessed using the type-2 two-tailed *t* (Student) test. *p* < 0.05 was considered statistically significant.

## 5. Conclusions

In the current study, we show that oxidative stress induced by hemin or H_2_O_2_ could be a trigger for escaping from the senescence of colon cancer cells. However, the outcome depends on the proper activities of antioxidative enzymes: HO-1, CAT, and GPx-1. Therefore, a proper combination of prooxidants and/or antioxidative enzymes’ activity could prevent senescent cells from re-activating their proliferative properties and cancer re-growth after chemotherapies.

## Figures and Tables

**Table 1:** List of the antibodies.

Name of the Antibody	Clonality	Clone	Phosphorylation Site	Host	Manufacturer	Reference Number
Catalase	Polyclonal	-	-	Rabbit	Abcam	ab16731
Cyclin A	Monoclonal	BF683	-	Mouse	Santa Cruz Biotechnology	sc-239
Cyclin B	Monoclonal	D5C10	-	Rabbit	Cell Signalling	12231
E-cadherin	Monoclonal	24E10	-	Rabbit	Cell Signalling	3195
GAPDH	Monoclonal	6C5	-	Mouse	Millipore	MAB374
GPx-1	Polyclonal	-	-	Rabbit	Abcam	ab22604
HO-1	Polyclonal		-	Rabbit	Enzo Life Sciences	ADI-SPA-894
Nanog	Monoclonal	D73G4	-	Rabbit	Cell Signalling	4903
p21	Monoclonal	12D1	-	Rabbit	Cell Signalling	2947
p21	Monoclonal	CP74		Mouse	Sigma-Aldrich	P1484
p53	Monoclonal	DO-1	-	Mouse	Santa Cruz Biotechnology	sc-126
PARP-1	Monoclonal	C2-10	-	Mouse	BD-Biosciences	556362
p-cdc2	Monoclonal	10A11	Tyr15	Rabbit	Cell Signalling	4539
p-p53	Monoclonal	16G8	Ser15	Mouse	Cell Signalling	9286
p-Rb	Polyclonal	-	Ser807/811	Rabbit	Cell Signalling	9308
p-S6	Monoclonal	D57.2.2E	Ser235/236	Rabbit	Cell Signalling	4858
Snail	Monoclonal	C15D3	-	Rabbit	Cell Signalling	3879
Vinculin	Monoclonal	V284	-	Mouse	Sanata Cruz Biotechnology	sc-59803
γ-H2AX	Monoclonal	9F3	Ser139	Mouse	Abcam	ab26350

## Data Availability

The datasets generated during and analyzed during this study are available from the corresponding author upon reasonable request.

## Supplementary Materials

The following are available online at https://www.mdpi.com/article/10.3390/cancers14194793/s1, Full pictures of the Western blots.
