# Supplementary material for: High Hemin Concentration Induces Escape from Senescence of Normoxic and Hypoxic Colon Cancer Cells
Source: Cancers (Basel). 2022 Sep 30;14(19):4793. doi: 10.3390/cancers14194793 (PMC9564005; doi:10.3390/cancers14194793)
Supplement: Supplementary file 1 [file cancers-14-04793-s001.zip › cancers-1842606-supplementary.pdf]

Representative blots showing raw data of protein expression analysis: (**1–11**) in HCT116 cells treated with 2.5 μM IRINO and exposed to 1, 10 or 100 μM hemin: (1) HO-1; (2) CAT; (3) GPx-1; (4) cyclin A; (5) cyclin B; (6) p-cdc2; (7) p-Rb; (8) p21; (9) p-S6; (10) PARP-1; (11) γ-H2AX; (**12–24**) in HCT116 treated with 2.5 μM IRINO and exposed to 5 or 10 μM H2O2 and collected after 4th and 7th days of the experiment: (12) HO-1; (13) CAT; (14) GPx-1; (15) cyclin A; (16) cyclin B; (17) p-cdc2; (18) p-Rb; (19) p21; (20) p-S6; (21) p53; (22) E-cadherin; (23) Snail; (24) Nanog; (**25–37**) in HCT116 cells treated with 2.5 μM IRINO and exposed to 100 μM hemin, where antioxidative enzymes: HO-1, CAT or GPx-1 were silenced with use of siRNAs: (25) HO-1; (26) CAT; (27) GPx-1; (28) cyclin A; (29) cyclin B; (30) p-cdc2; (31) pRb; (32) p21; (33) p-S6; (34) PARP-1; (35) E-cadherin; (36) Snail; (37) Nanog; (**38–51**) in HCT116 cells treated with 2.5 μM IRINO and exposed to 100 μM hemin, where CAT was silenced with use of siRNAs, cells were cultured in normoxia or hypoxia: (38) HO-1; (39) CAT; (40) GPx-1; (41) cyclin A; (42) cyclin B; (43) p-cdc2; (44) p-Rb; (45) p21; (46) p-S6; (47) PARP-1; (48) p53; (49) E-cadherin; (50) Snail; (51) Nanog.

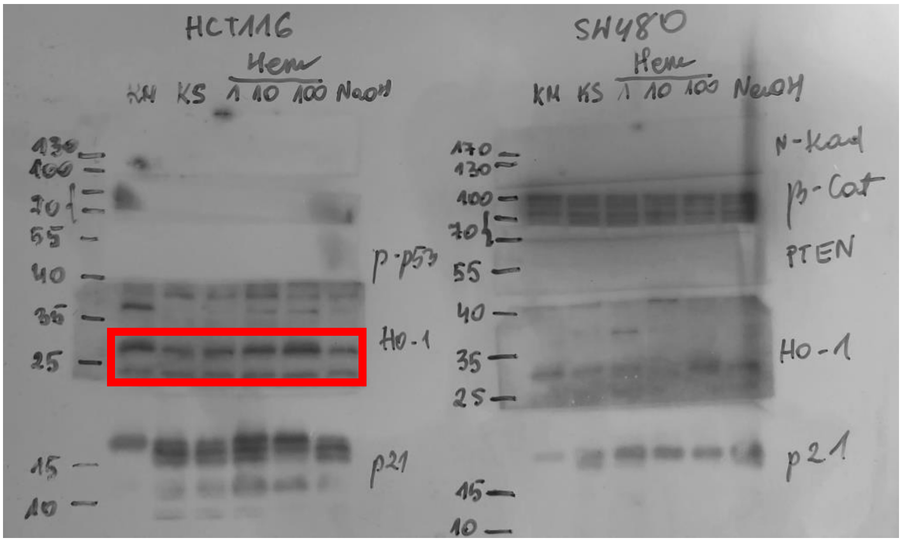

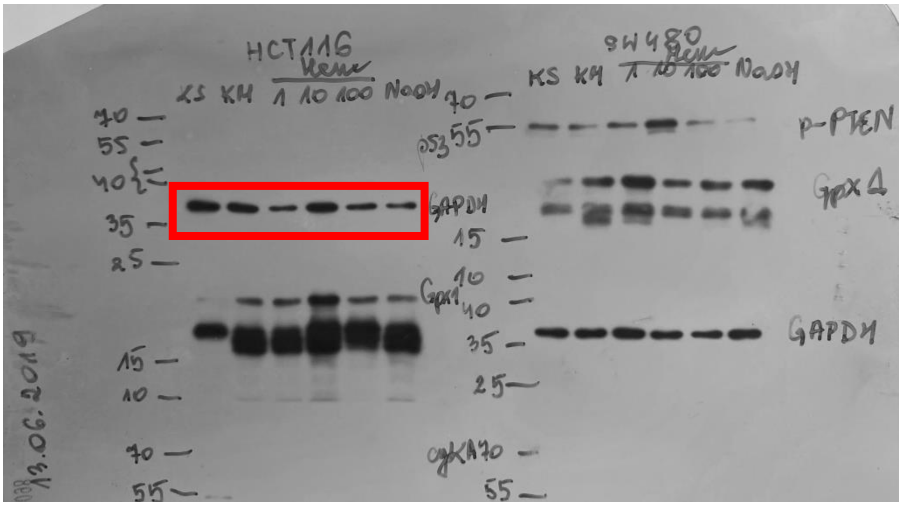

KM – Untreated Control
KS – Irinotecan
1 – Irinotecan + 1 uM Hemin
10 – Irinotecan + 10 uM Hemin
100 – Irinotecan + 100 uM Hemin
NaOH – Irinotecan + NaOH

Fig. A.II.1

CAT **HCT116**

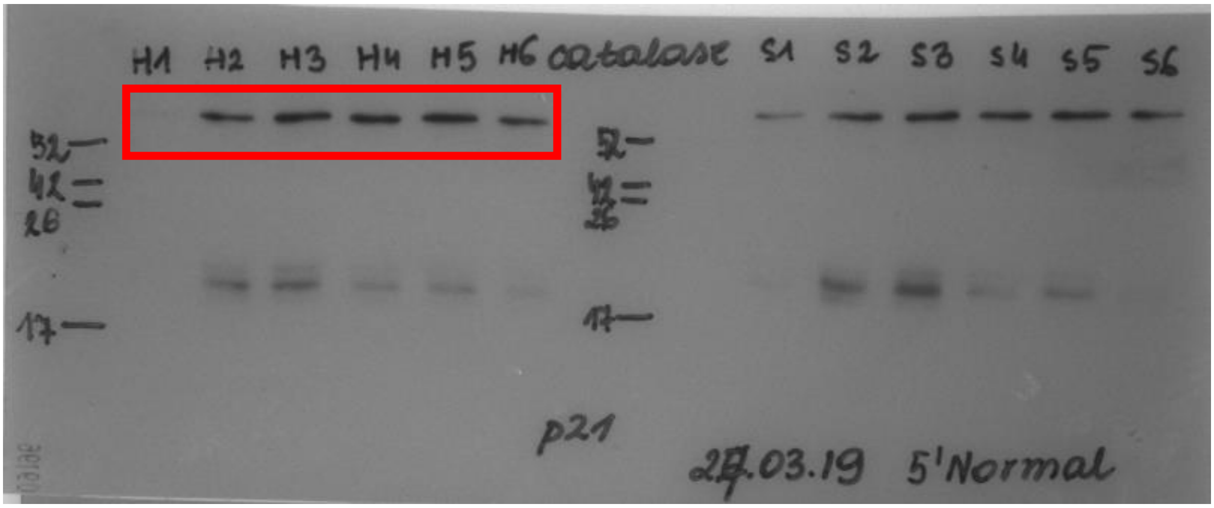

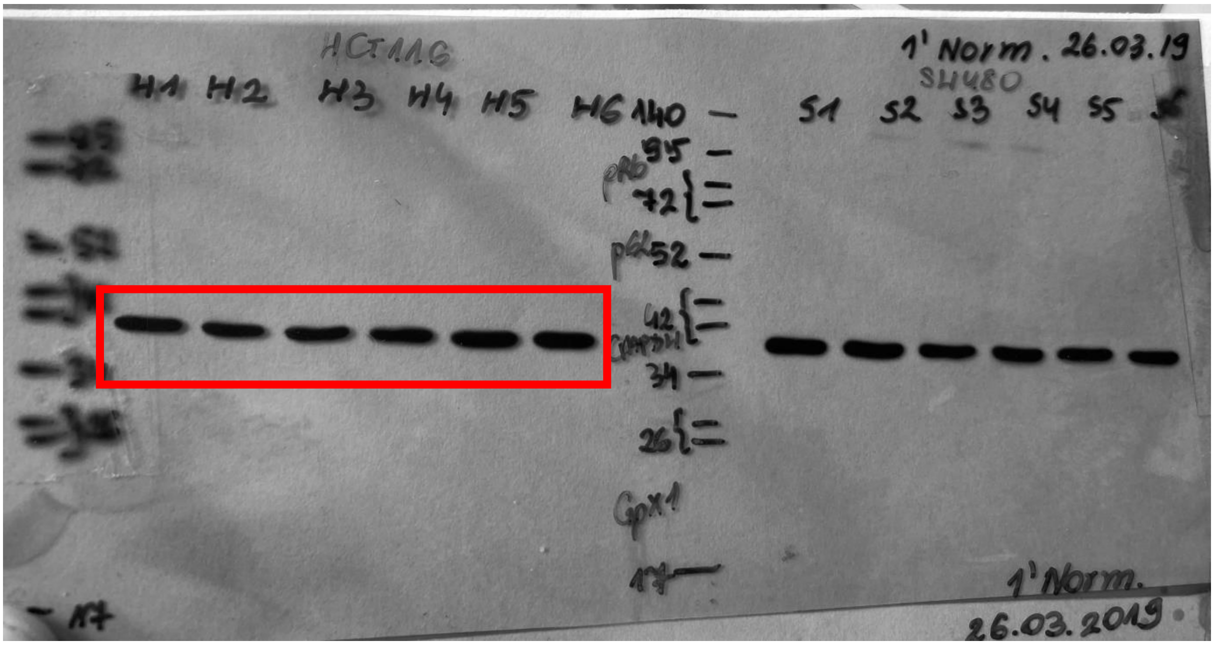

H1 – Untreated Control
H2 – Irinotecan
H3 – Irinotecan + 1 uM Hemin
H4 – Irinotecan + 10 uM Hemin
H5 – Irinotecan + 100 uM Hemin
H6 – Irinotecan + NaOH

Fig. A.II.2

GPx-1

HCT116

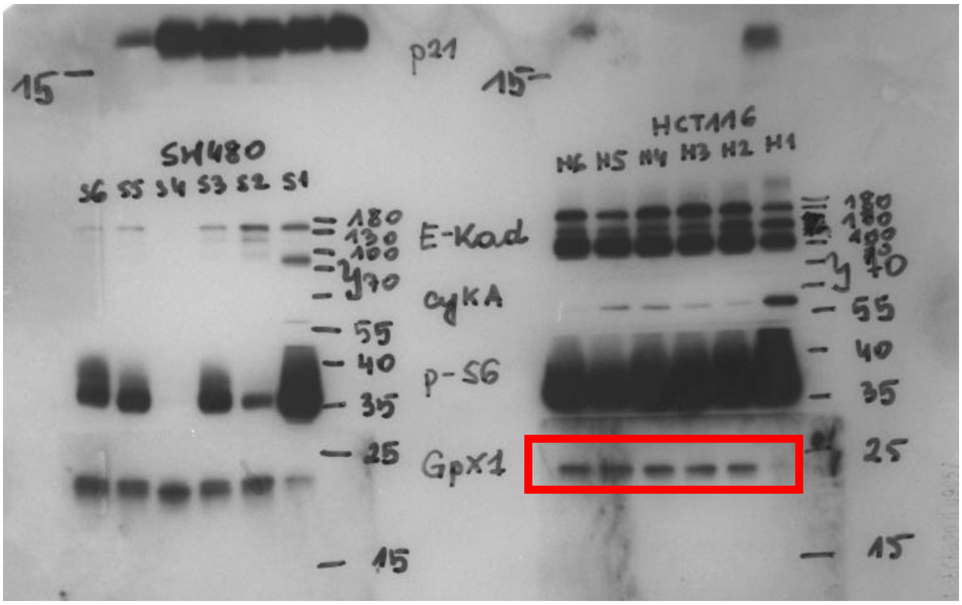

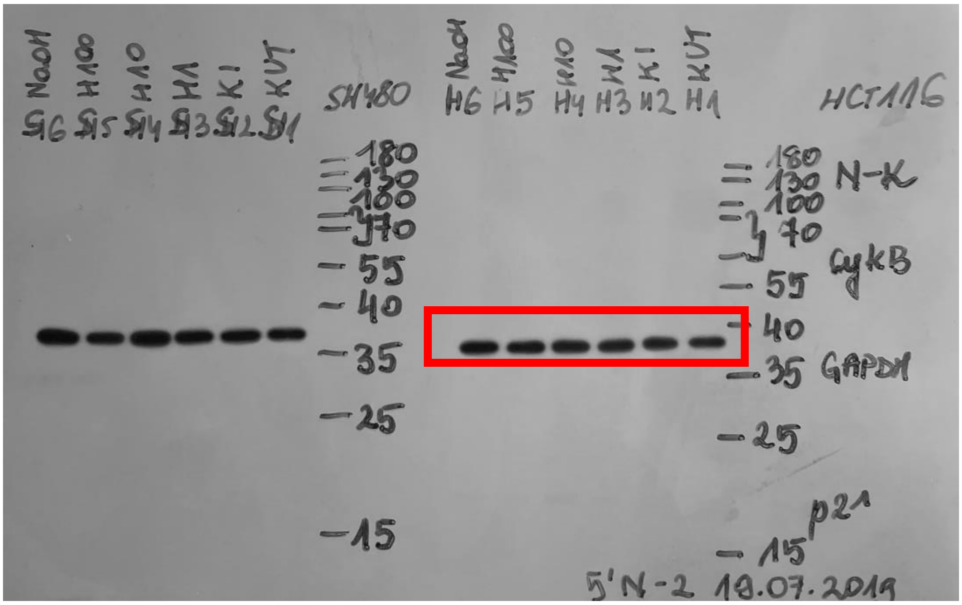

H1 – Untreated Control
H2 – Irinotecan
H3 – Irinotecan + 1 uM Hemin
H4 – Irinotecan + 10 uM Hemin
H5 – Irinotecan + 100 uM Hemin
H6 – Irinotecan + NaOH

Fig. A.II.3

cyclin A

HCT116

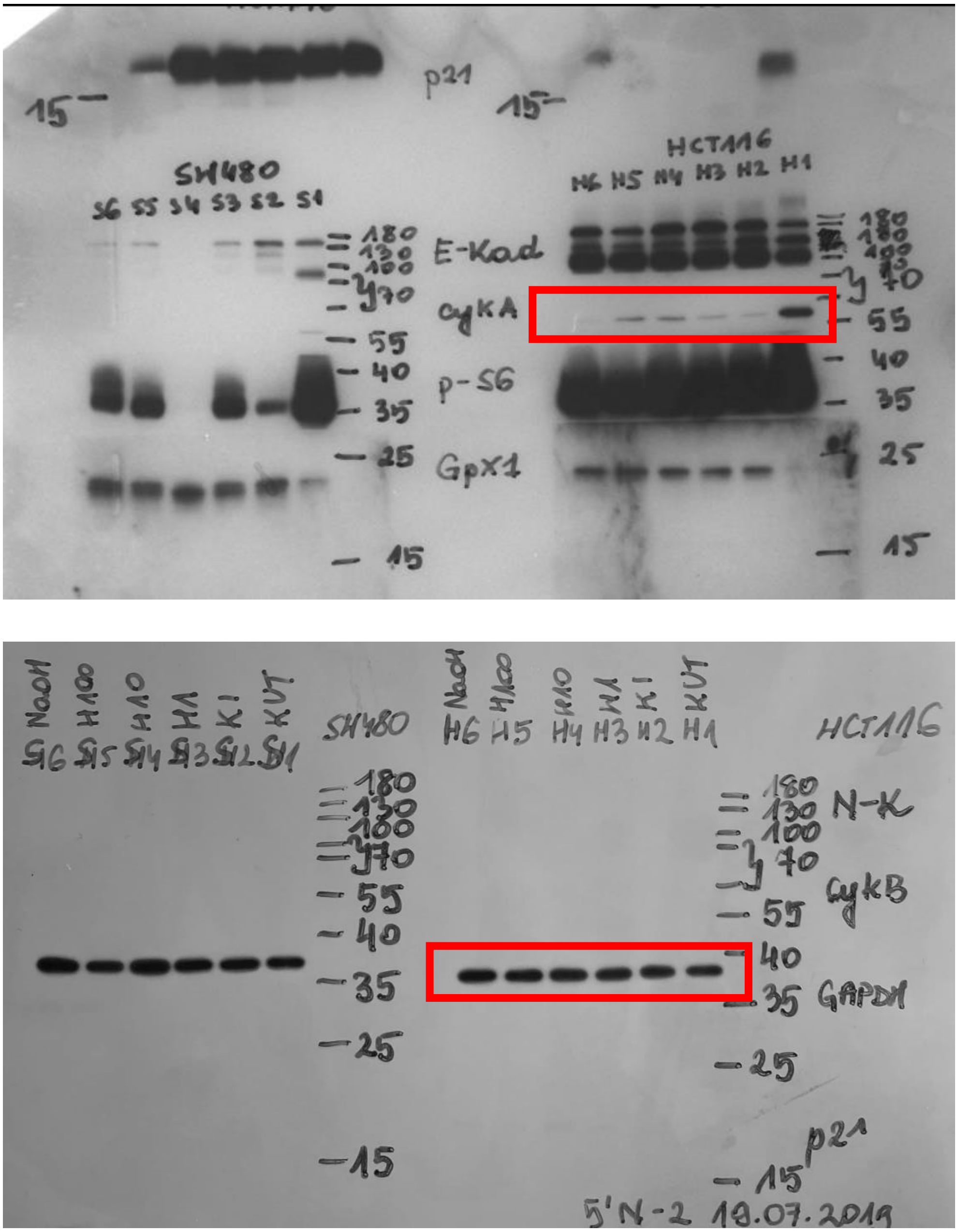

H1 – Untreated Control
H2 – Irinotecan
H3 – Irinotecan + 1 uM Hemin
H4 – Irinotecan + 10 uM Hemin
H5 – Irinotecan + 100 uM Hemin
H6 – Irinotecan + NaOH

Fig. A.II.4

cyclin B

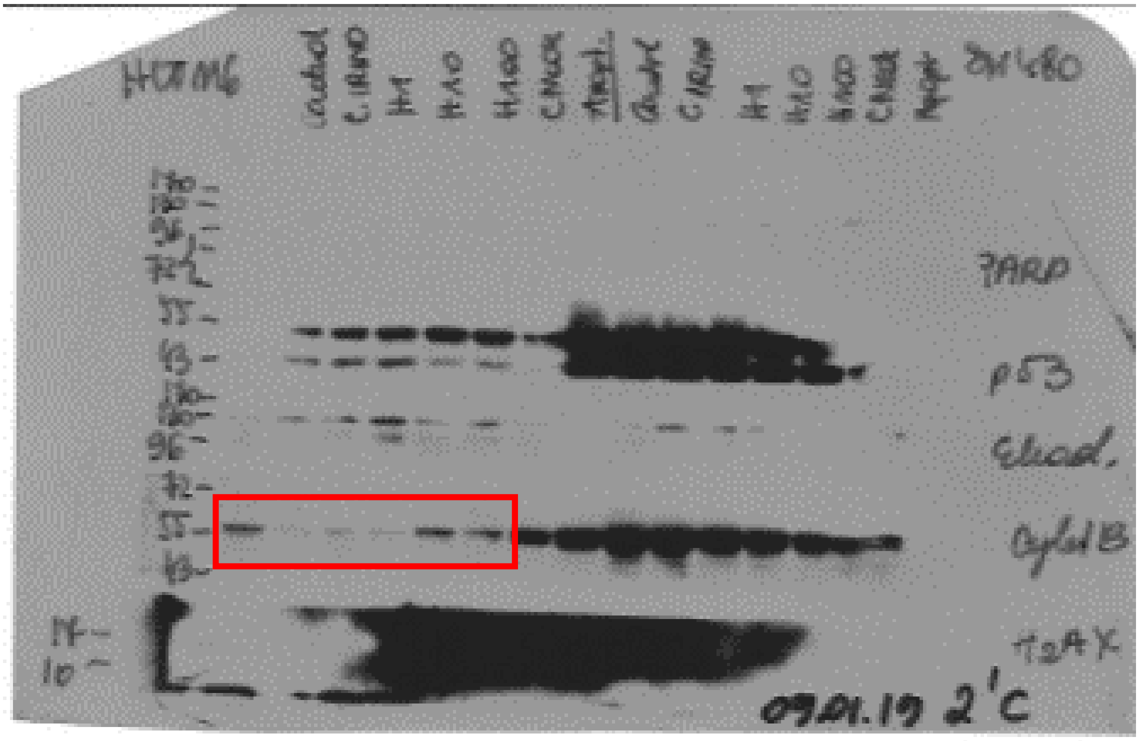

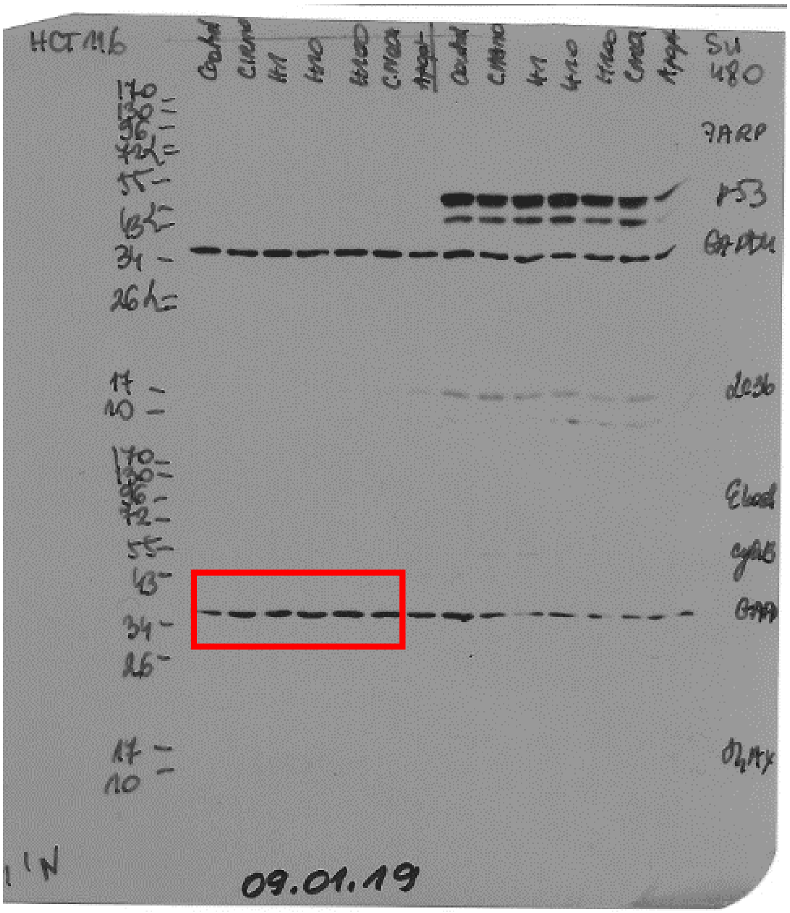

KM – Untreated Control
KS – Irinotecan
1 – Irinotecan + 1 uM Hemin
10 – Irinotecan + 10 uM Hemin
100 – Irinotecan + 100 uM Hemin
NaOH – Irinotecan + NaOH

Fig. A.II.5

p-cdc2

HCT116

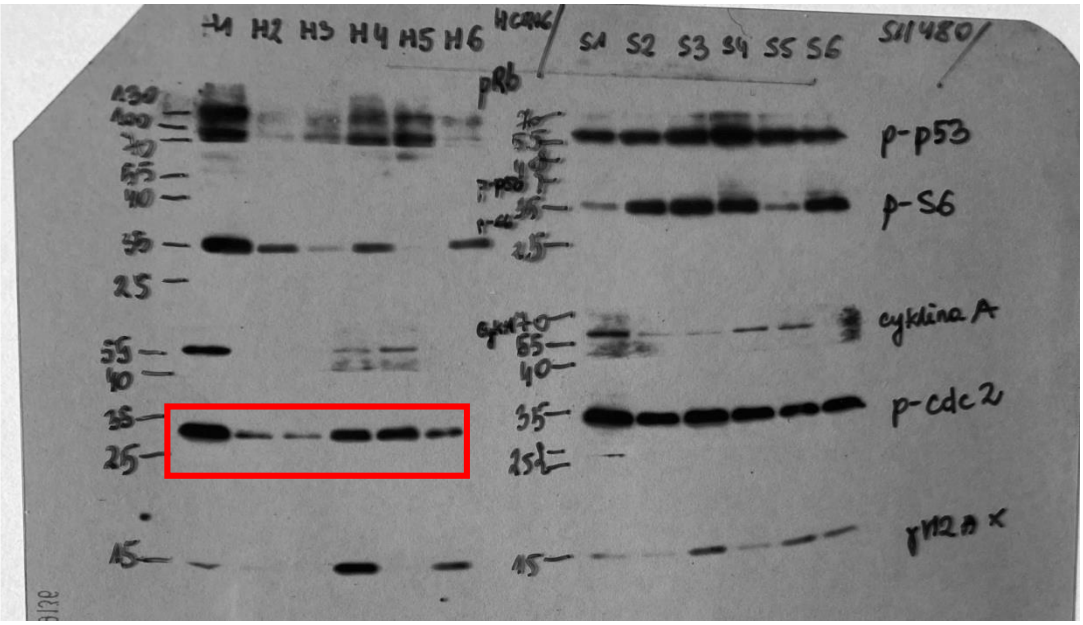

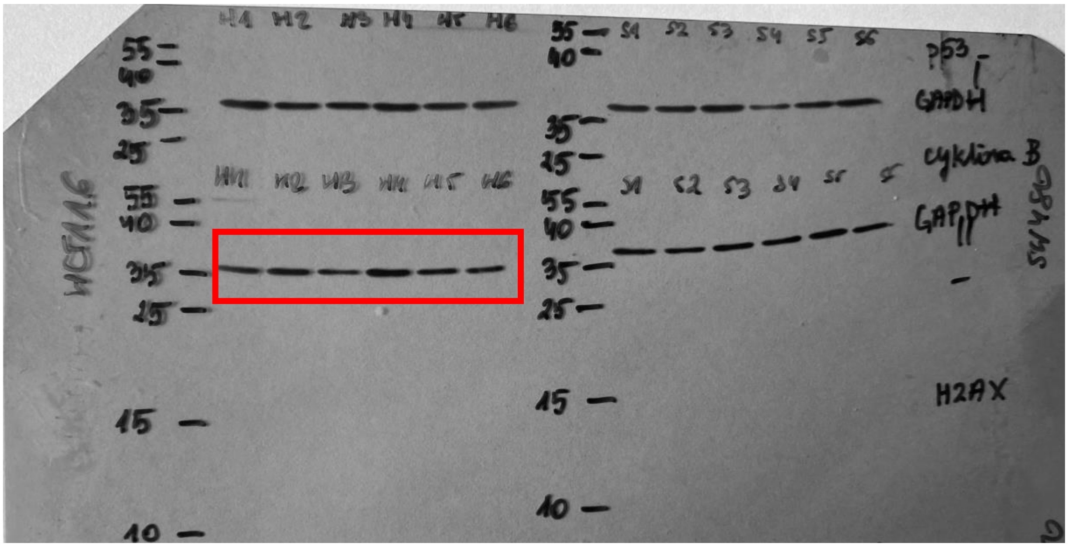

H1 – Untreated Control
H2 – Irinotecan
H3 – Irinotecan + 1 uM Hemin
H4 – Irinotecan + 10 uM Hemin
H5 – Irinotecan + 100 uM Hemin
H6 – Irinotecan + NaOH

Fig. A.II.6

p-Rb

HCT116

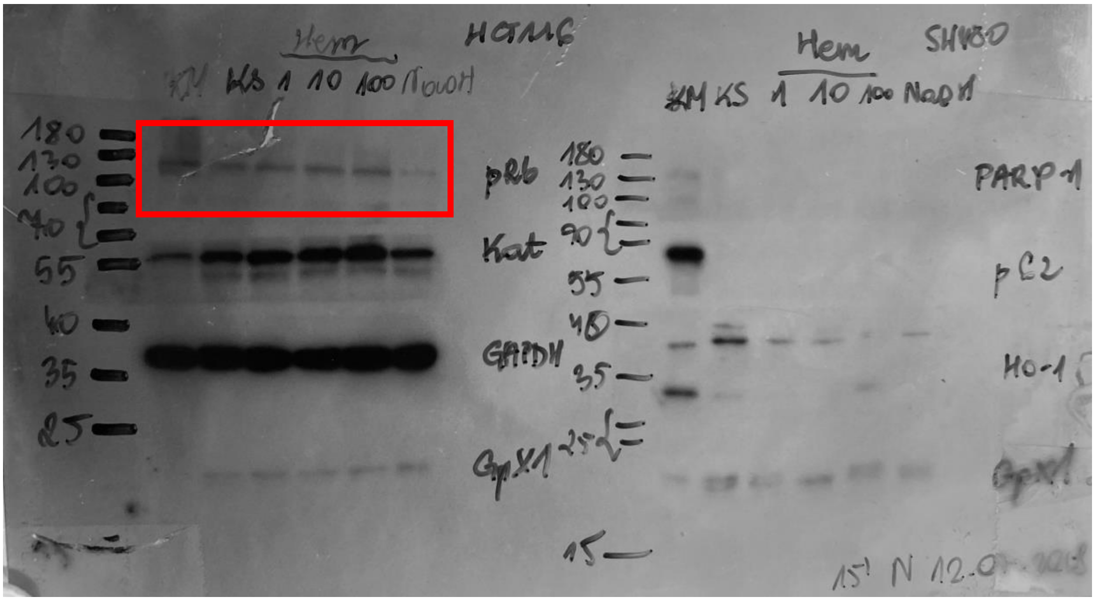

KM – Untreated Control
KS – Irinotecan
1 – Irinotecan + 1 uM Hemin
10 – Irinotecan + 10 uM Hemin
100 – Irinotecan + 100 uM Hemin
NaOH – Irinotecan + NaOH

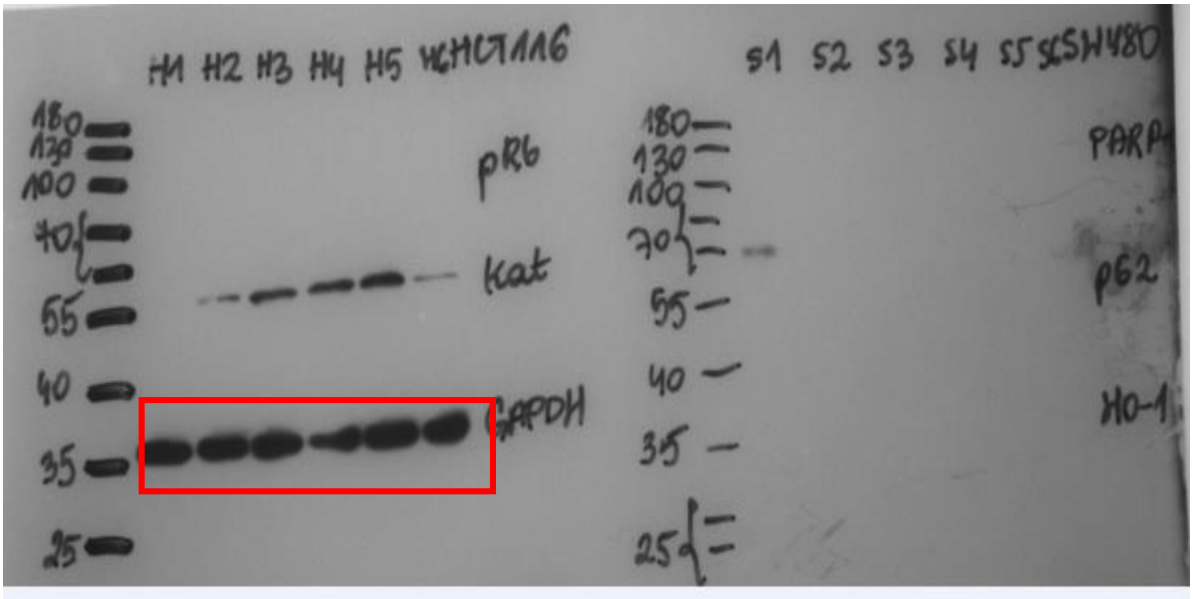

H1 – Untreated Control
H2 – Irinotecan
H3 – Irinotecan + 1 uM Hemin
H4 – Irinotecan + 10 uM Hemin
H5 – Irinotecan + 100 uM Hemin
H6 – Irinotecan + NaOH

Fig. A.II.7

p21

HCT116

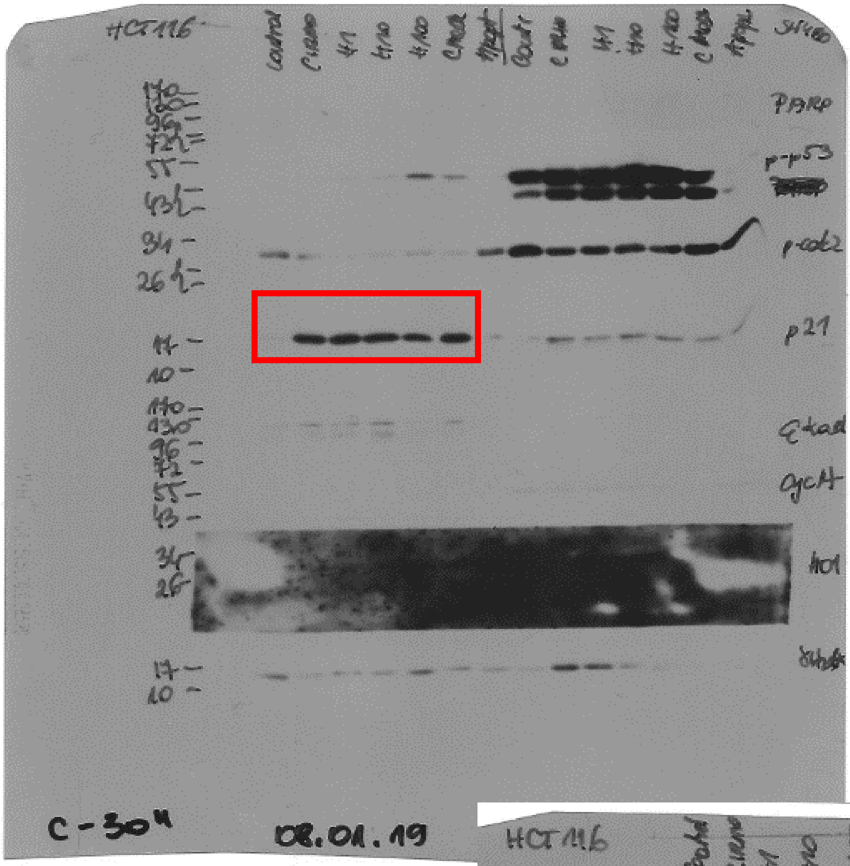

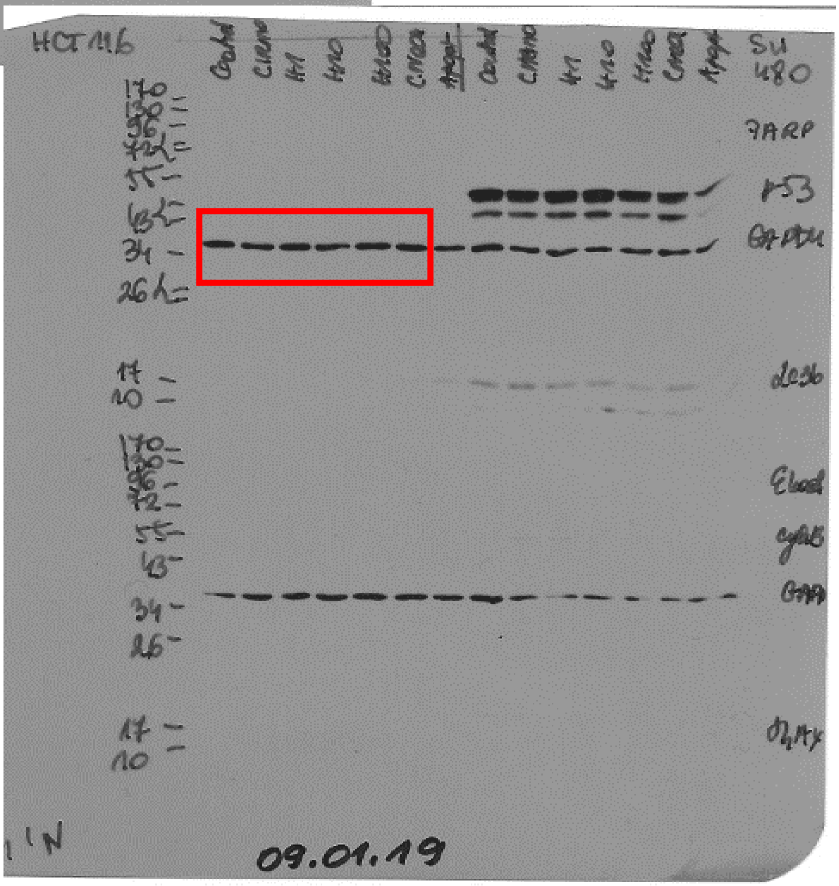

control – Untreated Control
C IRINO – Irinotecan
H1 – Irinotecan + 1 uM Hemin
H10 – Irinotecan + 10 uM Hemin
H100 – Irinotecan + 100 uM Hemin
NaOH – Irinotecan + NaOH

Fig. A.II.8

p-S6

HCT116

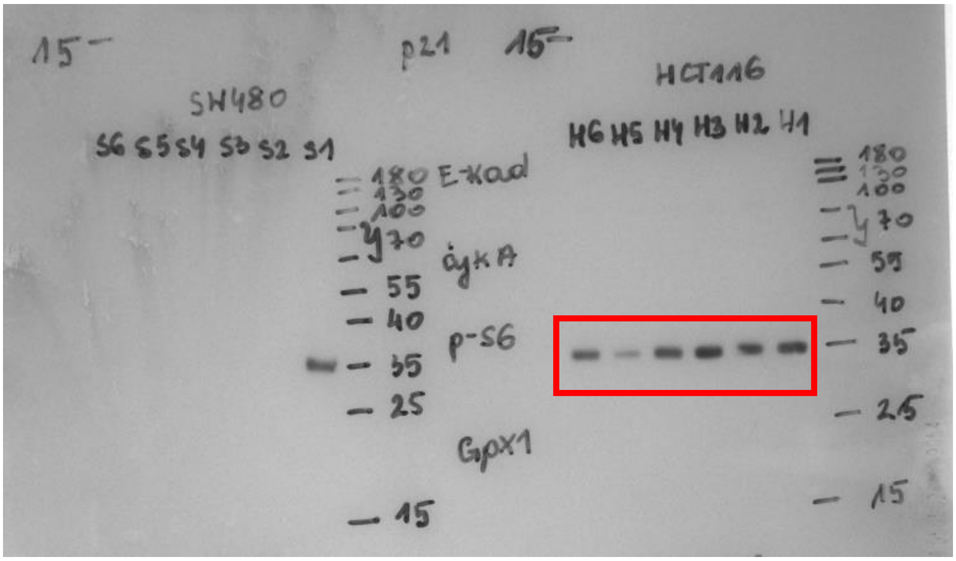

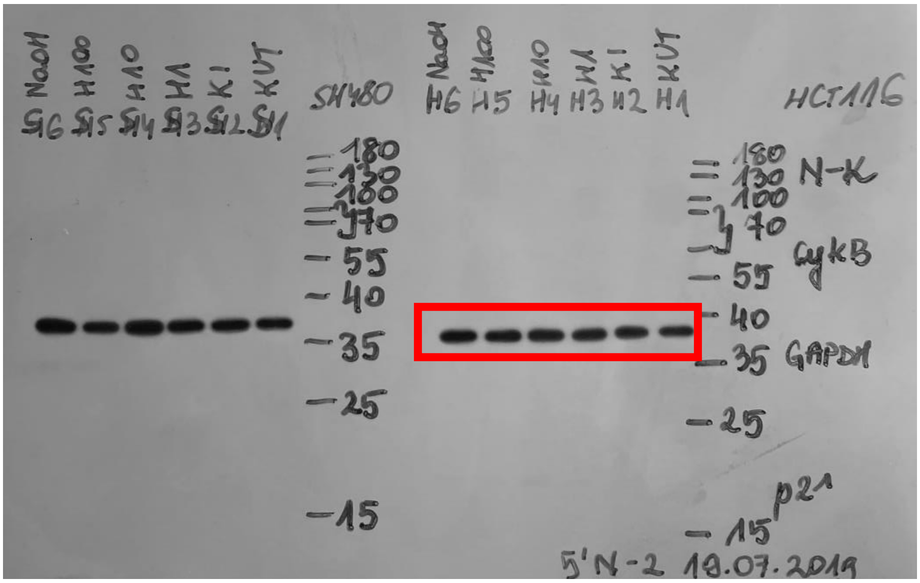

H1 – Untreated Control
H2 – Irinotecan
H3 – Irinotecan + 1 uM Hemin
H4 – Irinotecan + 10 uM Hemin
H5 – Irinotecan + 100 uM Hemin
H6 – Irinotecan + NaOH

Fig. A.II.9

PARP-1 **HCT116**

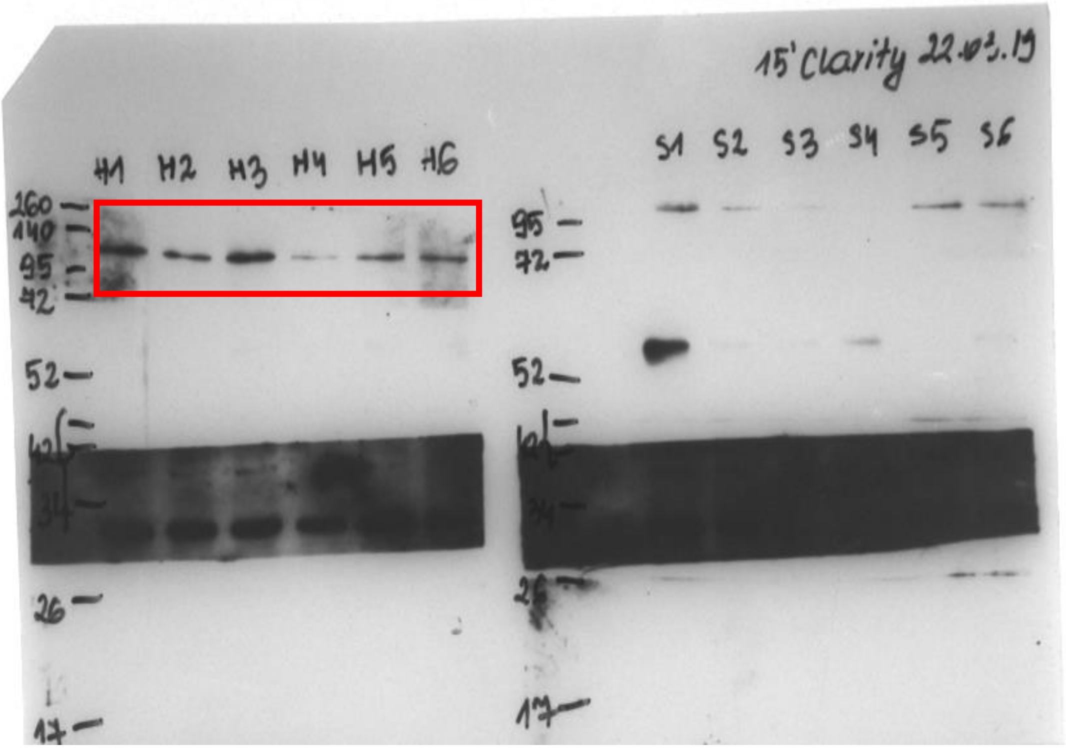

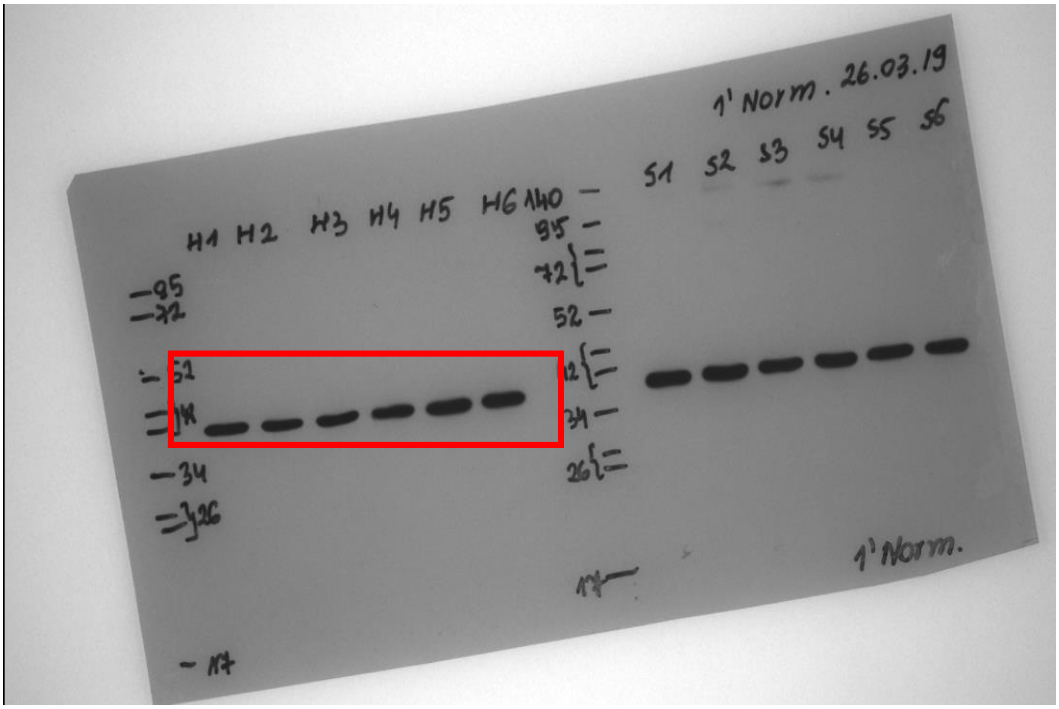

H1 – Untreated Control
H2 – Irinotecan
H3 – Irinotecan + 1 uM Hemin
H4 – Irinotecan + 10 uM Hemin
H5 – Irinotecan + 100 uM Hemin
H6 – Irinotecan + NaOH

Fig. A.II.10

γ-H2AX

HCT116

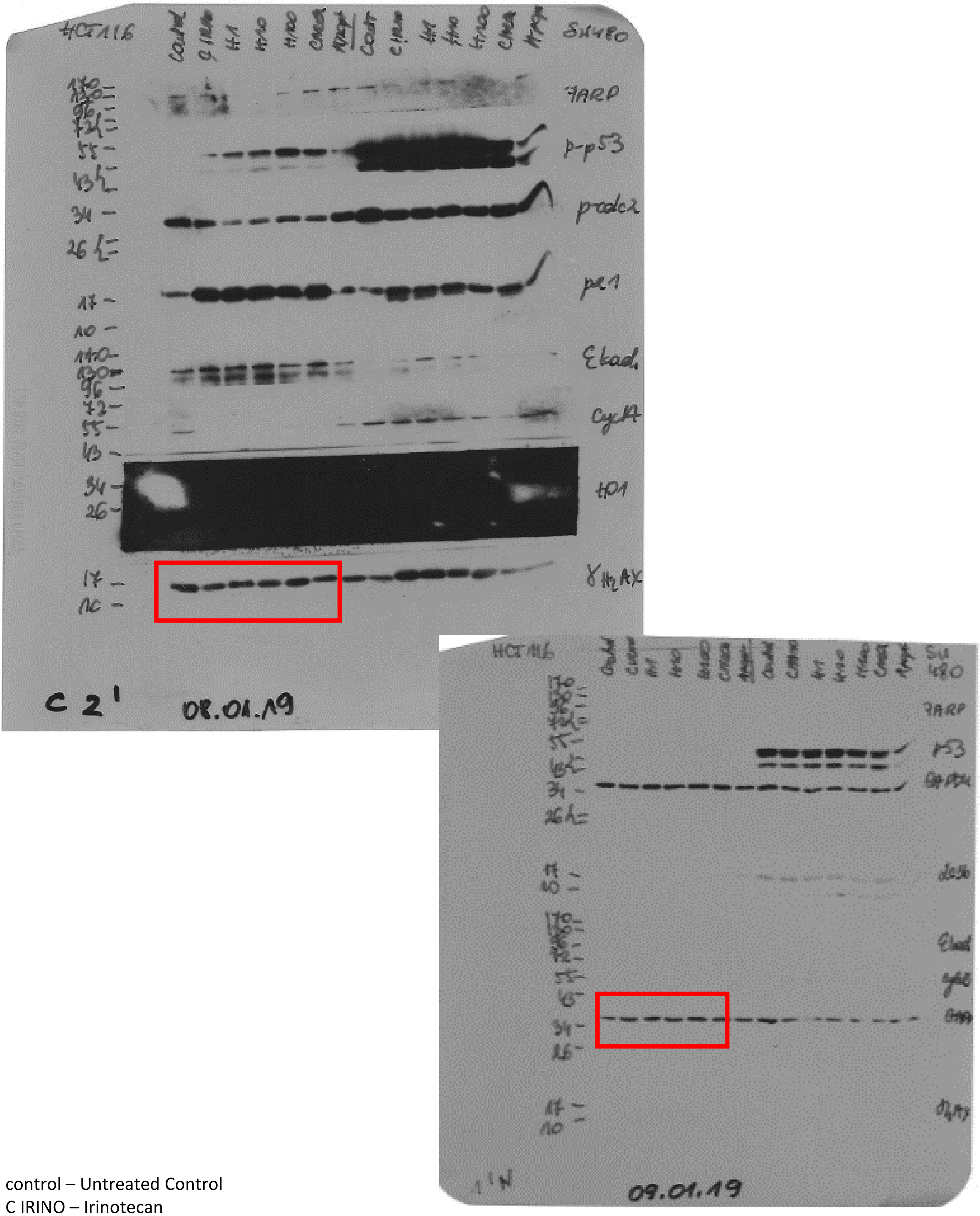

control – Untreated Control
C IRINO – Irinotecan
H1 – Irinotecan + 1 uM Hemin
H10 – Irinotecan + 10 uM Hemin
H100 – Irinotecan + 100 uM Hemin
NaOH – Irinotecan + NaOH

Fig. A.II.11

HO-1

control | 1+4 | H_2O_2 10 | control | 1+7 | H_2O_2 10

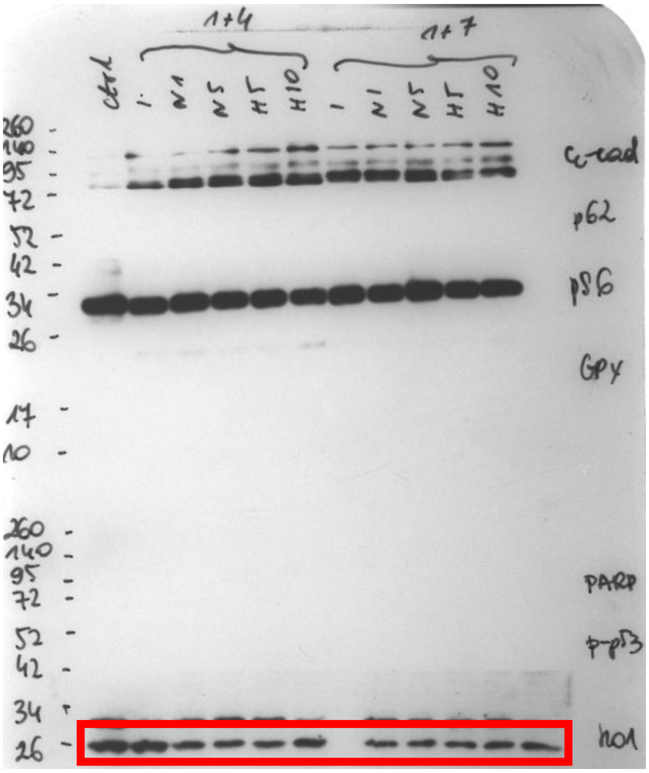

control | 1+4 | H_2O_2 10 | control | 1+7 | H_2O_2 10

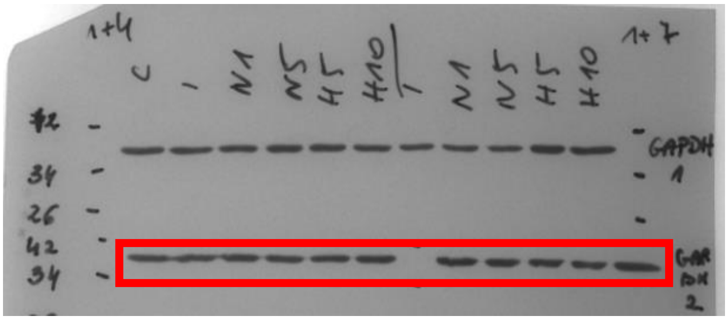

Fig. A.II.12

CAT

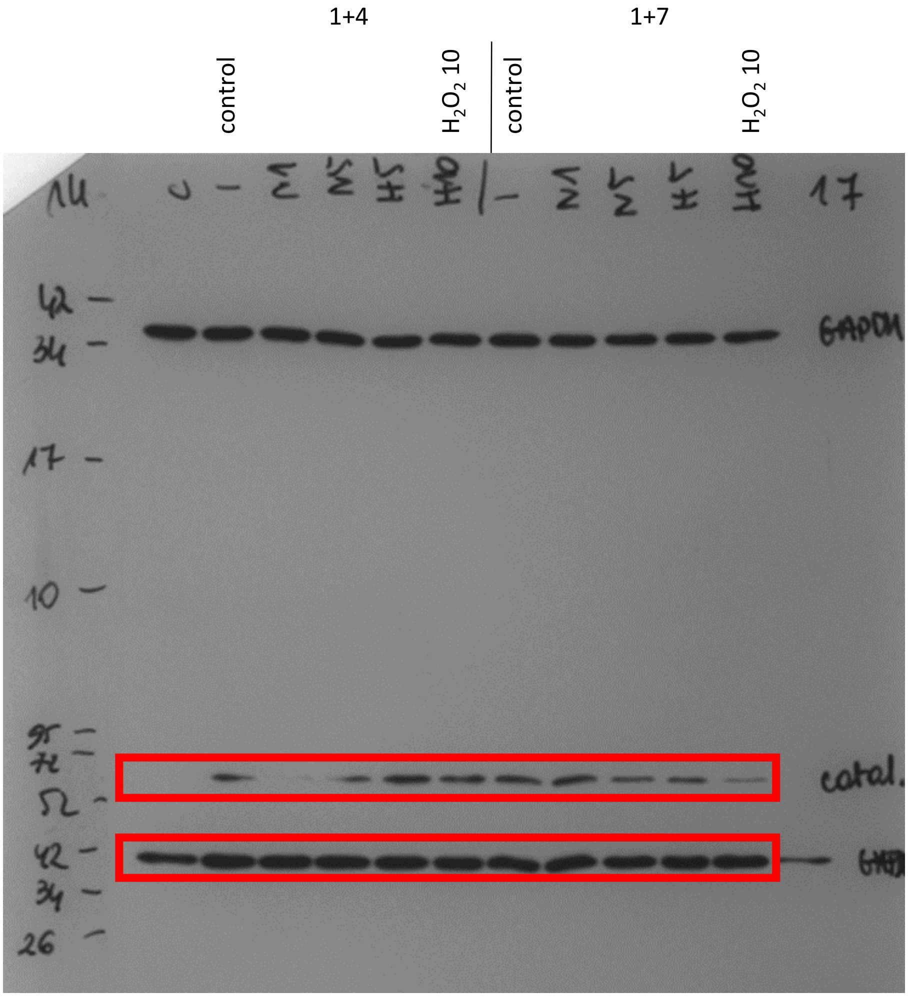

Fig. A.II.13

GPx-1

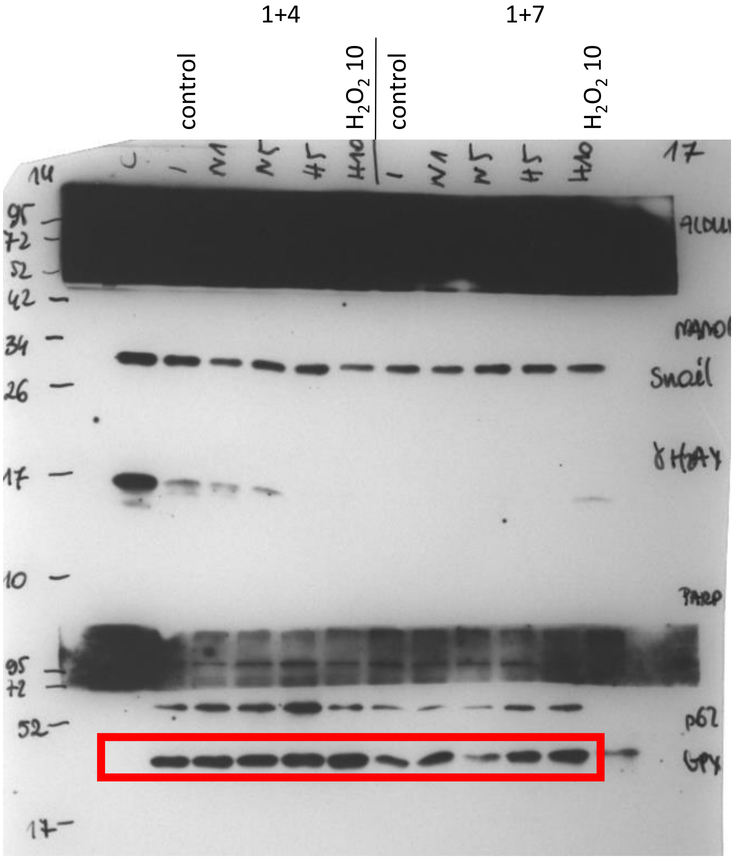

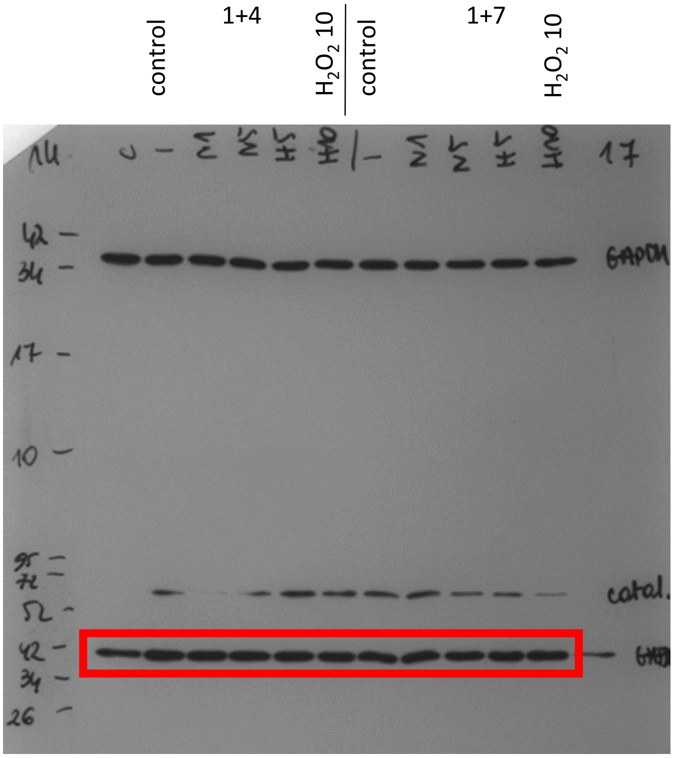

Fig. A.II.14

cyclin A

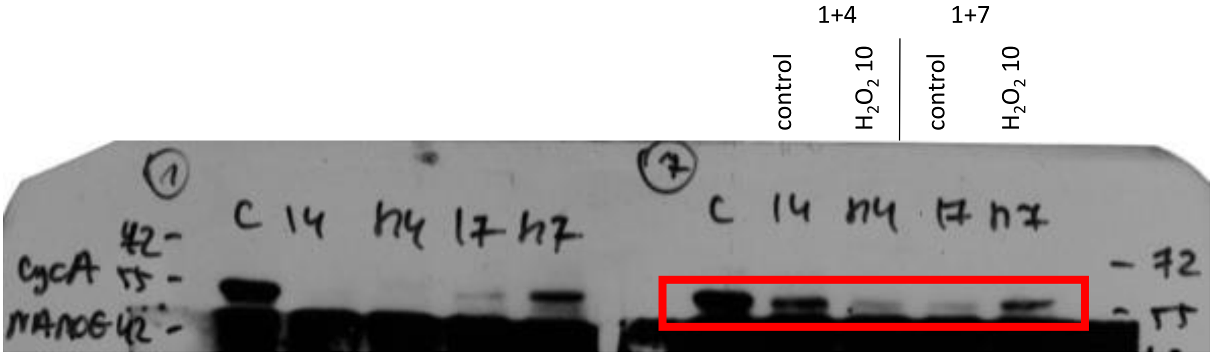

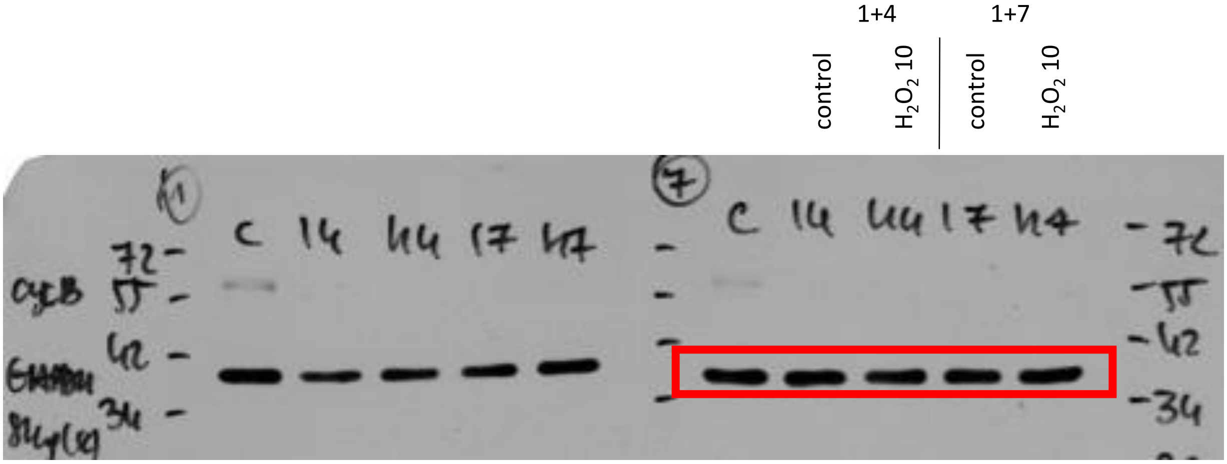

Fig. A.II.15

cyclin B

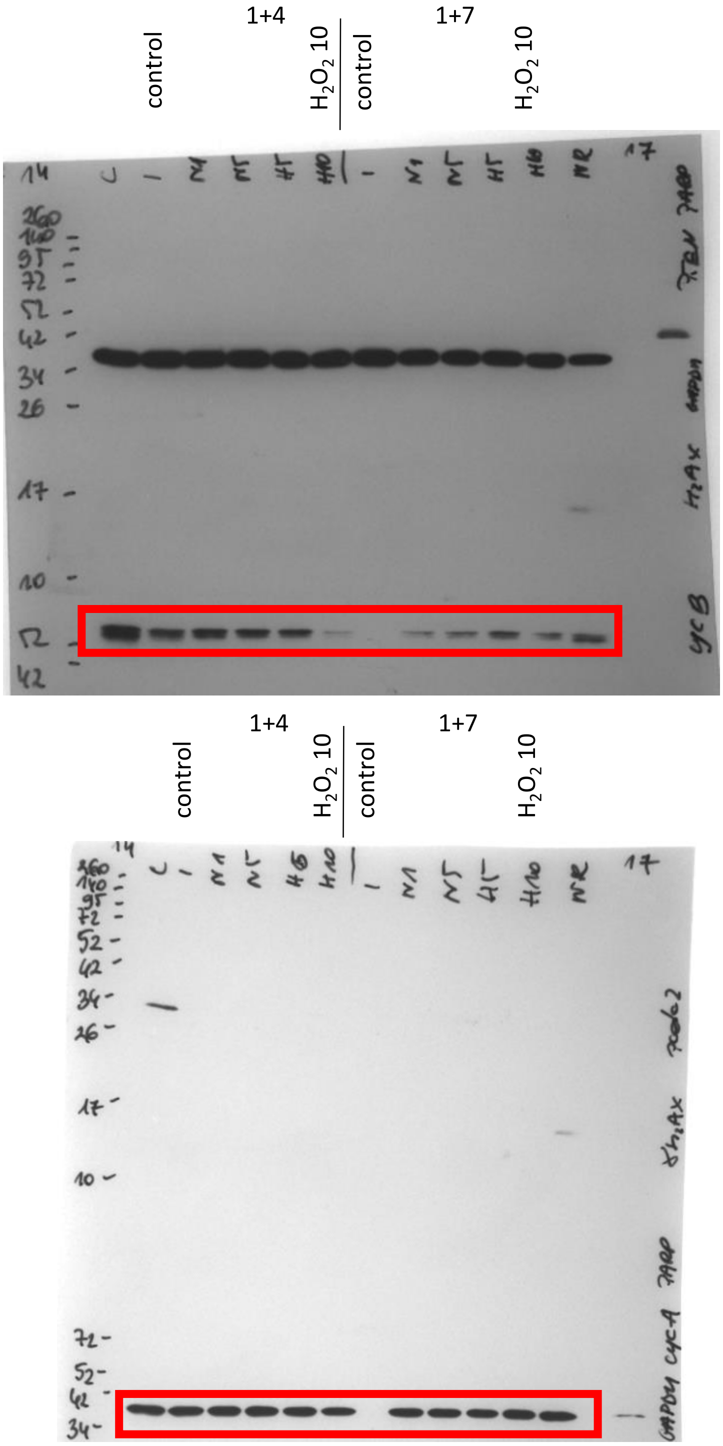

Fig. A.II.16

p-cdc2

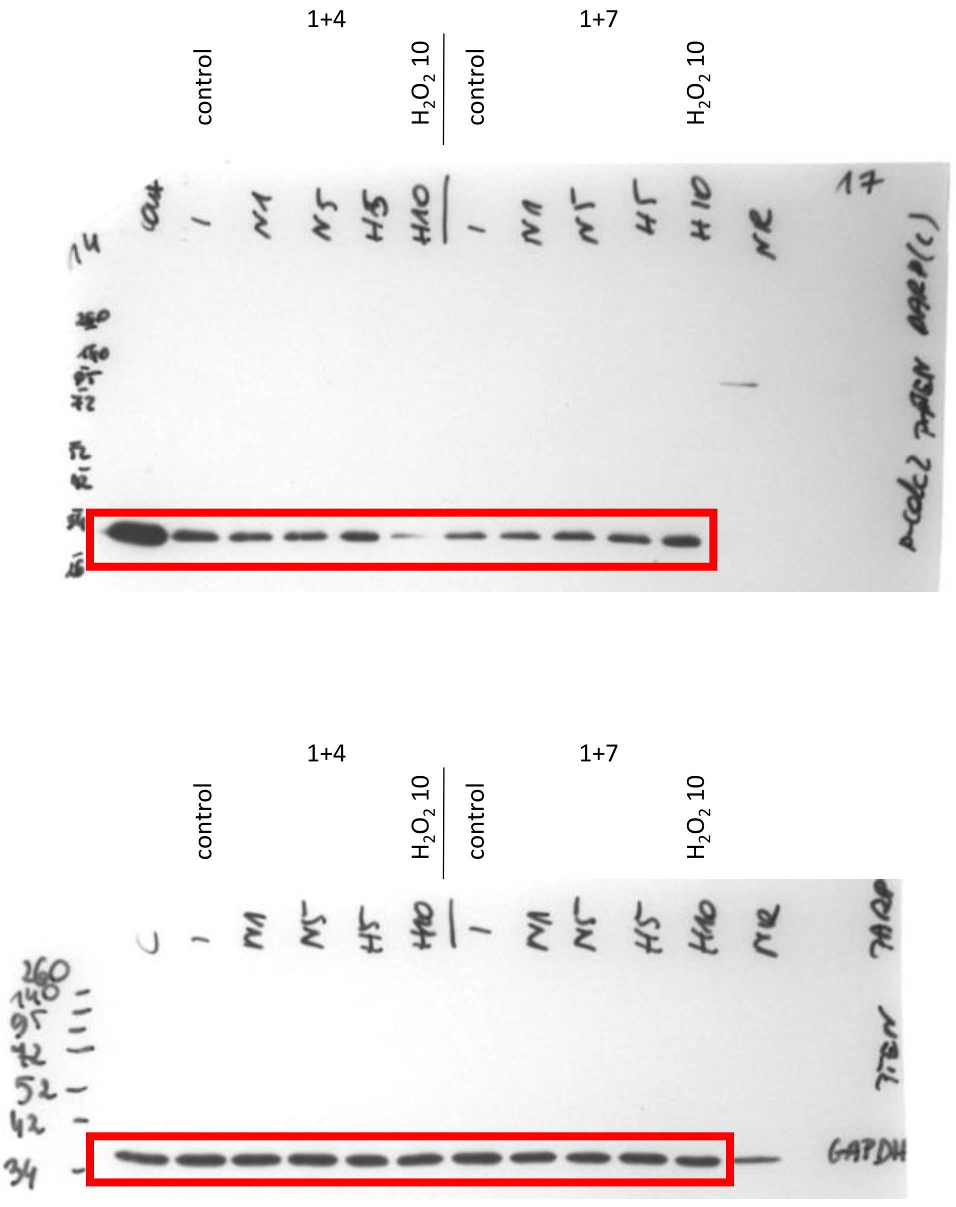

Fig. A.II.17

p-Rb

1+7 | 1+4

H_2O_2 10 | control | H_2O_2 10 | control

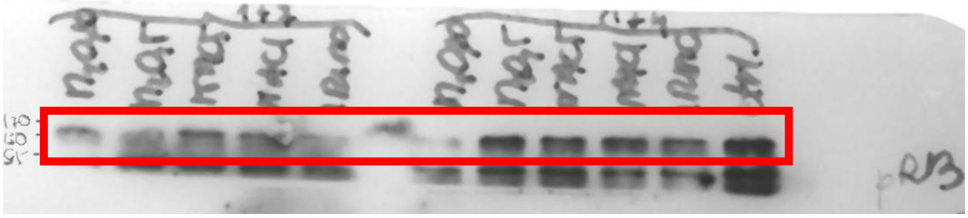

1+7 | 1+4

H_2O_2 10 | control | H_2O_2 10 | control

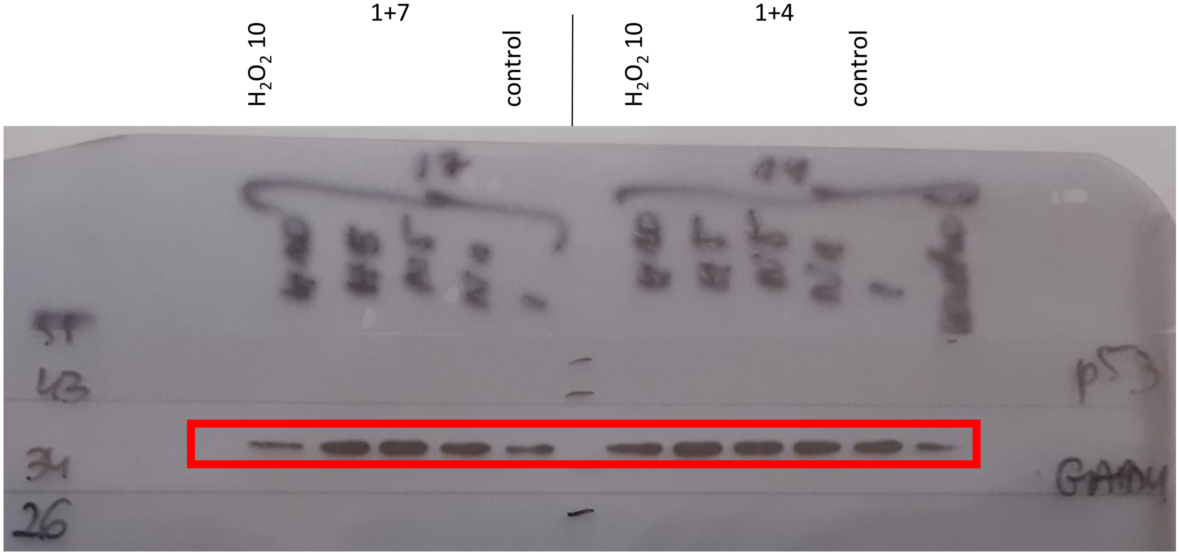

Fig. A.II.18

p21

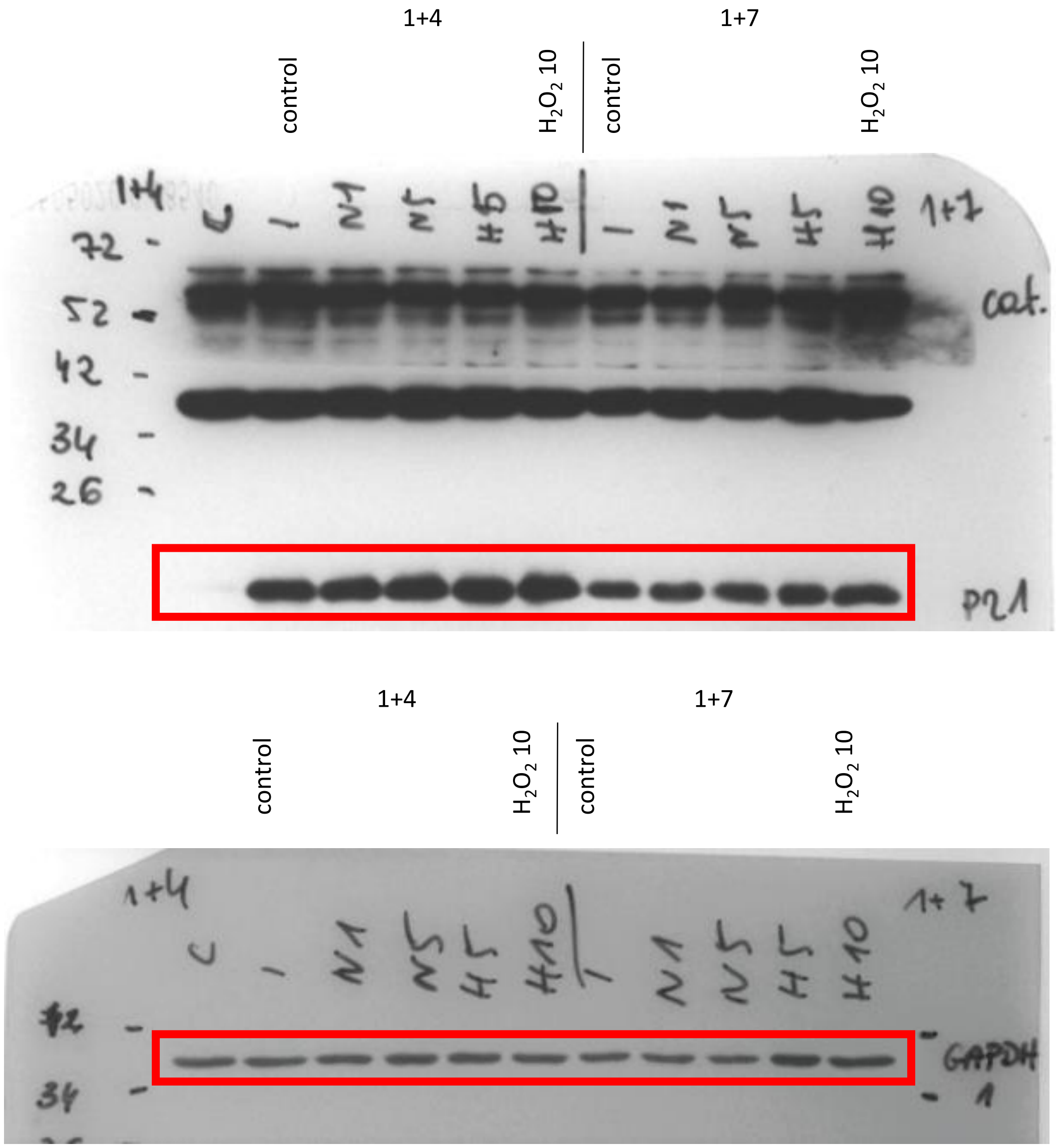

Fig. A.II.19

p-S6

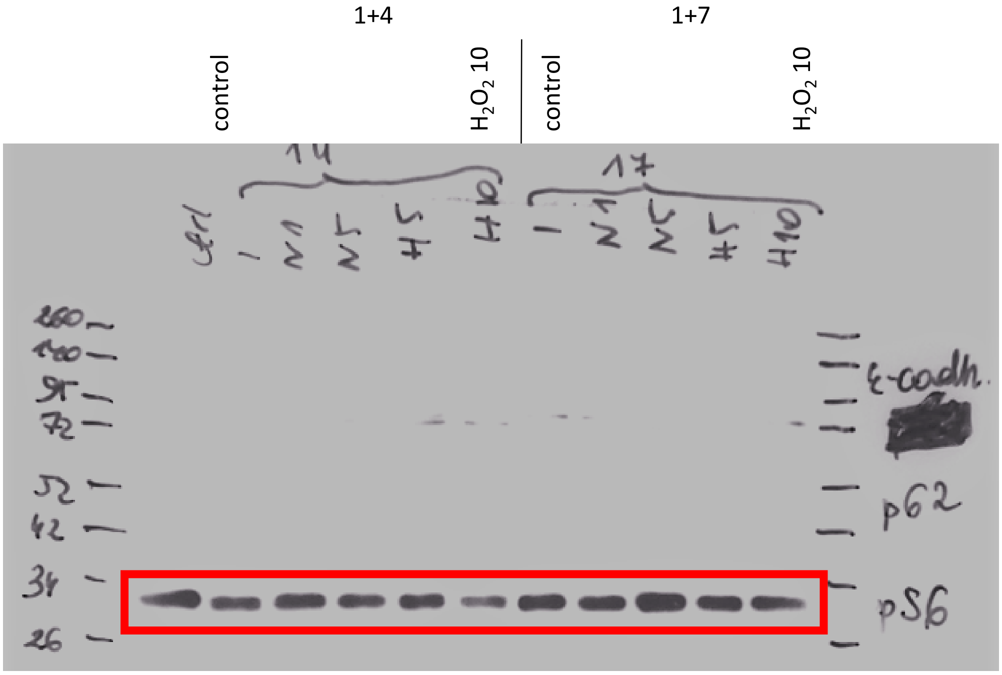

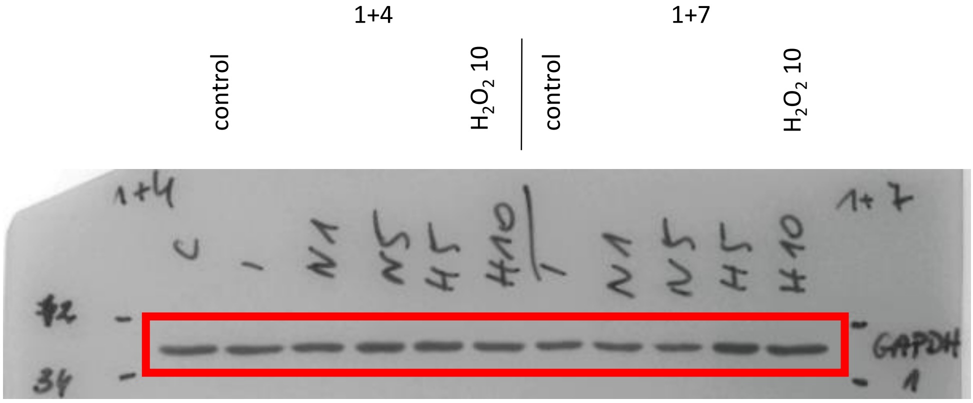

Fig. A.II.20

p53

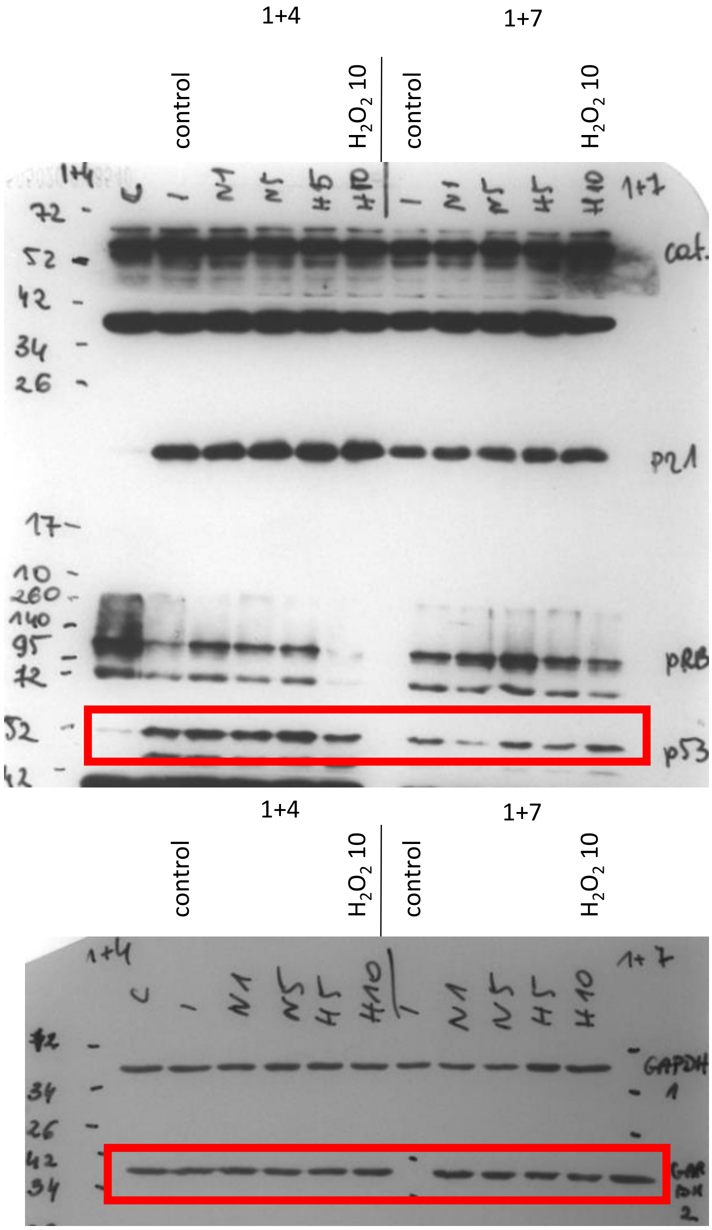

Fig. A.II.21

E-cadherin

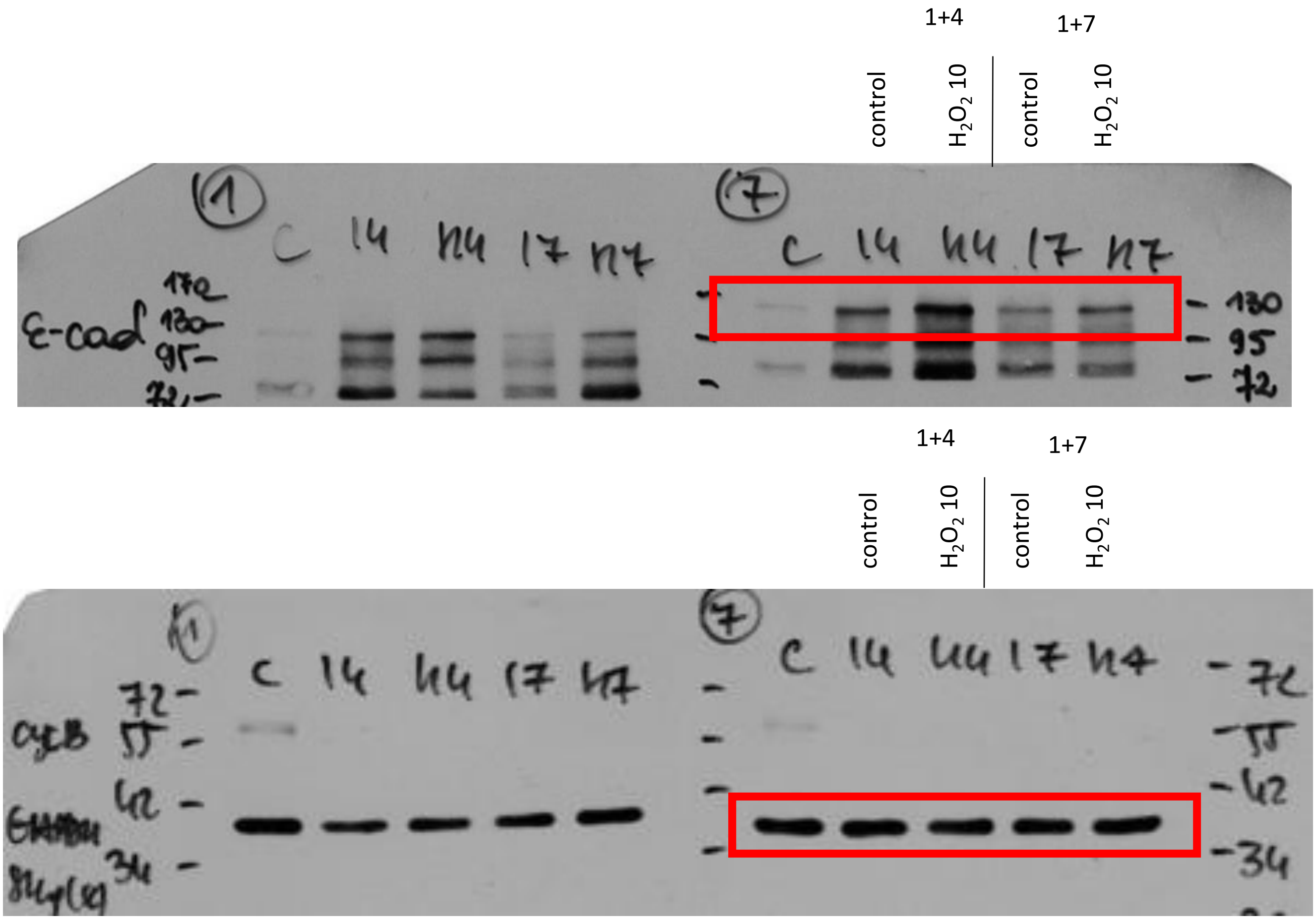

Fig. A.II.22

Snail

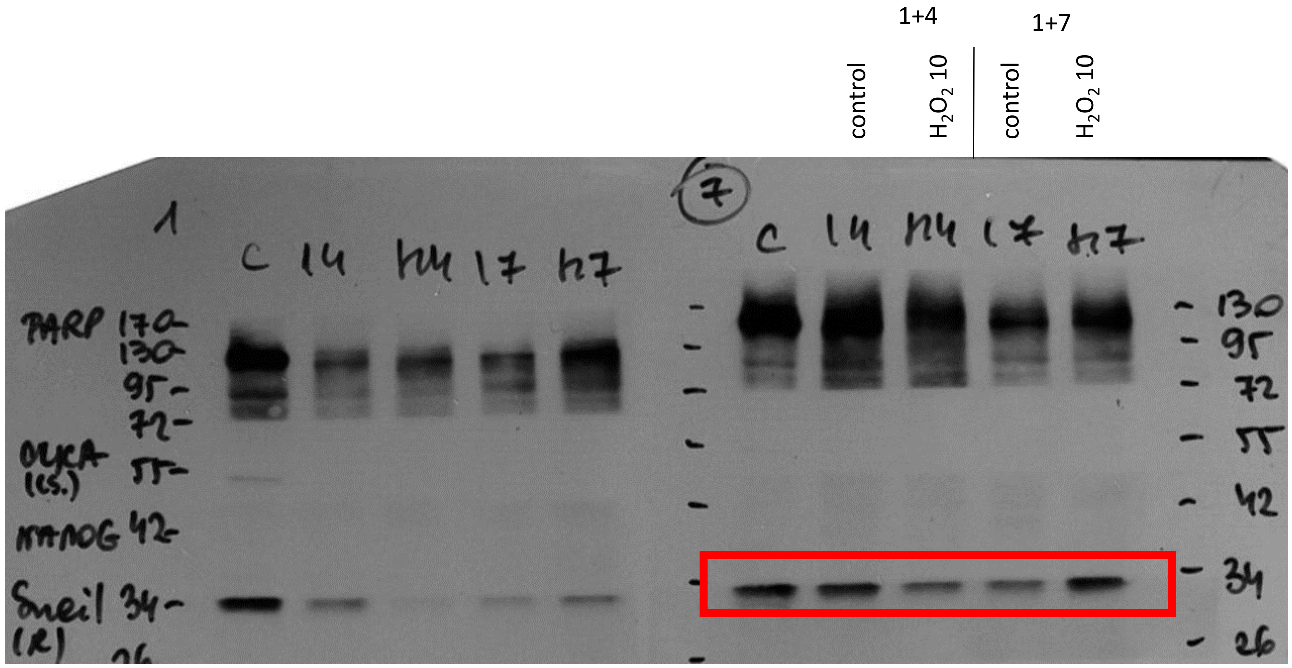

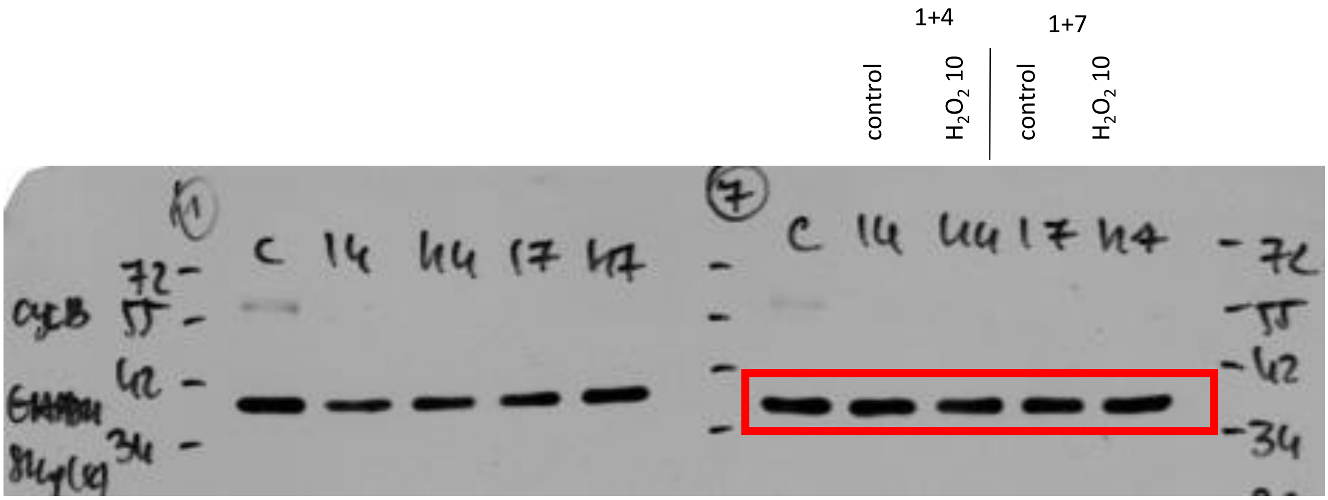

Fig. A.II.23

NANOG

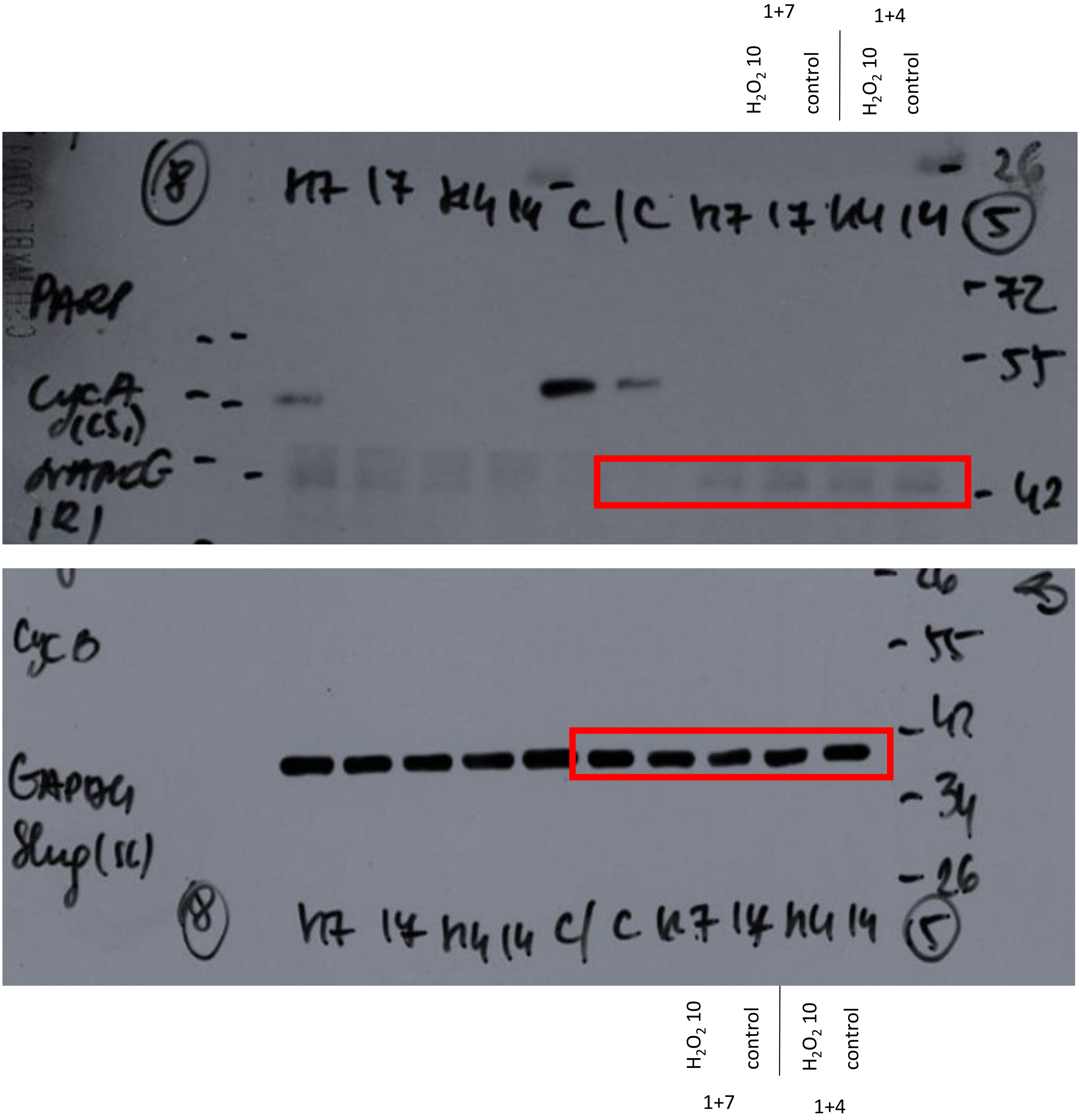

Fig. A.II.24

HO-1

HO-1

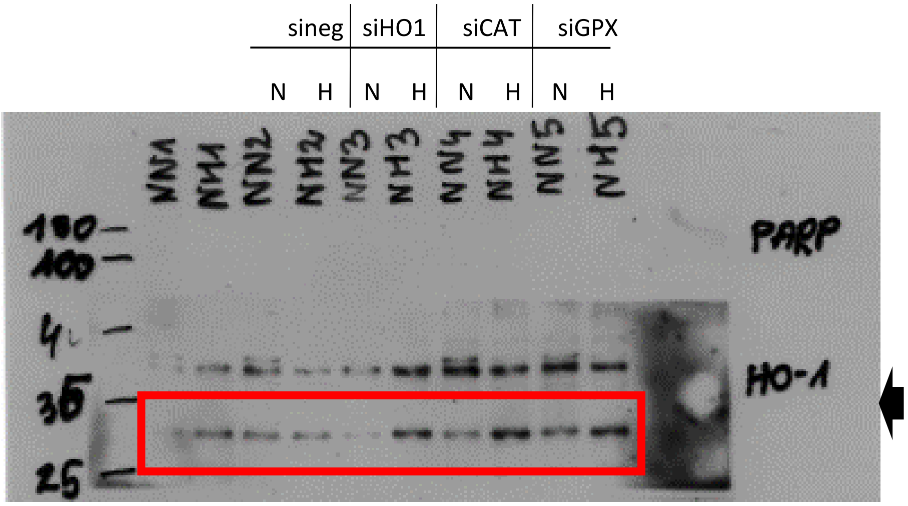

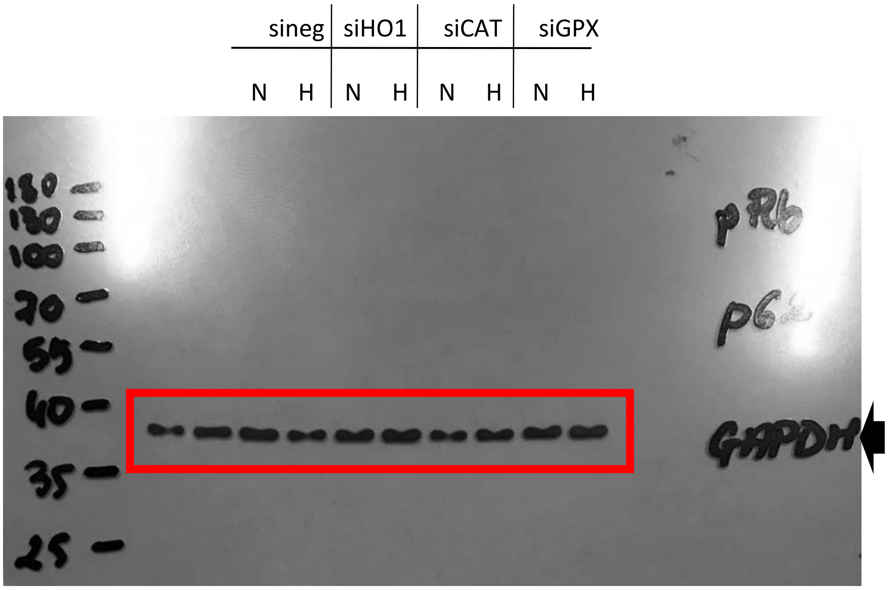

Fig. A.II.25

CAT

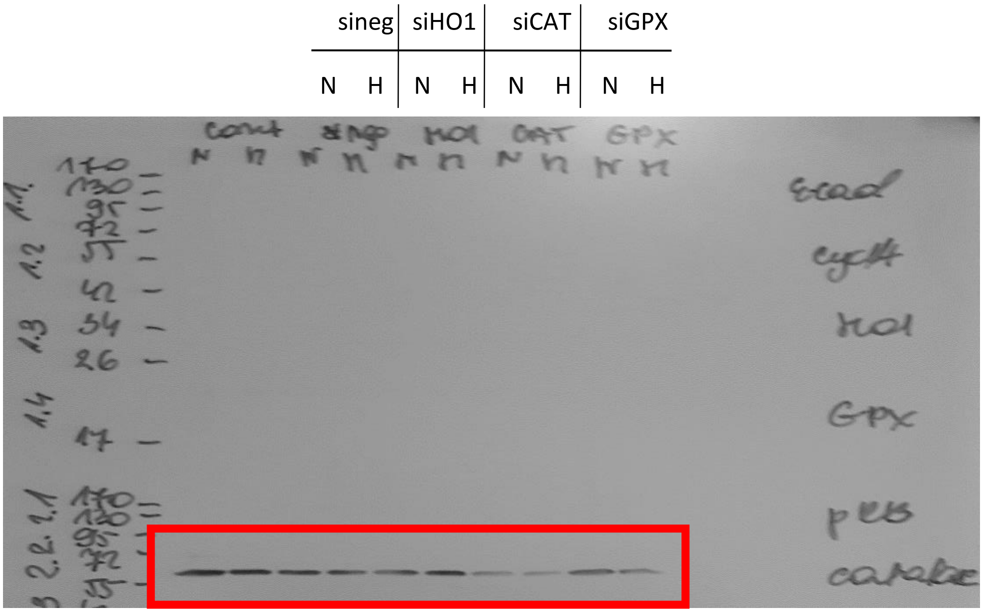

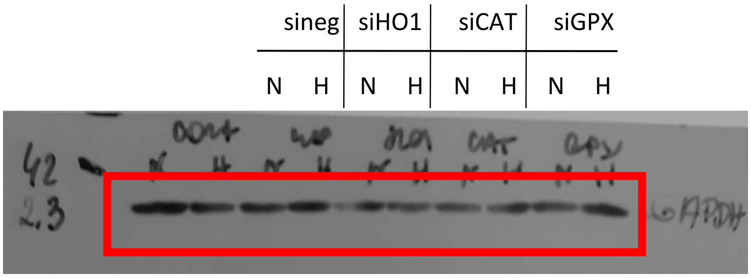

Fig. A.II.26

GPx-1

sineg		siHO1		siCAT		siGPX	
N	H	N	H	N	H	N	H

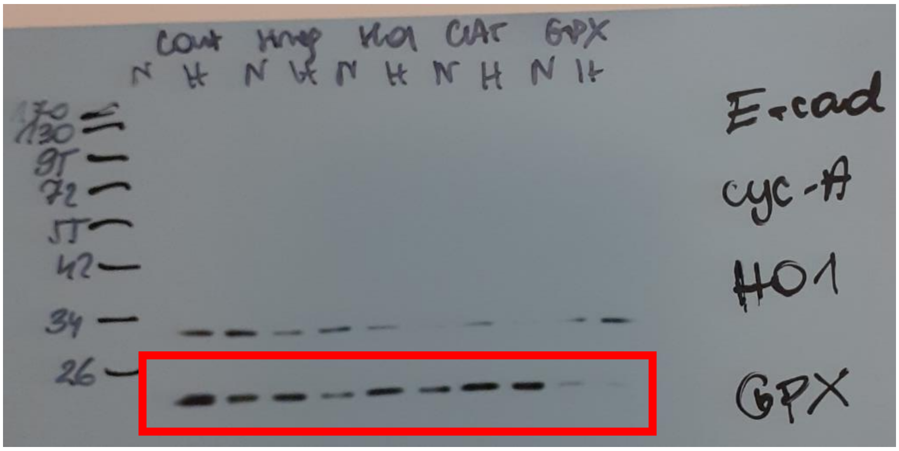

sineg		siHO1		siCAT		siGPX	
N	H	N	H	N	H	N	H

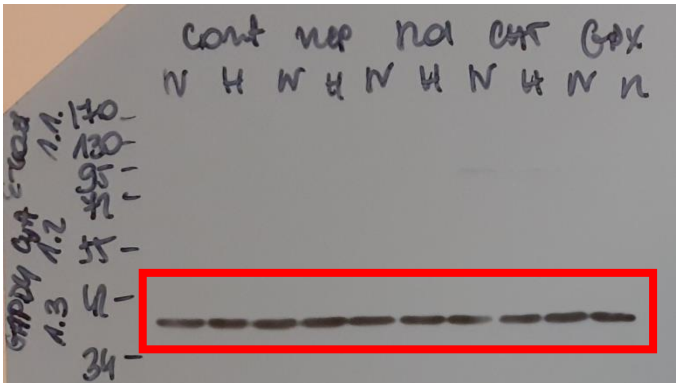

Fig. A.II.27

cyclin A

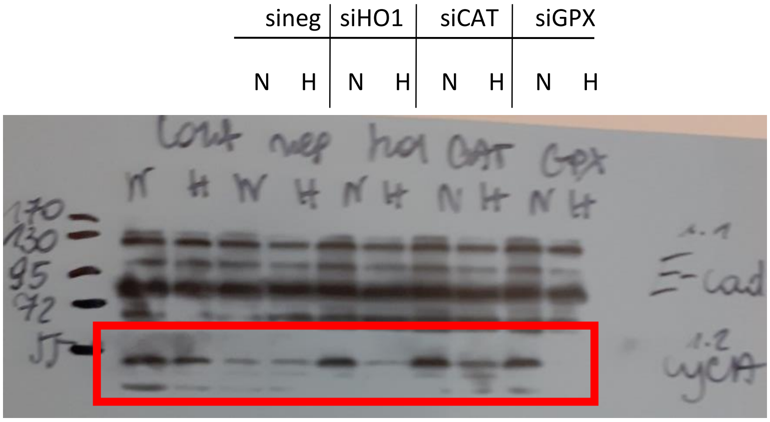

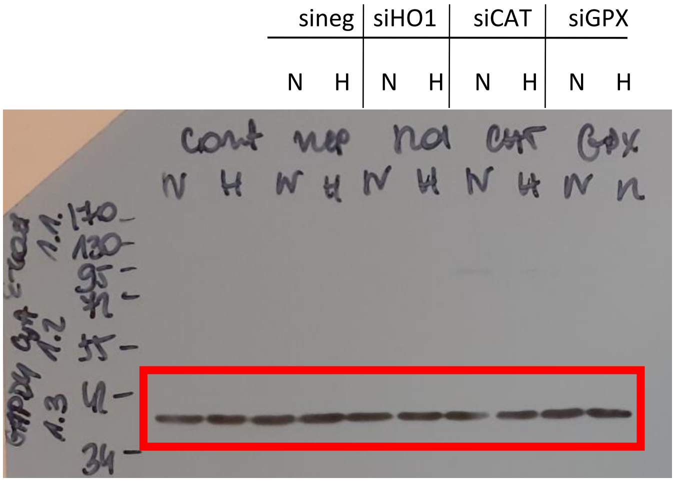

Fig. A.II.28

cyclin B

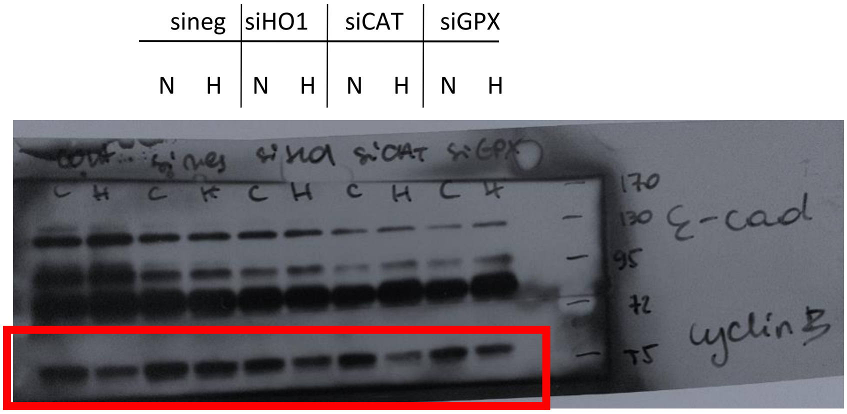

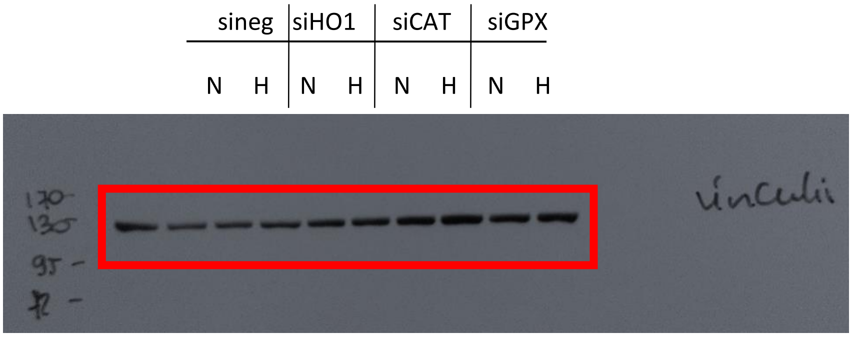

Fig. A.II.29

p-cdc2

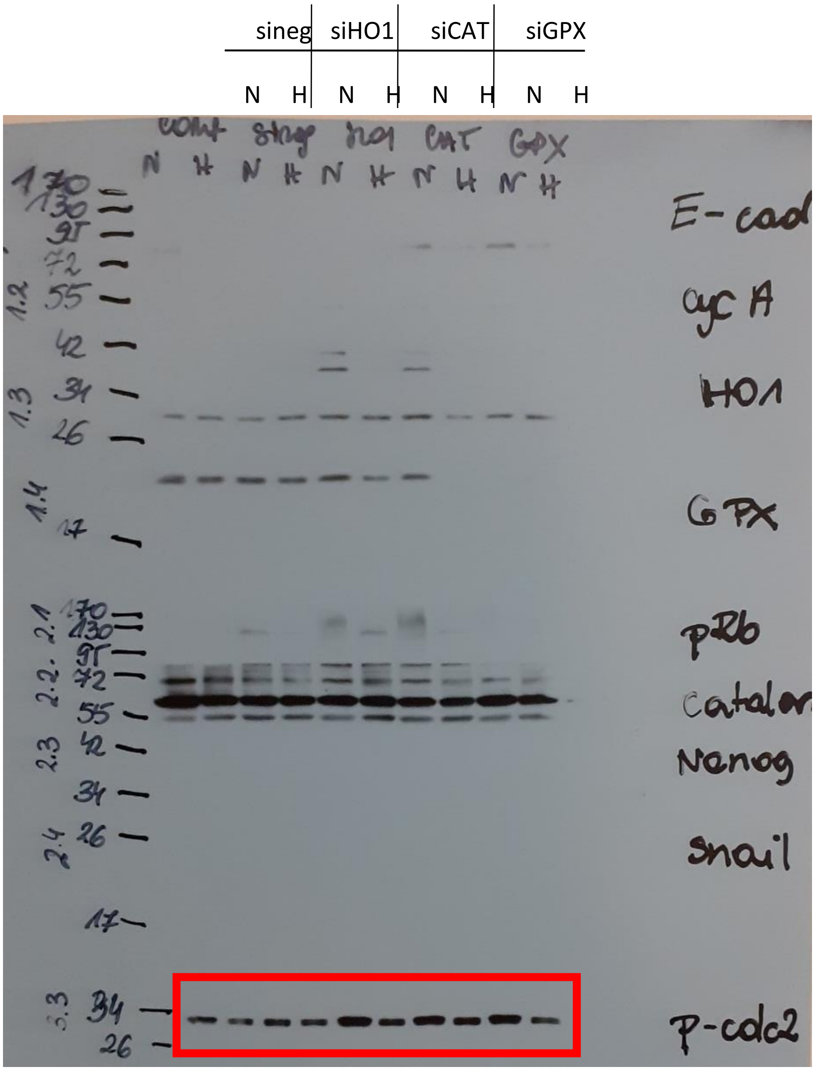

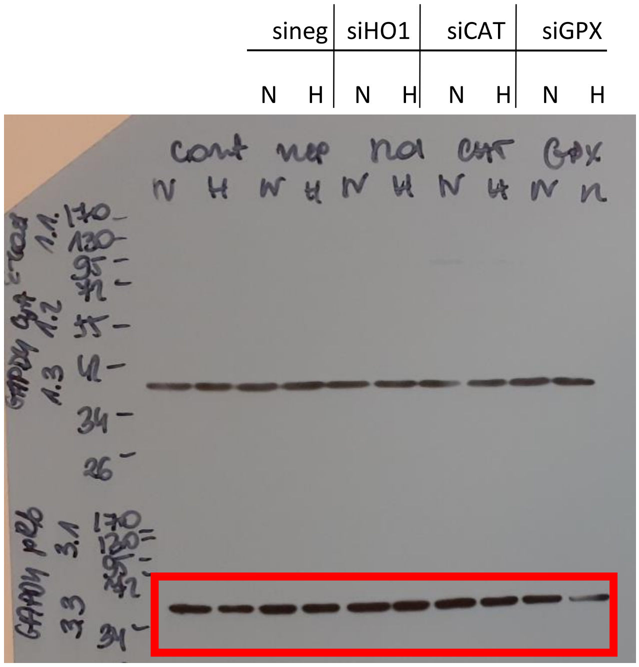

Fig. A.II.30

p-Rb

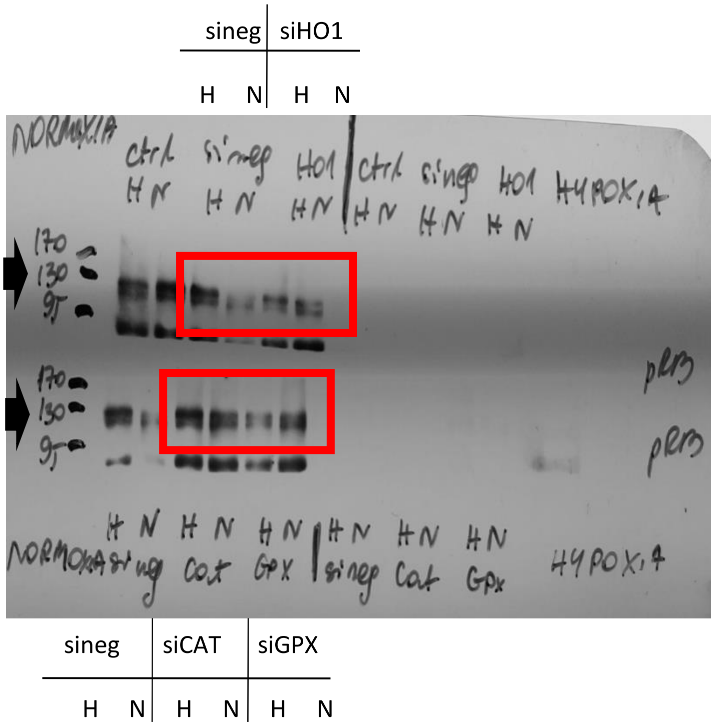

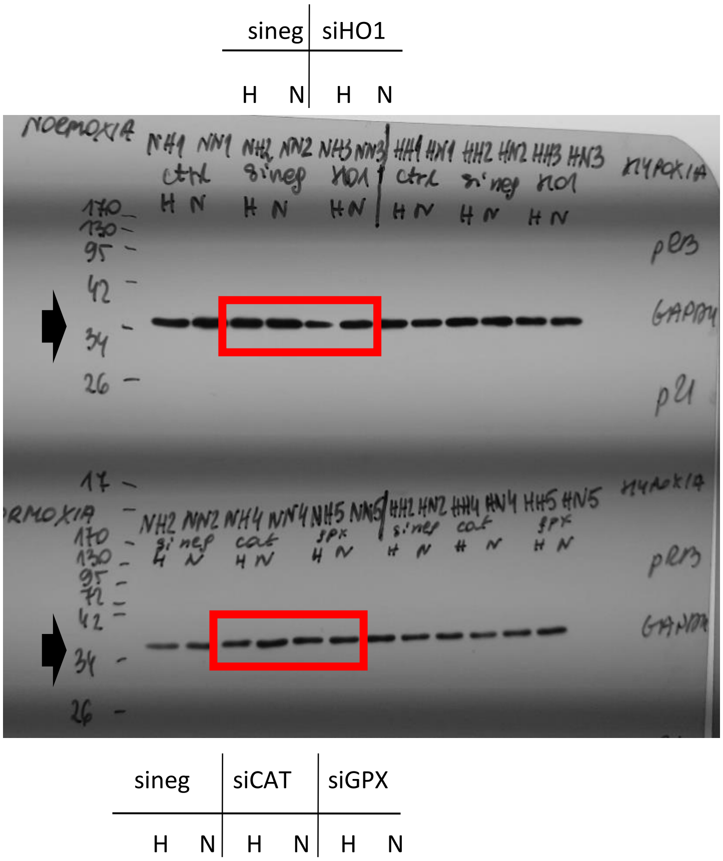

Fig. A.II.31

sineg		siHO1		siCAT		siGPX	
N	H	N	H	N	H	N	H

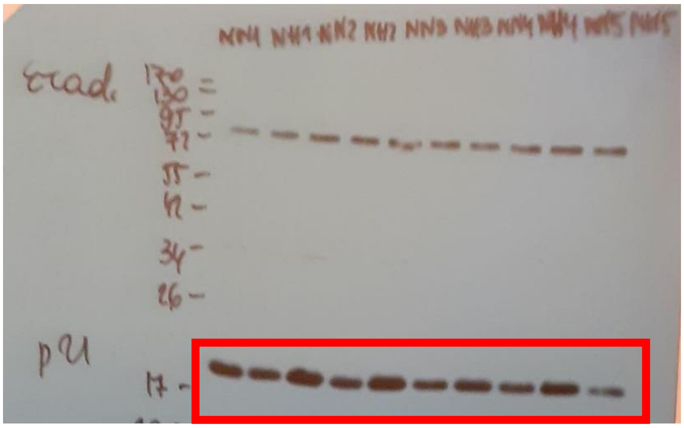

sineg		siHO1		siCAT		siGPX	
N	H	N	H	N	H	N	H

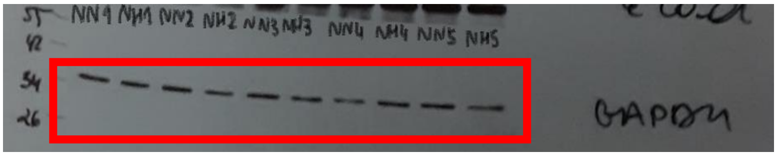

Fig. A.II.32

p-S6

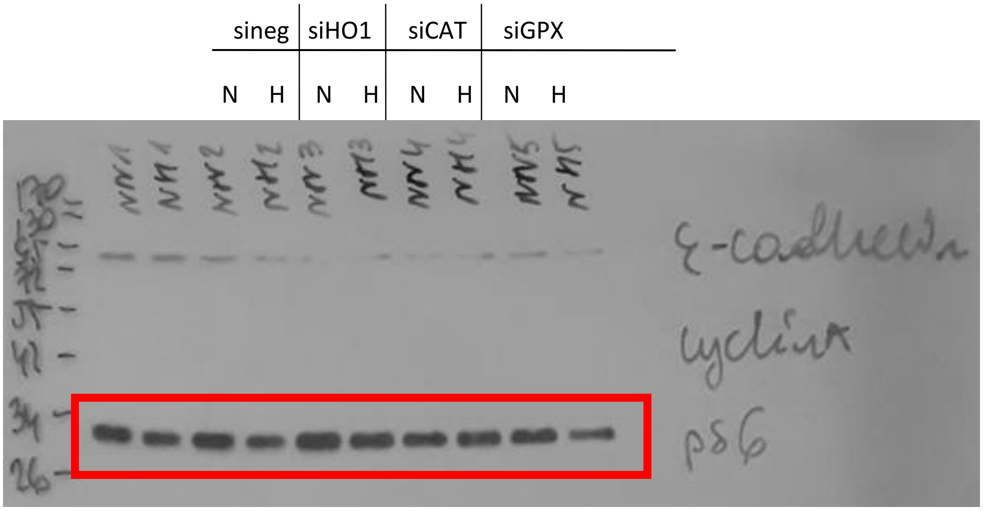

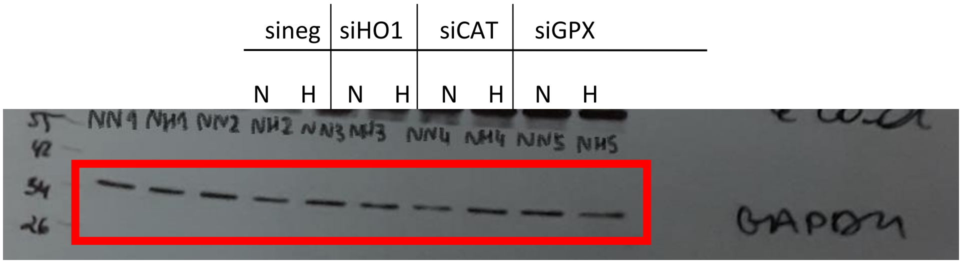

Fig. A.II.33

PARP-1

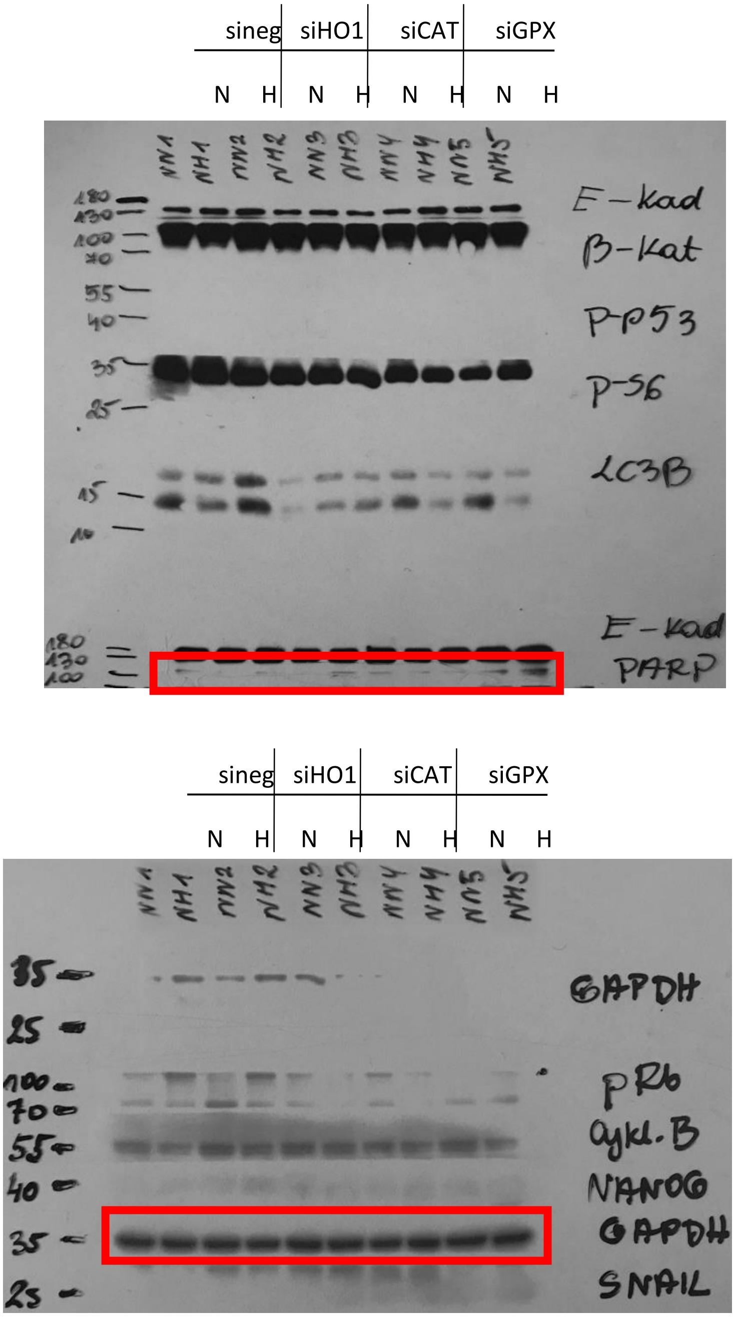

Fig. A.II.34

E-cadherin

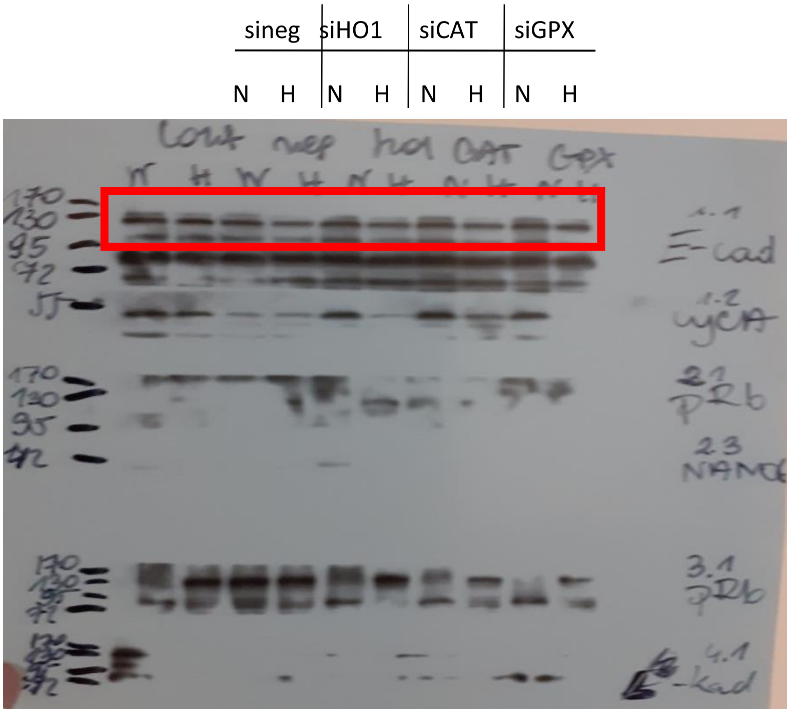

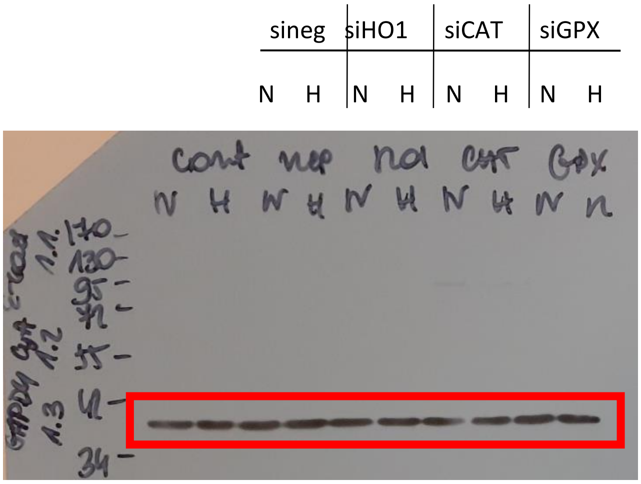

Fig. A.II.35

Snail

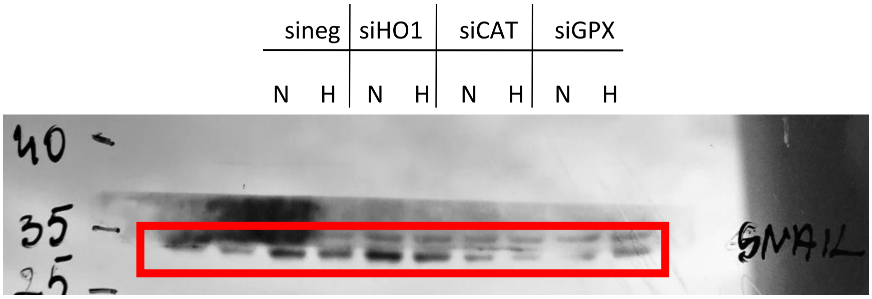

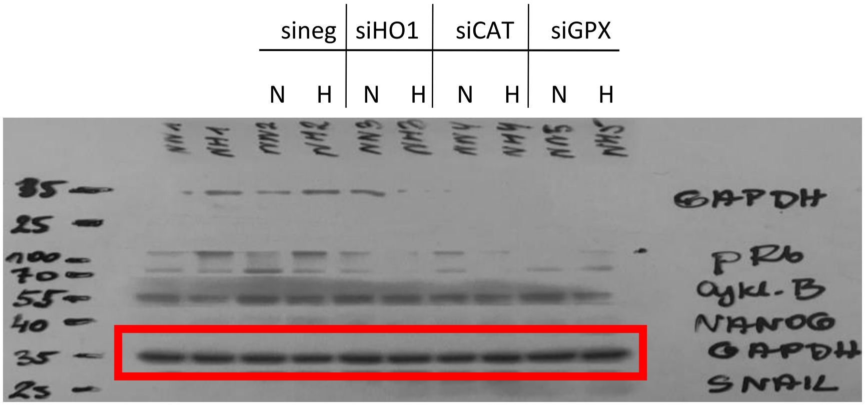

Fig. A.II.36

NANOG

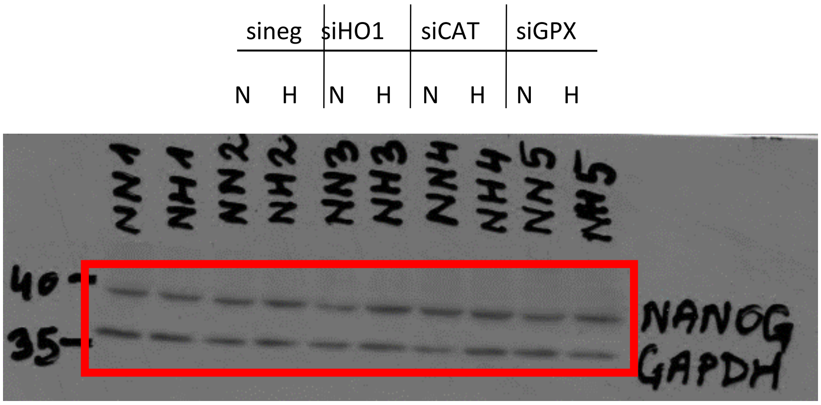

Fig. A.II.37

HO-1

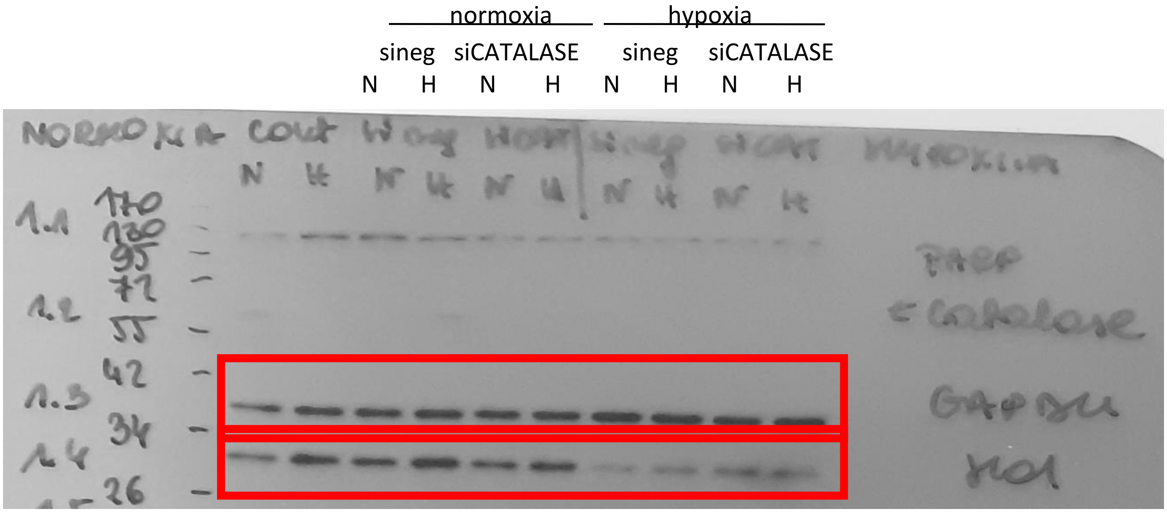

Fig. A.II.38

CAT

normoxia hypoxia

sineg siCATALASE sineg siCATALASE

N H N H N H N H

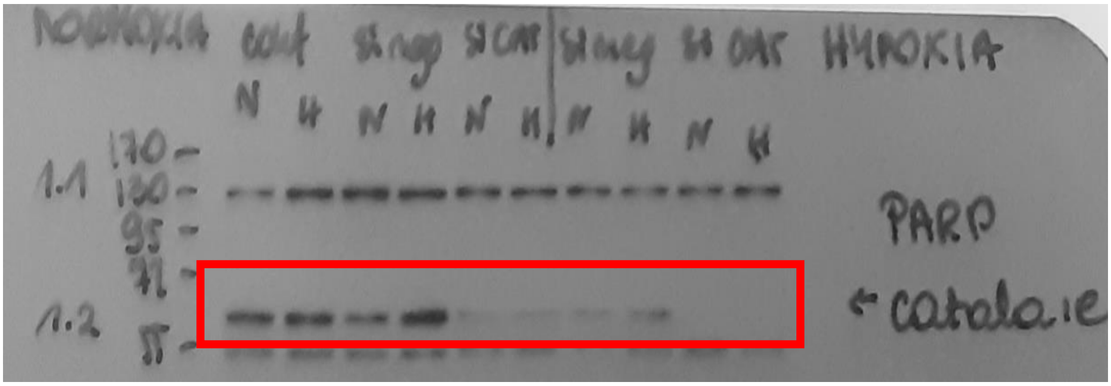

normoxia hypoxia

sineg siCATALASE sineg siCATALASE

N H N H N H N H

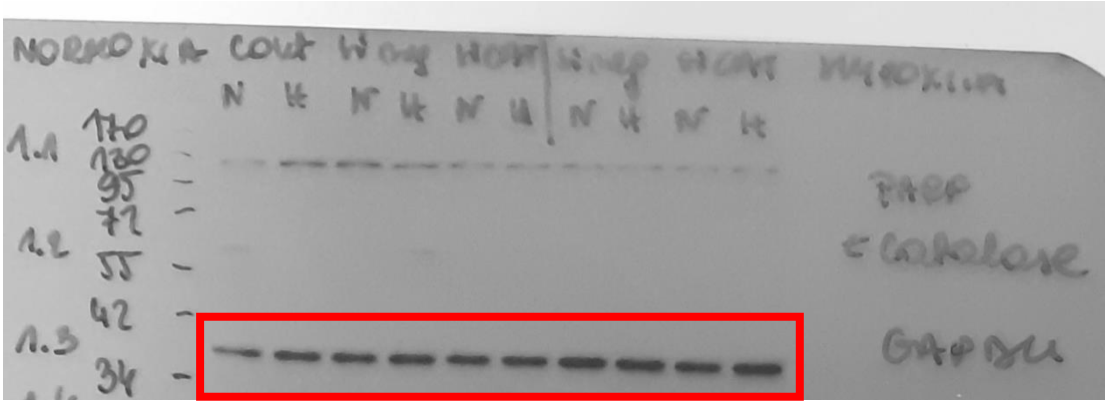

Fig. A.II.39

GPx-1

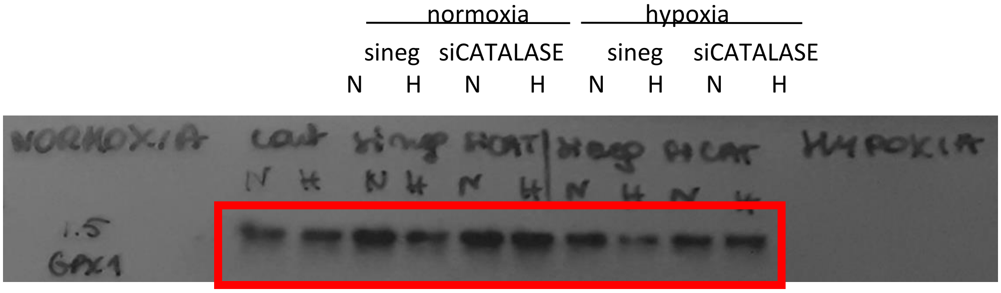

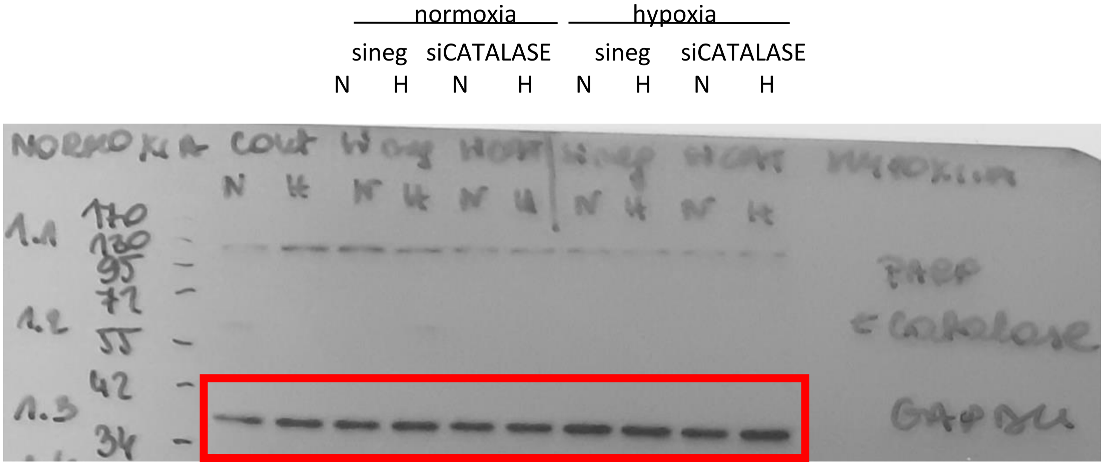

Fig. A.II.40

cyclin A

normoxia hypoxia

sineg siCATALASE sineg siCATALASE

N H N H N H N H

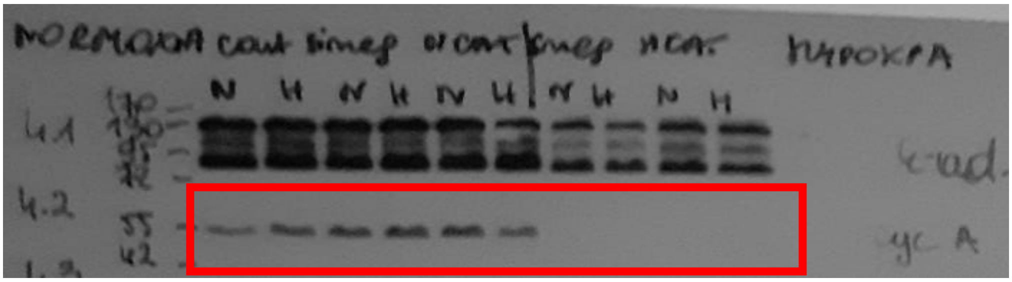

normoxia hypoxia

sineg siCATALASE sineg siCATALASE

N H N H N H N H

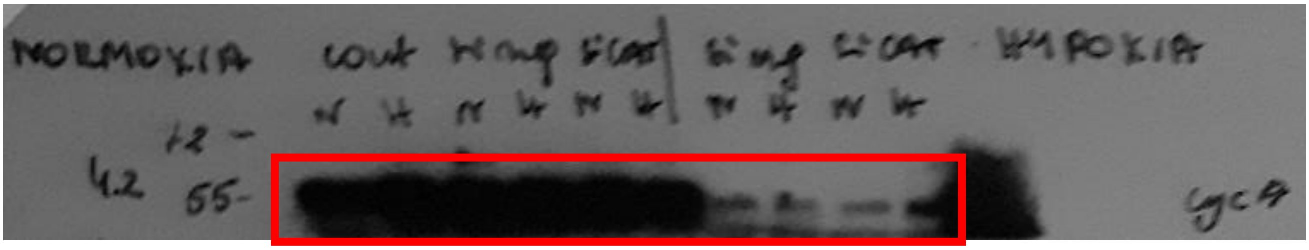

normoxia hypoxia

sineg siCATALASE sineg siCATALASE

N H N H N H N H

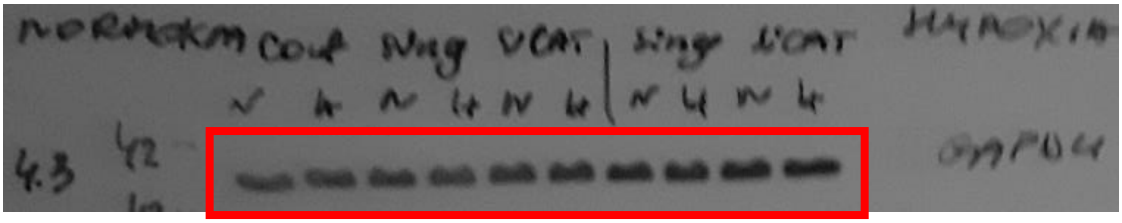

Fig. A.II.41

cyclin B

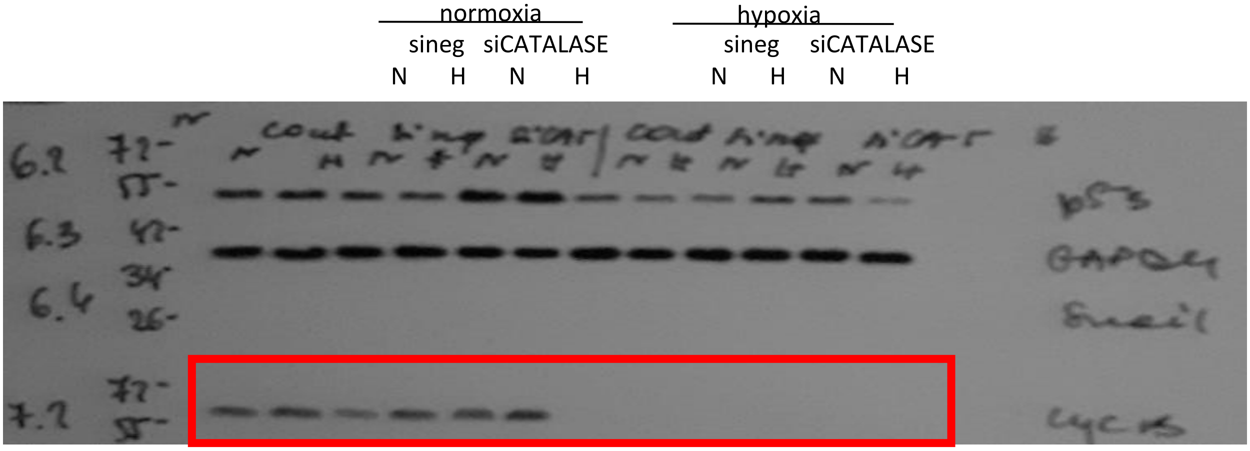

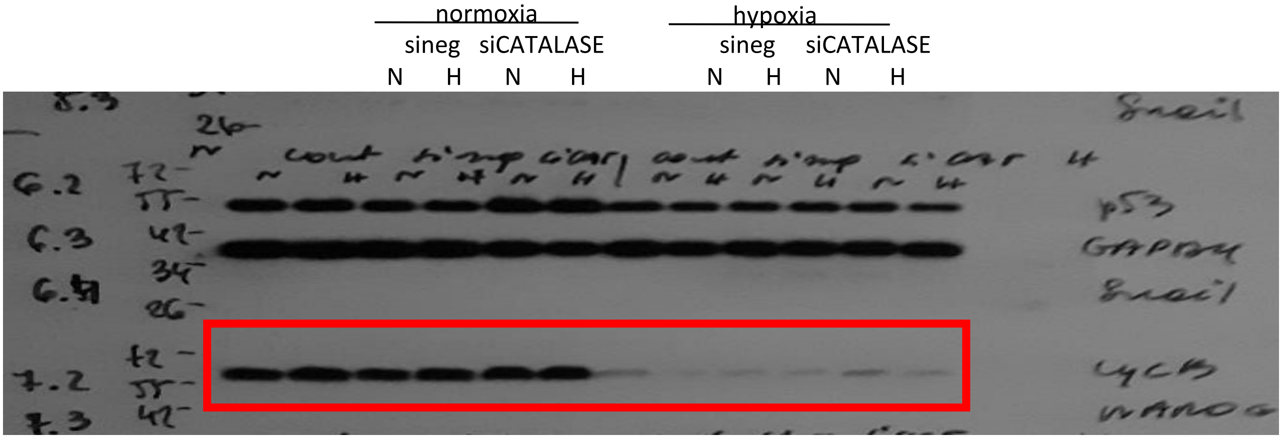

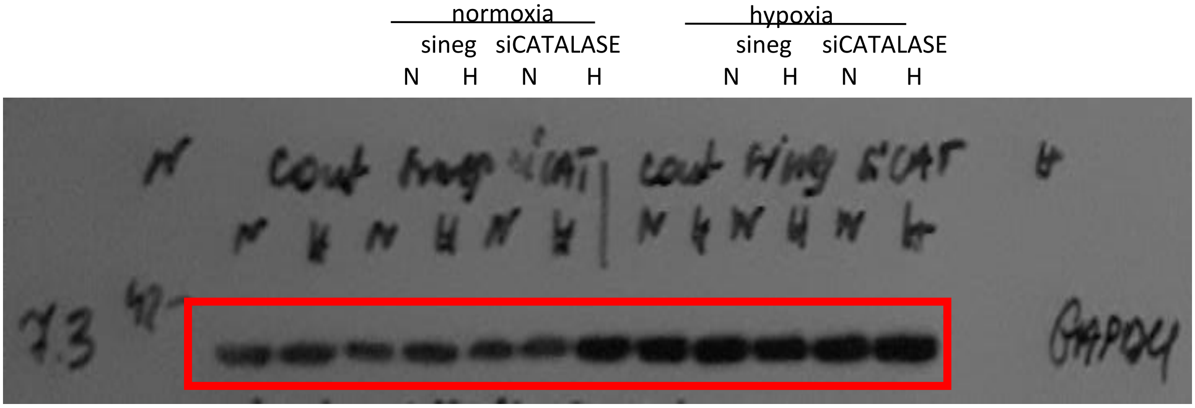

Fig. A.II.42

p-cdc2

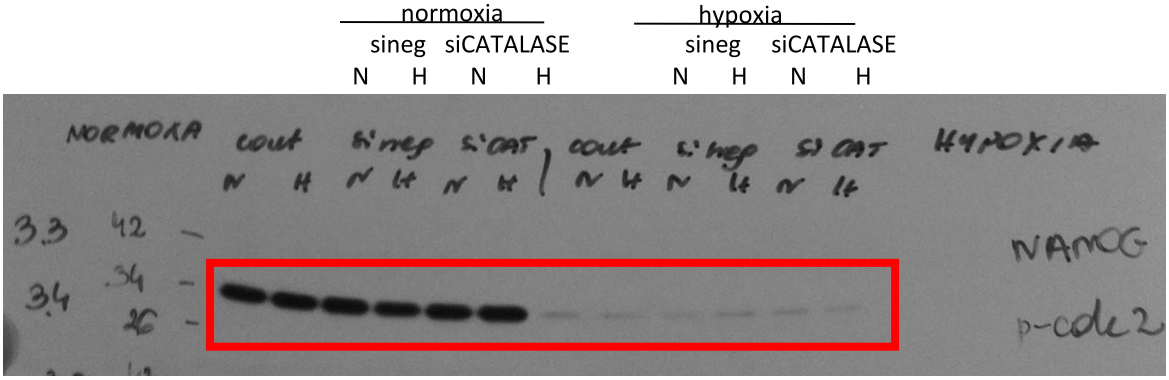

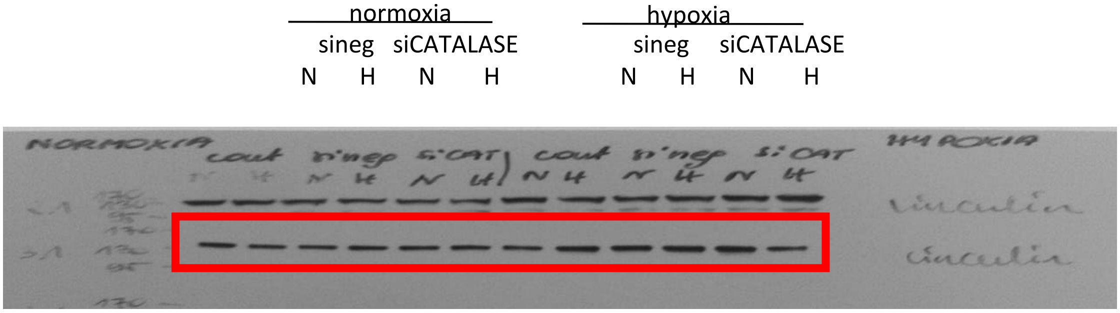

Fig. A.II.43

p-Rb

normoxia hypoxia
sineg siCATALASE sineg siCATALASE
N H N H N H N H

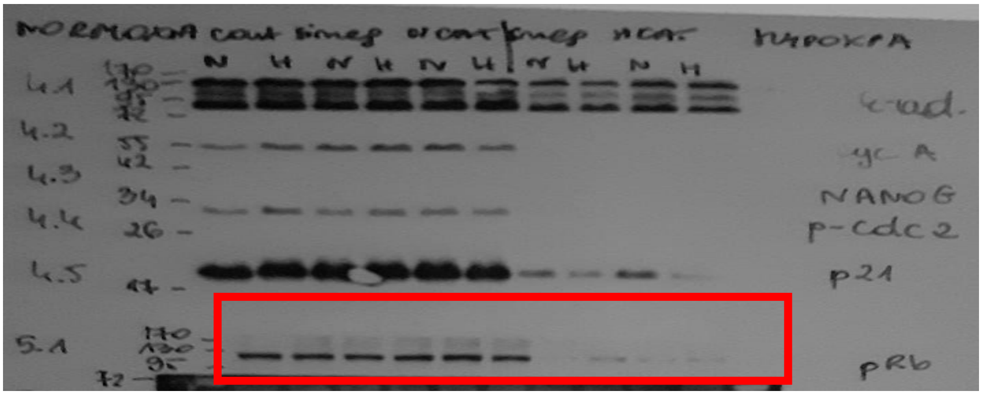

normoxia hypoxia
sineg siCATALASE sineg siCATALASE
N H N H N H N H

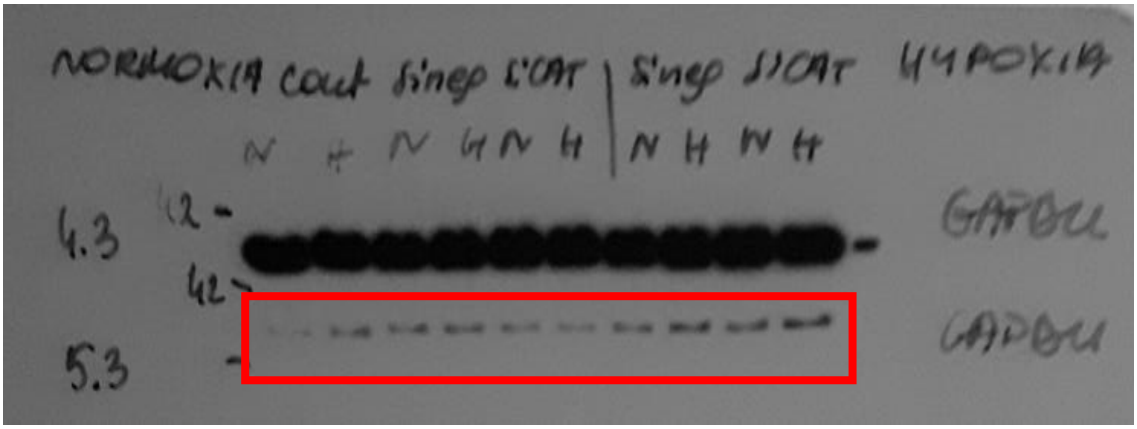

Fig. A.II.44

p21

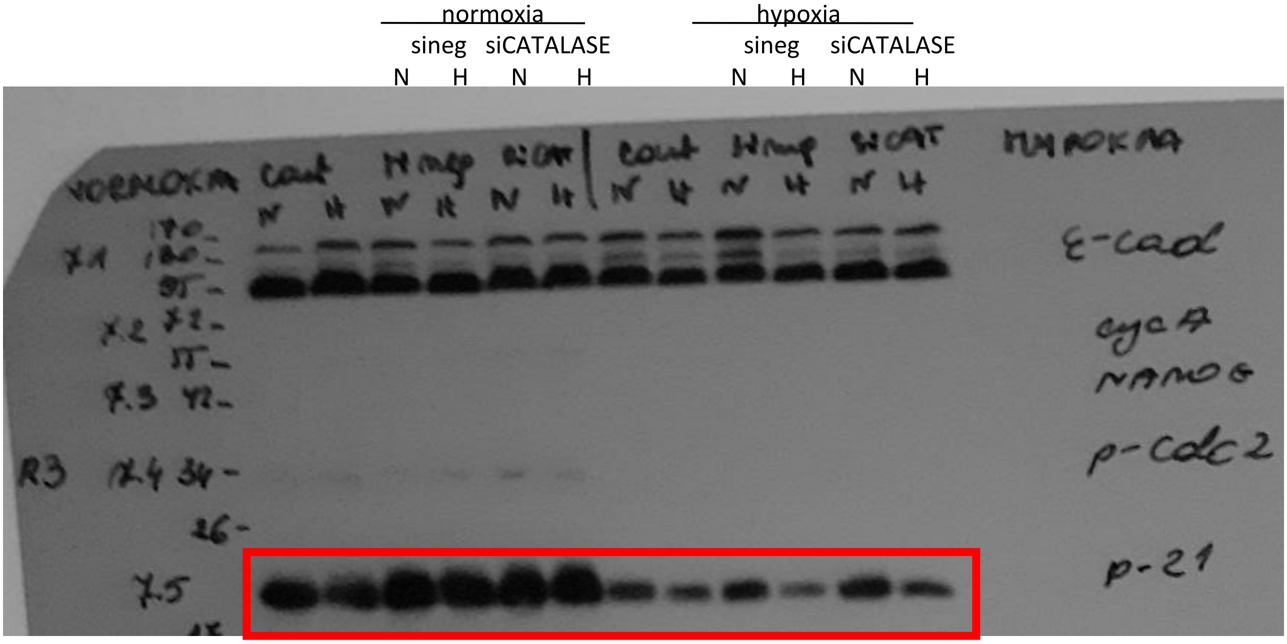

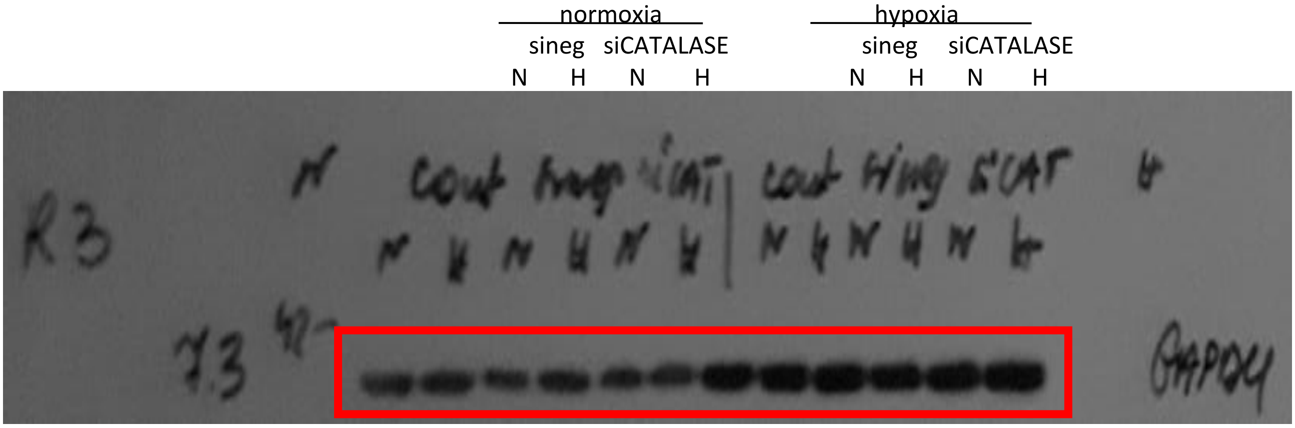

Fig. A.II.45

p-S6

normoxia | hypoxia

sineg siCATALASE sineg | sineg siCATALASE

N H N H | N H N H

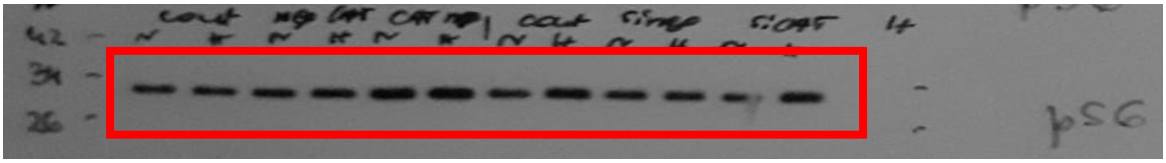

normoxia | hypoxia

sineg siCATALASE sineg | sineg siCATALASE

N H N H | N H N H

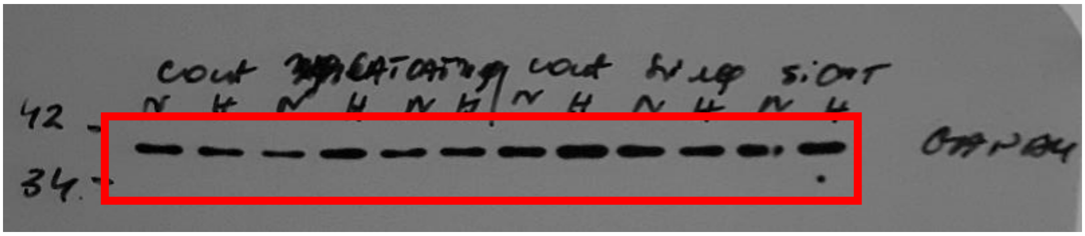

Fig. A.II.46

PARP-1

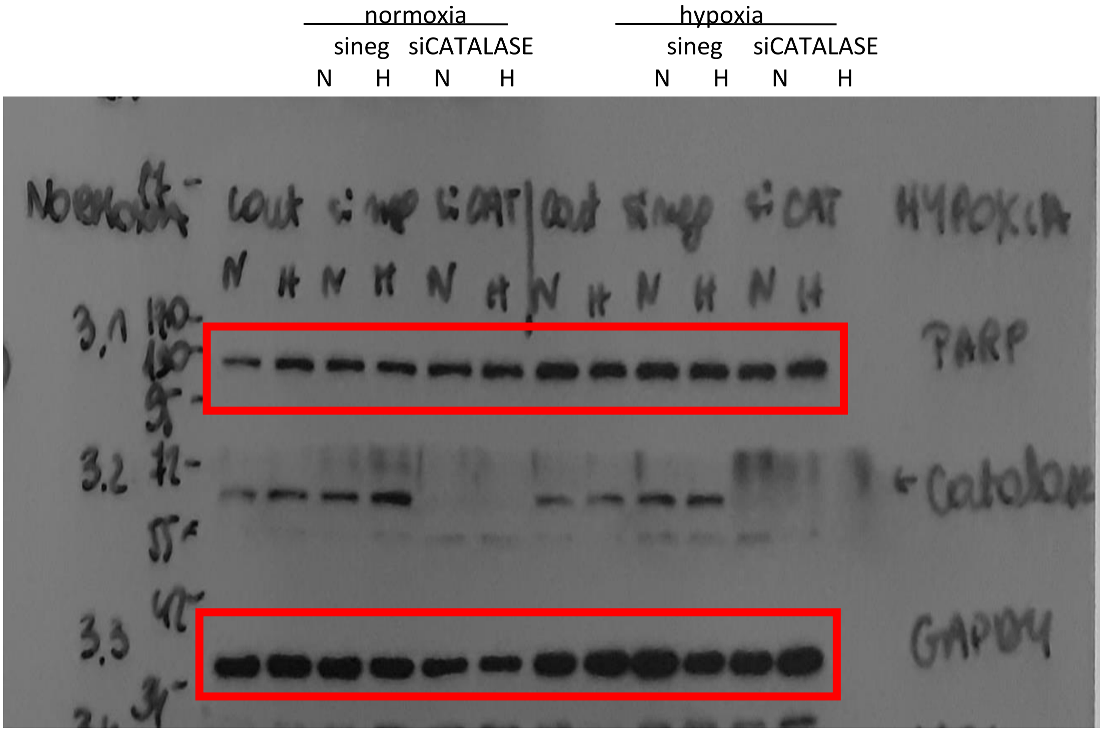

Fig. A.II.47

p53

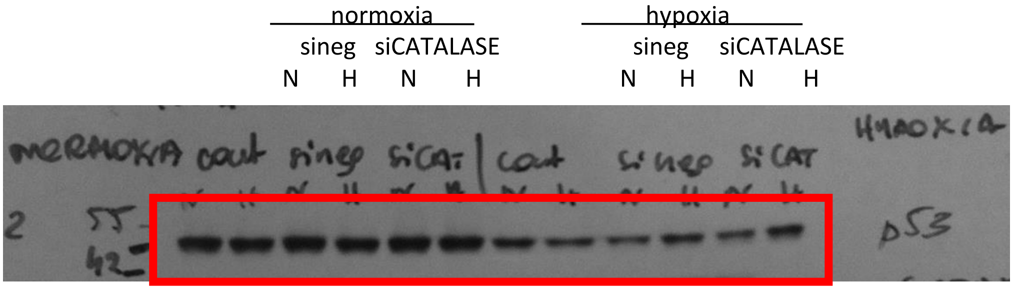

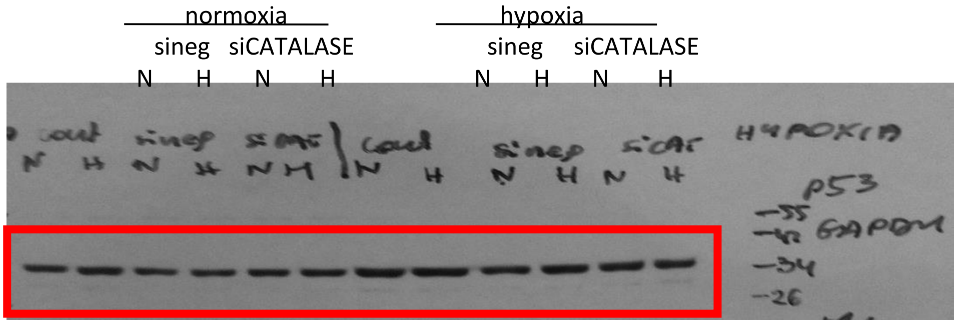

Fig. A.II.48

E-cadherin

normoxia hypoxia

sineg siCATALASE sineg siCATALASE

N H N H N H N H

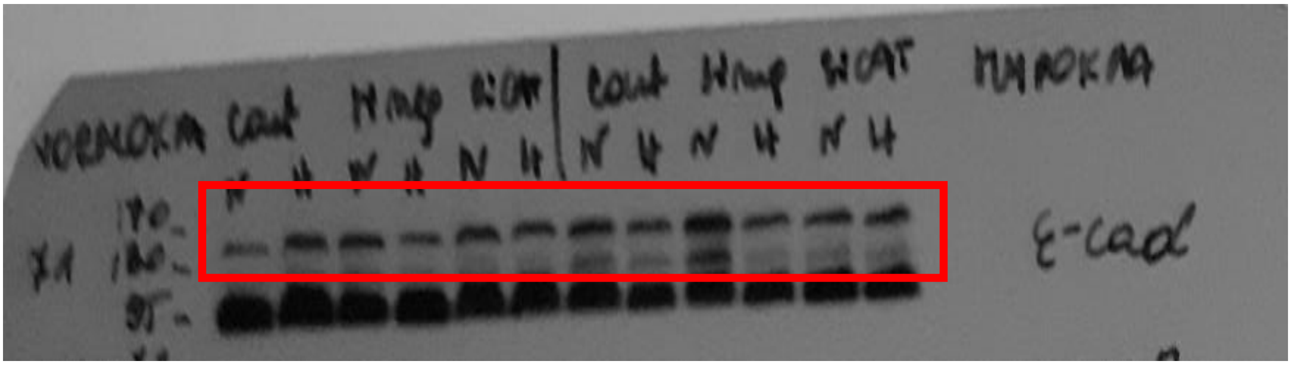

normoxia hypoxia

sineg siCATALASE sineg siCATALASE

N H N H N H N H

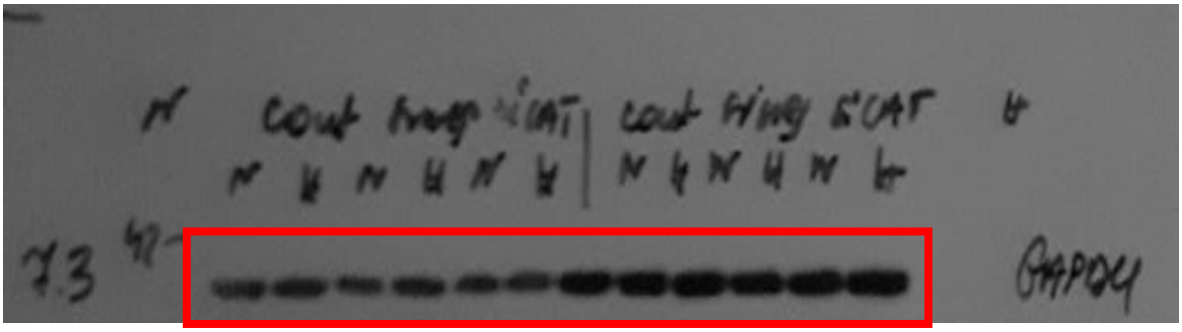

Fig. A.II.49

Snail

Snail

normoxia | hypoxia
sineg siCATALASE | sineg siCATALASE
N H N H | N H N H

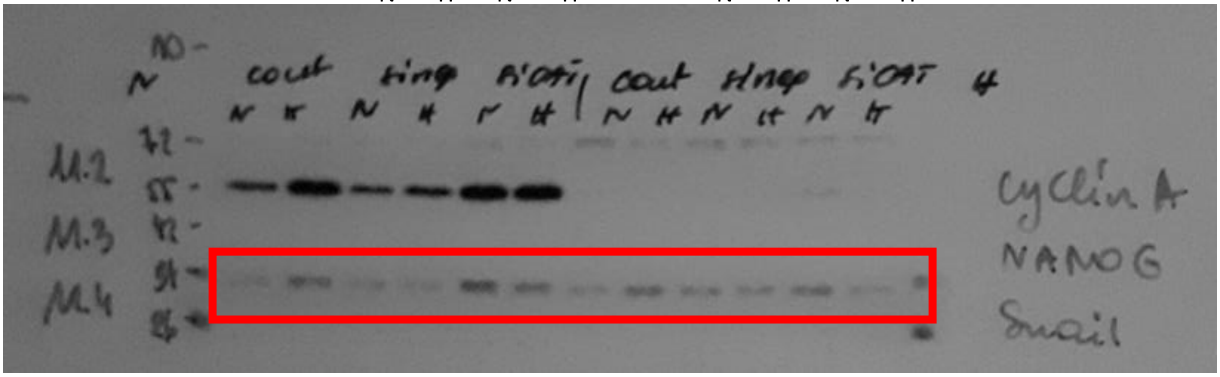

normoxia | hypoxia
sineg siCATALASE | sineg siCATALASE
N H N H | N H N H

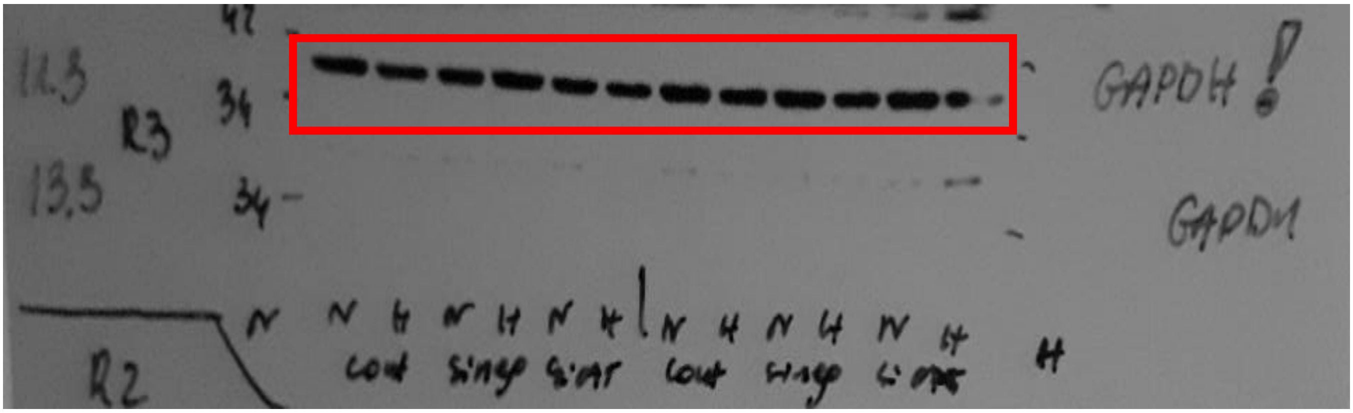

Fig. A.II.50

NANOG

normoxia — sineg, siCATALASE: N H N H
hypoxia — sineg, siCATALASE: N H N H

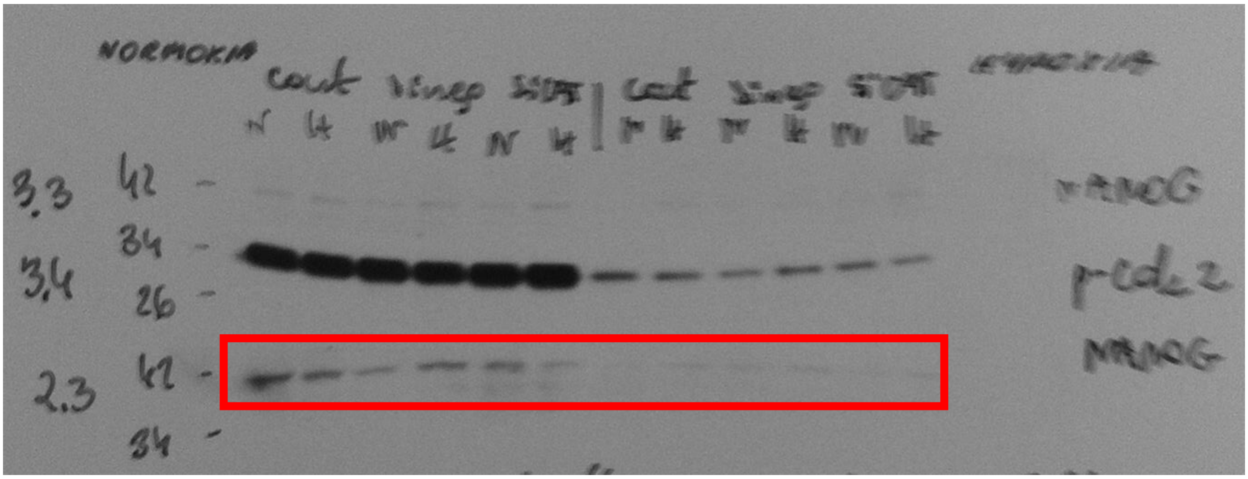

normoxia — sineg, siCATALASE: N H N H
hypoxia — sineg, siCATALASE: N H N H

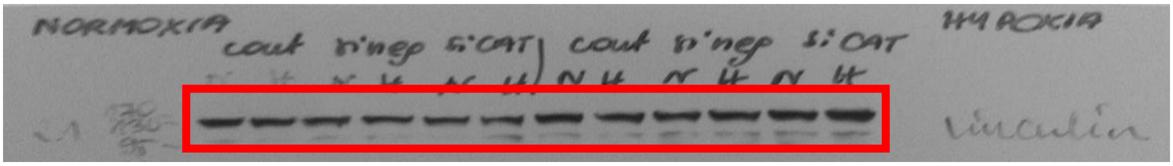

Fig. A.II.51
